# Search for ABCB1 Modulators Among 2-Amine-5-Arylideneimidazolones as a New Perspective to Overcome Cancer Multidrug Resistance

**DOI:** 10.3390/molecules25092258

**Published:** 2020-05-11

**Authors:** Aneta Kaczor, Márta Nové, Annamária Kincses, Gabriella Spengler, Ewa Szymańska, Gniewomir Latacz, Jadwiga Handzlik

**Affiliations:** 1 Department of Technology and Biotechnology of Drugs, Faculty of Pharmacy, Jagiellonian University Medical College, 9 Medyczna Street, 30-688 Kraków, Poland; aneta.kaczor@doctoral.uj.edu.pl (A.K.); ewa.szymanska@uj.edu.pl (E.S.); glatacz@cm-uj.krakow.pl (G.L.); 2 Department of Medical Microbiology and Immunobiology, Faculty of Medicine, University of Szeged, Dóm tér 10, H-6720 Szeged, Hungary; nove.marta@med.u-szeged.hu (M.N.); kincses.annamaria@med.u-szeged.hu (A.K.); spengler.gabriella@med.u-szeged.hu (G.S.)

**Keywords:** ABCB1 modulator, 5-arylideneimidazolone, Dimroth rearrangement, cancer MDR, Pgp, T-lymphoma cancer cell, rhodamine 123 accumulation assay, Pgp docking

## Abstract

Multidrug resistance (MDR) is a severe problem in the treatment of cancer with overexpression of glycoprotein P (Pgp, ABCB1) as a reason for chemotherapy failure. A series of 14 novel 5-arylideneimidazolone derivatives containing the morpholine moiety, with respect to two different topologies (groups A and B), were designed and obtained in a three- or four-step synthesis, involving the Dimroth rearrangement. The new compounds were tested for their inhibition of the ABCB1 efflux pump in both sensitive (parental (PAR)) and ABCB1-overexpressing (MDR) T-lymphoma cancer cells in a rhodamine 123 accumulation assay. Their cytotoxic and antiproliferative effects were investigated by a thiazolyl blue tetrazolium bromide (MTT) assay. For active compounds, an insight into the mechanisms of action using either the luminescent Pgp-Glo™ Assay in vitro or docking studies to human Pgp was performed. The safety profile in vitro was examined. Structure–activity relationship (SAR) analysis was discussed. The most active compounds, representing both 2-substituted- (**11**) and Dimroth-rearranged 3-substituted (**18**) imidazolone topologies, displayed 1.38–1.46 fold stronger efflux pump inhibiting effects than reference verapamil and were significantly safer than doxorubicin in cell-based toxicity assays in the HEK-293 cell line. Results of mechanistic studies indicate that active imidazolones are substrates with increasing Pgp ATPase activity, and their dye-efflux inhibition via competitive action on the Pgp verapamil binding site was predicted in silico.

## 1. Introduction

Multidrug resistance (MDR) is nowadays one of the most important problems in the treatment of various diseases including cancer. The lack of efficacy seen after usage of chemotherapeutic drugs, effective previously, is connected to various mechanisms. One of them involves an increased drug efflux through membrane transporters, e.g., P-glycoprotein (Pgp, ABCB1) [1,2,3,4]. Pgp is an ATP-binding cassette protein, encoded by the *MDR1* (*ABCB1*) gene, which is present in both normal and cancer cells [5,6]. The structure includes two hydrophobic transmembrane domains, which contain drug binding domain (DBD). In the cytoplasmic region, there are two nucleotide binding domains (NBDs) in which coupling ATP hydrolysis takes place [5,7,8,9]. ABCB1 obtains energy from the ATP hydrolysis process and uses it to translocate to the cell exterior structurally unrelated compounds, e.g., chemotherapeutics, xenobiotics [10,11]. Hence, ABCB1 overexpression became the reason for the lack of response in the applied therapy, due to lowered drug concentration, often below the therapeutic point [1,2,3,4,12]. This mechanism seems to play an important role in cancer MDR, mostly because many anticancer drugs are ABCB1 substrates, e.g., doxorubicin, paclitaxel, vincristine [13]. Additionally, cells with overexpression of this pump may inhibit the process of apoptosis [14]. This may lead to resistance even against chemotherapeutics, which are not Pgp substrates [6]. In this context, searching for ABCB1 inhibitors seems to be a prominent way to overcome cancer MDR [15]. Earlier studies provided four main groups of Pgp inhibitors, with respect to their potency, selectivity, and potential of drug–drug interaction [16]. However, the toxicity of previously found ABCB1 inhibitors is a significant problem that limits their therapeutic application [12]. For example, verapamil, which belongs to the first group, demonstrates cardiotoxicity due to the higher affinity for the calcium channel than for ABCB1 [17]. Taking into account the important role of MDR in cancer, searching for new compounds that are able to increase the concentration of chemotherapeutic drugs is currently a crucial task of cancer medicinal chemistry [12].

**Figure 1:**
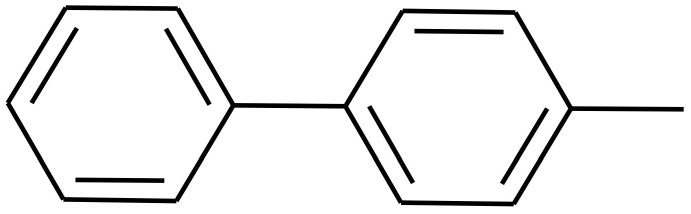
5-Arylideneimidazolones able to block mechanism of bacterial multidrug resistance (MDR) (**1**,**2**) [21,22].

On the other hand, compounds with the hydantoin (imidazole-2,4-dione) scaffold are a big structural family in the search for new drugs with various therapeutic directions, to give active agents with antiarrhythmic, anti-inflammatory, or other physiological properties [18,19,20]. Representatives from this group displayed potential activity as antibiotic adjuvants able to block mechanism(s) of bacterial MDR, e.g., efflux pumps (compounds **1**, **2**, Figure 1) [21,22].

**Figure 2:**
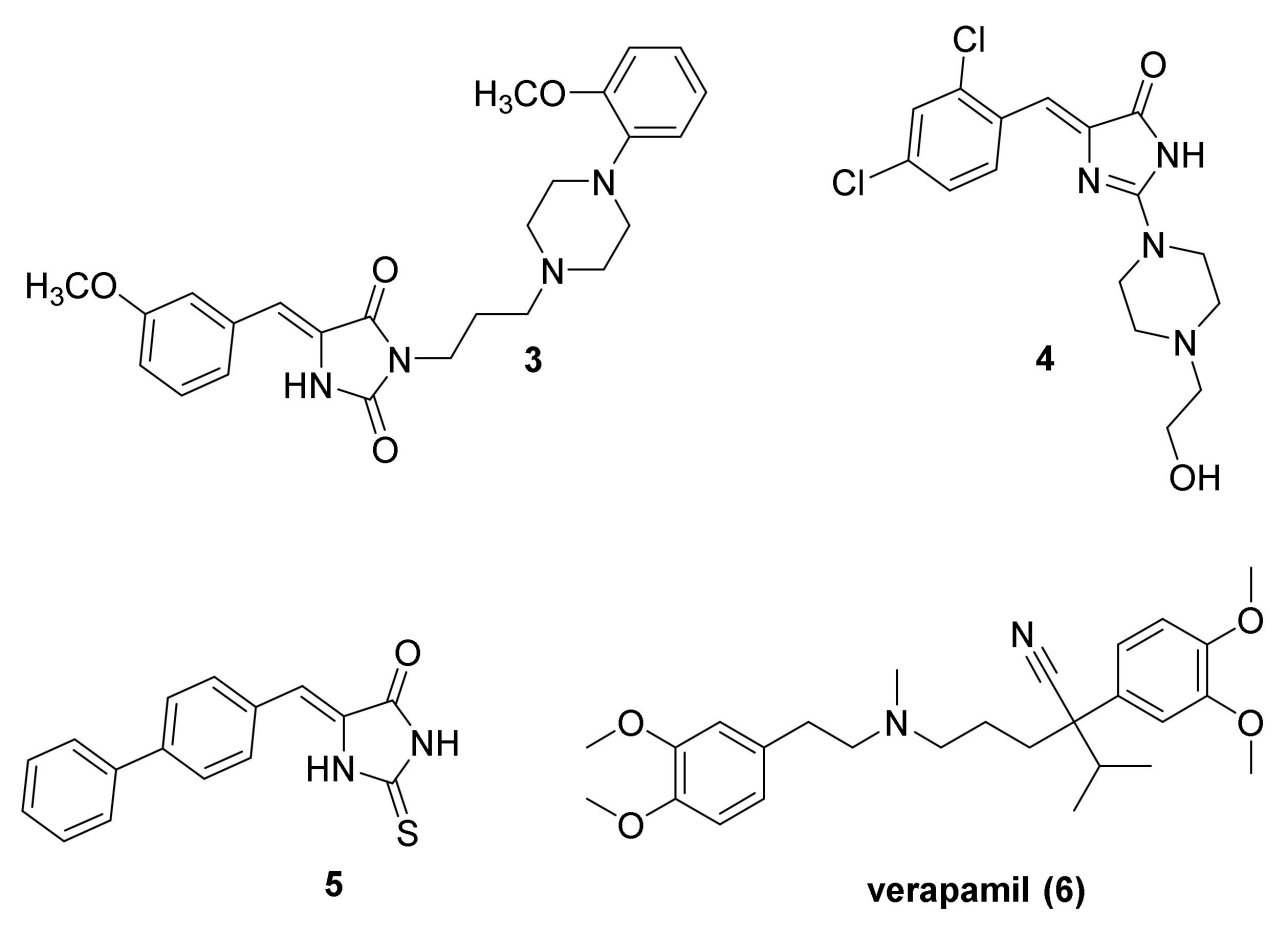
Previously found (thio)hydantoin derivatives able to modulate ABCB1 in cancer cells (**3**–**5**) and the reference modulator, verapamil (**6**) [23,24].

Furthermore, some (thio)hydantoin derivatives (**3**–**5**; Figure 2) demonstrated the ability to modulate the ABCB1 efflux pump in cancer cells at the same or stronger level than verapamil (**6**; Figure 2) [23,24].

SAR analysis performed within the previous studies indicated that the arylidene moiety at position 5 of imidazolone was crucial for the modulatory action, while the presence of hydroxyl groups was unprofitable [24].

Taking this into account, a series of new 5-arylideneimidazolones (**7**–**20**) were designed, synthesized, and tested as ABCB1 modulators in mouse T-lymphoma cells within this study. The designed compounds possess amine fragments, containing a morpholine moiety, at position 2 (group A, Table 1) or at positions 2 and 3 (group B, Table 1) of the imidazolone ring. In order to determine MDR efflux pump modulatory properties for the new compounds, a dye-substrate accumulation assay was performed in two T-lymphoma cell lines, i.e., parental (PAR) and multidrug resistant one (MDR) with overexpression of ABCB1. The cytotoxicity and antiproliferative effect of the obtained compounds were examined in these cell lines. In search for probable molecular mechanisms of modulatory action of active compounds, both in silico and in vitro studies were carried out. The safety of the most active compounds was tested in vitro in the human HEK-293 cell line.

## 2. Results

### 2.1. Synthesis

**Scheme 1:**
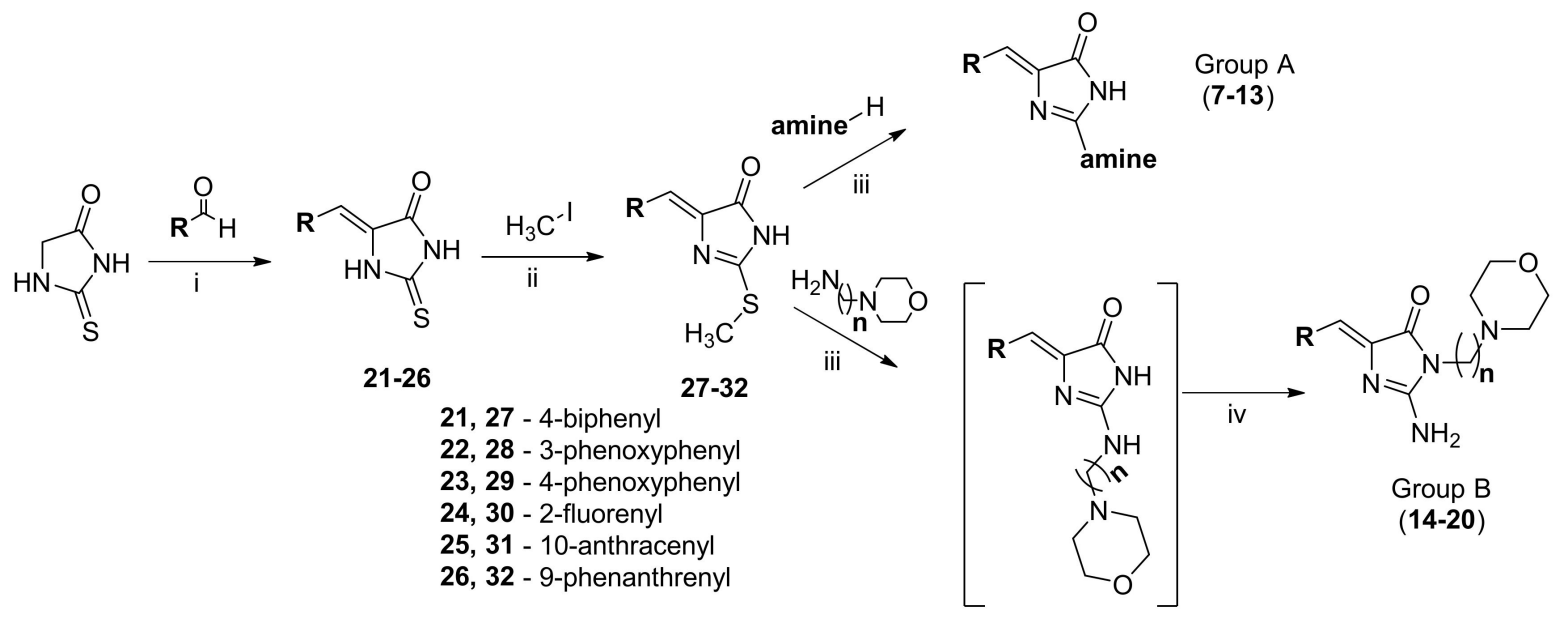
Synthesis route for compounds **7**–**20**; (i), CH_3_COONa, CH_3_COOH, reflux, 5–7 h; (ii) CH_3_I, C_2_H_5_ONa, rt, 24 h; (iii) proper amine, oil bath 120–130 °C, 15 min; C_2_H_5_OH, reflux 5–7 h (iv) Dimroth rearrangement.

The compounds (**7**–**20**) were synthesized within 3–4-step synthesis pathways, according to Scheme 1. The synthesis of compounds **16** and **19** is described elsewhere [22]. In the first step, Knoevenagel condensation of thiohydantoin with suitable aromatic aldehydes was applied to give 5-arylideneimidazolone intermediates (**21**–**26**) that were subsequently reacted with iodomethane in sodium ethanolate solution. Treatment of the resulting S-methylated derivatives (**27**–**32**) with commercial secondary- (Group A, Table 1) or primary- (Group B, Table 1) amines allowed the target 2-amine-5-arylideneimidazolones (**7**–**20**) to be yielded. In the case of group B (**14**–**20**), the Dimroth rearrangement was observed to proceed via a similar mechanism to that described previously [22]. Thus, the final products of group B contain the primary amine group at position 2 and the morpholine-alkyl moiety at position 3 of the imidazolone core.

### 2.2. Biological Screening

#### 2.2.1. The Rhodamine 123 Accumulation Assay

Compounds **7**–**20** were investigated for their ABCB1 efflux pump inhibiting activity using the rhodamine 123 accumulation assay. The study was carried out on mouse T-lymphoma cell lines, including the parental sensitive line (PAR) and its multidrug-resistant subline (MDR) transfected with the human *MDR1* (*ABCB1*) gene. Results were acquired by flow cytometry measuring the intracellular accumulation of the fluorescent ABCB1 substrate rhodamine 123. The fluorescence activity ratio (FAR), as a measure of the efflux pump modulating properties, was calculated based on the fluorescence data obtained [23,24,25]. Verapamil was used as a reference ABCB1-modulator with respect to two assays performed for appropriate compounds (superscript 1 or 2, Table 2). Therefore, results were also expressed as % of FAR for verapamil in the appropriate assay (quotient, Table 2) in order to enable a good comparison of results within the whole series [26].

Results indicate that the arylideneimidazolones **8**, **12**, **13**, and **15**–**17** demonstrated slight to moderate (**12**) ABCB1 modulatory properties (FAR = 2.07–6.05). Two compounds (**11**, **18**), however, were highly effective, almost 1.5 fold more active than verapamil tested at the same concentration (20 μM). The fluorescence activity ratio (FAR) for compound **11** was 23.419, with a quotient of 146.22%, while compound **18** displayed 138.37% of the activity determined for verapamil. At 10 fold lower concentration (2 μM), the whole series (**7**–**20**) was not active.

#### 2.2.2. Cancer Cytotoxicity Assays

The cytotoxicity of compounds **7**–**20** was examined in both aforementioned PAR and MDR mouse T-lymphoma cell lines, using the MTT assay to estimate IC_50_ values. The selectivity index for cytotoxic action on MDR with respect to PAR cells was calculated (SI, Table 3). The morpholine-imidazolones (**7**–**20**) showed weak (IC_50_ > 20 µM) to moderate (10 µM < IC_50_ < 20 µM) cytotoxic effects, and all derivatives were, furthermore, more potent in PAR cells, as shown by the selectivity index (SI < 1). This range of SI may indicate ABCB1-efflux pump substrate properties for the whole series (**7**–**20**), especially distinct in the case of **19** (SI ≤ 0.29), and slightly weaker for **8**, **12**, **16**, and **18**.

The relatively strongest T-lymphoma cytotoxic action could be observed in the case of compound **9** with IC_50_ values of 12.82 µM (PAR) and 18.11 µM (MDR). The rest of the compounds displayed IC_50_ > 20 µM in the MDR cells, and 15.80 µM < IC_50_ < 20 µM in the case of the most active ones (**12**, **15**, **16**, **18**, and **20**) in parental T-lymphoma cells.

#### 2.2.3. Influence on Cancer Cell Proliferation

Compounds (**7**–**20**) were also tested for their ability to inhibit proliferation of both PAR and MDR mouse T-lymphoma cancer cells. The compounds (**7**–**20**) displayed moderate to weak antiproliferative effects (Table 4). Compared to the cytotoxicity results, the effects were even stronger, with IC_50_ values in the range of 7.78 µM (**9**) to 26.42 µM (**10**) in PAR cells, 10.82 µM (**18**) to 48.58 µM (**13**) in the case of MDR. Compound **18** was the most active agent, with an IC_50_ of 10.82 µM in the multidrug-resistant subline of mouse T-lymphoma cells. Half of the tested compounds exhibited an IC_50_ lower than 20 µM in MDR cells. The antiproliferative effects were 28–94 fold weaker than those of the reference anticancer drug (doxorubicin) in PAR, and 6–28 fold in the resistant cells. Interestingly, the relatively most active agent (**18**) showed a higher antiproliferative potency in MDR cells compared to the parental ones. A similar trend was also observed for **10**, whereas the antiproliferative action was comparable in both PAR and MDR cells for compounds **11** and **16**.

### 2.3. Insight into Molecular Mechanisms of the Efflux Pump Modulation

Three compounds with the best efflux pump modulatory properties, i.e., two strong agents **11** and **18** and the moderate one (**12**), were selected for a deeper insight into mechanisms of action at the molecular level, using both in vitro and in silico assays. The ABCB1-efflux pump (Pgp) as an example of a transport protein represents a complex mechanism. Thus, two main mechanisms for the action of modulators can be involved, including: (i) a consumption of energy coming from ATP hydrolysis, which involves ATPase and is necessary to activate the Pgp pump to perform substrate efflux, and (ii) a competitive modulator-substrate action to obtain the pump binding site, which protects the substrate to be expelled, although the pump is active. In order to estimate the first mechanism, the arylideneimidazolones were tested in the intrinsic activity Pgp ATPase assay in vitro, whereas their action on the Pgp-binding site was examined on the basis of molecular modeling.

#### 2.3.1. Intrinsic Activity toward ABCB1 In Vitro

Compounds **11**, **12**, and **18** were tested at 100 µM on their influence on the ATPase activity of the Pgp pump in the luminescence Pgp-Glo™ Assay, according to previously described methods and protocols [27,28,29]. In this assay, luminescence was measured. The Pgp-dependent decreases in luminescence reflect the ATP consumption by Pgp. The ABCB1 basal ATP consumption (basal activity) is considered a difference between the luminescent signal of samples treated with Na_3_VO_4_, the selective and strong Pgp inhibitor (100% inhibition observed), and the luminescence of untreated Pgp samples. Thus, inhibitors give results <100% of the basal activity, while stimulators/substrates cause a statistically significant increase in the basal activity. Verapamil at 200 µM was used as a reference modulator with the substrate/ATPase stimulatory mode of action, and caffeine was used to give a negative control as a reference inactive compound toward the ABCB1 transporter.

**Figure 3:**
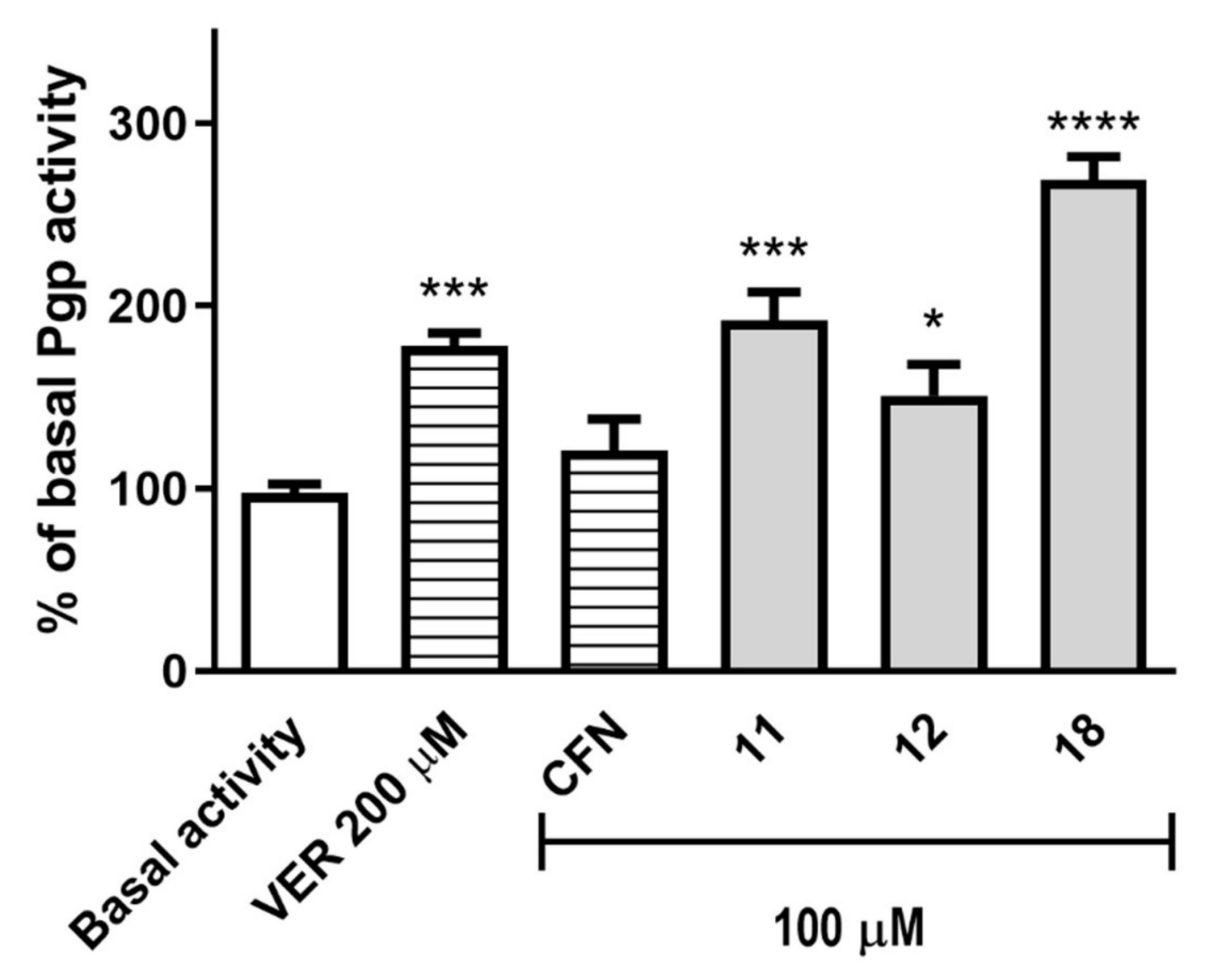
The effect of the ABCB1 substrate verapamil (VER) (200 μM), ABCB1-negative compound caffeine (CFN), and compounds **11**, **12**, and **18** (100 μM) on ABCB1 basal activity. The compounds are recognized as ABCB1 substrates if they stimulate its basal activity (>100%). Data are presented as the mean ± SD. Statistical significance was evaluated by one-way ANOVA, followed by Bonferroni’s comparison test (* *p* < 0.05, *** *p* < 0.001, **** *p* < 0.0001 compared to the basal activity).

Compounds **11** and **18** caused a statistically significant (*p* < 0.001 and *p* < 0.0001, respectively) increase in basal activity in the range corresponding to verapamil (**11**) or higher (**18**). Compound **12** also increased the basal activity with the statistical significance (*p* < 0.05); however, the action was much weaker than those observed for **18**, verapamil, and **11**, and only slightly stronger than that of the negative control caffeine (Figure 3).

This trend of action is in some accordance with the modulatory potency of the compounds found in the rhodamine 123 accumulation assays (**11**–**18** >> **12**, Table 2), but also excludes an ATPase-involving Pgp inhibitory action of the imidazolones (**11**, **12**, and **18**), indicating their Pgp-substrate properties. These results suggest that the most probable mechanism of the inhibition of rhodamine 123 efflux, observed for the compounds in the accumulation assay, is based on the modulator-substrate competitive action to the binding site of ABCB1.

#### 2.3.2. Molecular Modeling

In order to support the mechanism hypothesis postulated on the basis of results for both the Pgp ATPase and the rhodamine 123 accumulation assays, possible binding modes of the arylideneimidazolone compounds at the human Pgp protein were investigated using the molecular docking method.

##### Homology Model of Human Pgp

The homology model of human Pgp was constructed by multiple comparative modeling, and three reported X-ray structures of murine Pgp in the inward facing conformation: 4Q9H, 4XWK, and 4M1M, were selected as templates due to the relatively high diffraction resolution. To improve the quality of the model, the secondary structure of the linker region (amino acids 630–698) was additionally calculated.

##### Docking Results Analysis

Structures of the three selected imidazolone modulators **11**, **12**, and **18**, as well as verapamil, were subjected to induced fit docking to the predicted homology model. Obtained top-ranked poses were carefully analyzed in order to assess the possibility of competition between verapamil and imidazolone derivatives for occupation of the same binding site or overlapping binding sites in Pgp.

The highly hydrophobic transmembrane interior of Pgp is believed to be the putative site for substrate recognition [30,31,32]. According to the literature data, the residues inside this cavity are neutral, devoid of a positive or negative charge, and the substrates bind to the protein mainly by means of hydrophobic interactions and van der Waals and hydrogen bonds [31]. Most of the published data report docking of ligands at their neutral form; however, some authors also describe the docking of positively charged species [33,34,35]. For these reasons, we decided to perform docking to the homology model for both neutral and positively charged forms of the ligands in separate runs. The protonation states at pH = 7±2 were assigned during ligand preparation. In the case of predicted ionized forms of the imidazolone derivatives (**11**, **12**, and **18**), the positive charge was located on the nitrogen atom of the morpholine moiety.

**Figure 4:**
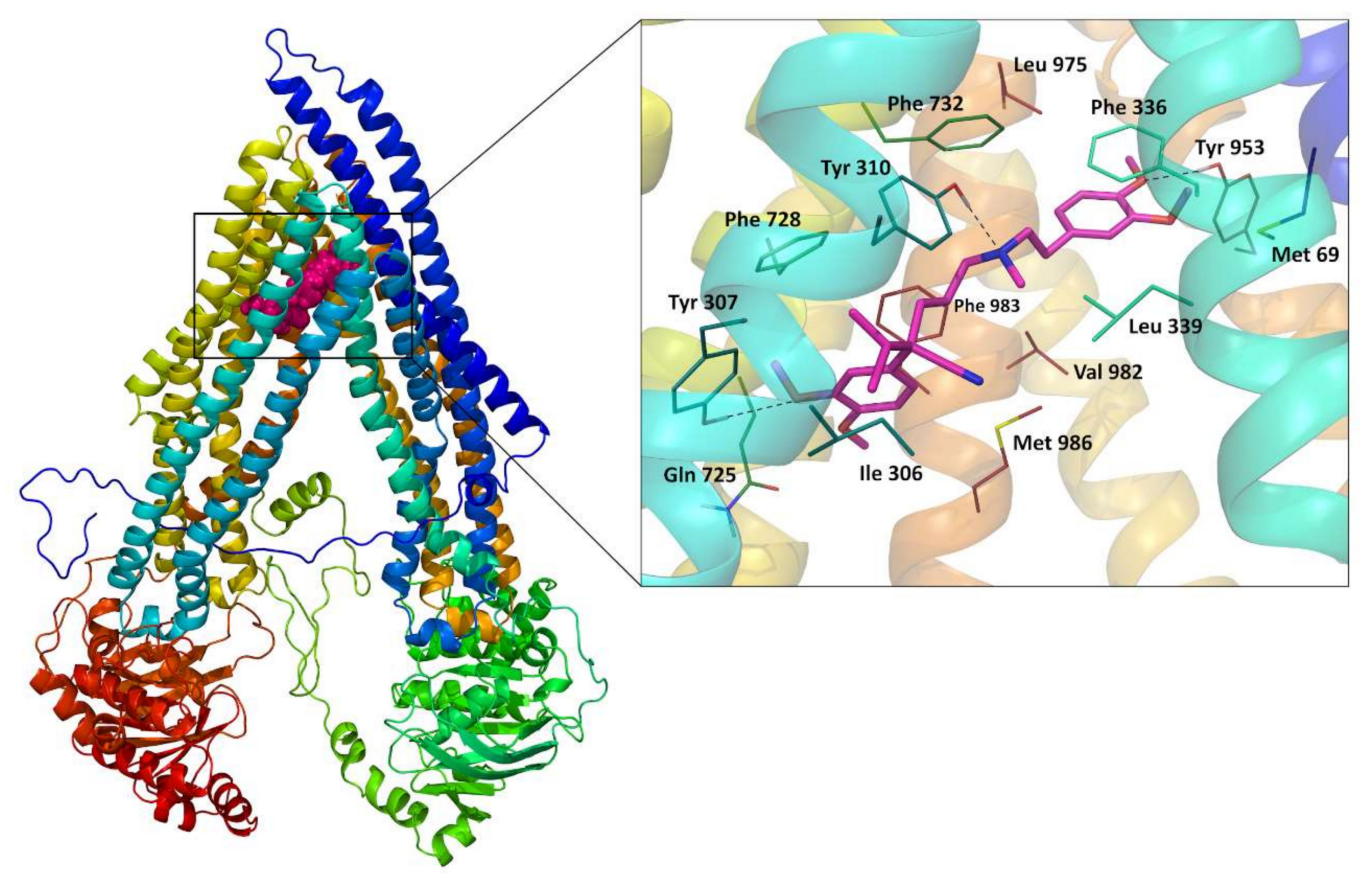
On the left: Position of the top-ranked docking pose of verapamil in human Pgp homology model; On the right: Binding mode and molecular interactions observed for the top-ranked docking pose of verapamil.

The binding space for verapamil was generated as a box of 25 Å size, defined by a centroid of amino acids found in the cysteine-scanning mutagenesis studies to be a part of the verapamil binding site [36,37,38]. Docking results for the neutral and charged forms of this drug showed similar poses for both species with comparable values of the binding energy expressed by the IFD score (Table S1 in Supplementary). As a reference for further investigations, one of the top-ranked poses (with a docking score −7.471 for the neutral form of verapamil) was chosen for its very good agreement with experimental data. The binding mode of verapamil in this pose follows the pattern of protein–ligand interactions predicted by other authors [32,34,39,40,41]. The molecule of the ligand is located in the surroundings, including mostly hydrophobic residues: Met 69, Phe 72, Ile 306, Phe 335, Phe 336, Leu 339, Leu 724, Phe 728, Phe 732, Leu 762, Phe 957, Leu 975, Phe 978, Phe 983, and Met 986 (Figure 4). Benzene rings of the drug were found to interact with Phe 983 and Phe 336 by π–π stacking. Furthermore, three hydrogen bonds were observed in this binding mode: two H-bonds formed by methoxy groups interacting with Tyr 307 and Tyr 953, and a H-bond between Tyr 310 and the amine group of verapamil.

For the investigated 5-arylideneimidazolone derivatives **11**, **12**, and **18**, the receptor grid box was set to include the whole binding cavity formed by the transmembrane region of the human Pgp, as defined for the P08183 sequence in the UniProt database [42]. Analysis of IFD docking results showed that almost all of the obtained top-ranked poses for the neutral forms of compounds were located at the top of the cavity (close to the outer leaflet of the membrane), interacting with residues of TM1, TM2, TM5, TM6, TM7, TM8, TM9, TM11, and TM12. In contrast to the case of verapamil, docking scores, XP Glide scores, and IFD scores demonstrated by the docked protonated forms of imidazolones were significantly lower than those seen for the neutral forms; therefore, we did not investigate them further.

**Figure 5:**
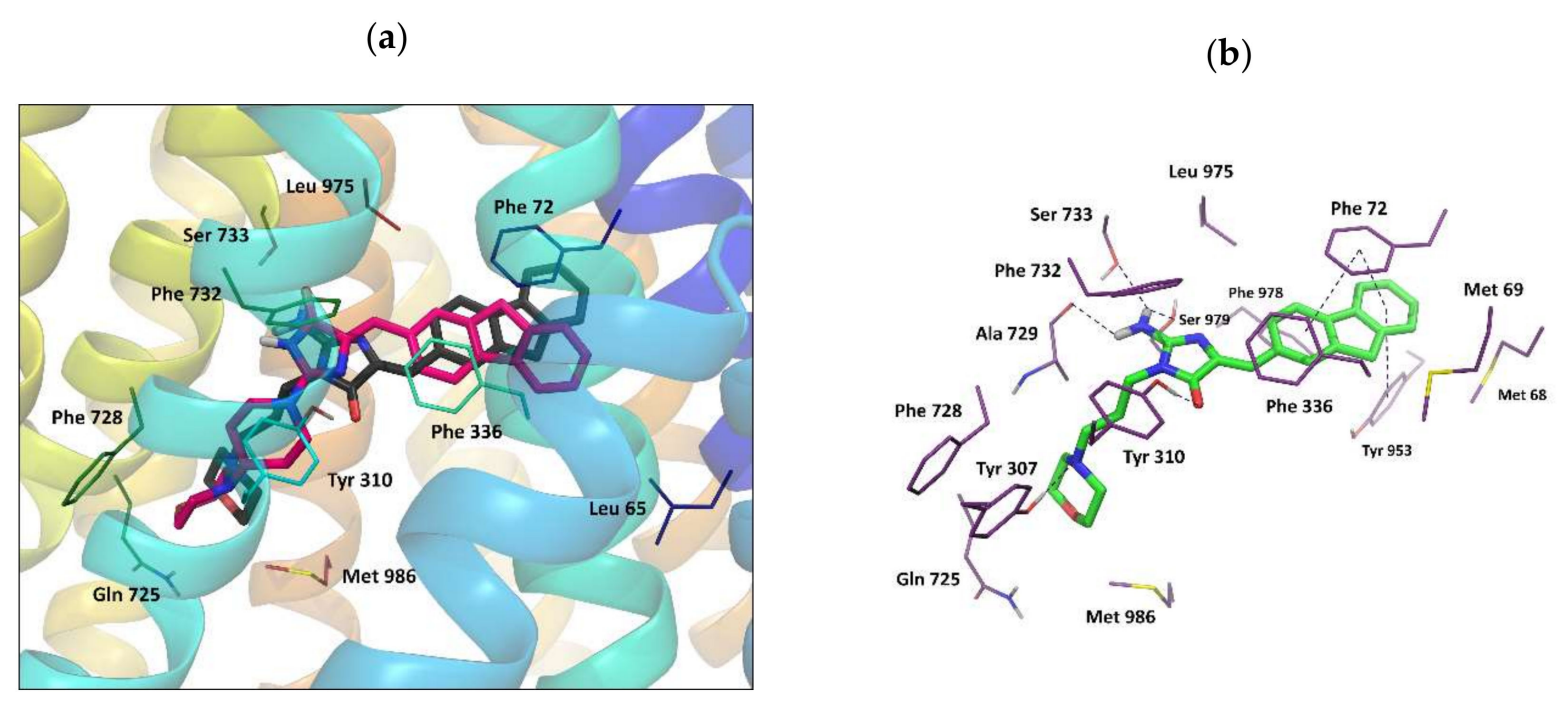
(**a**) Binding mode of compounds **18** and **12** adopting docking pose 1; (**b**) molecular interactions for the best docking pose 1 represented by compound **18**.

**Figure 6:**
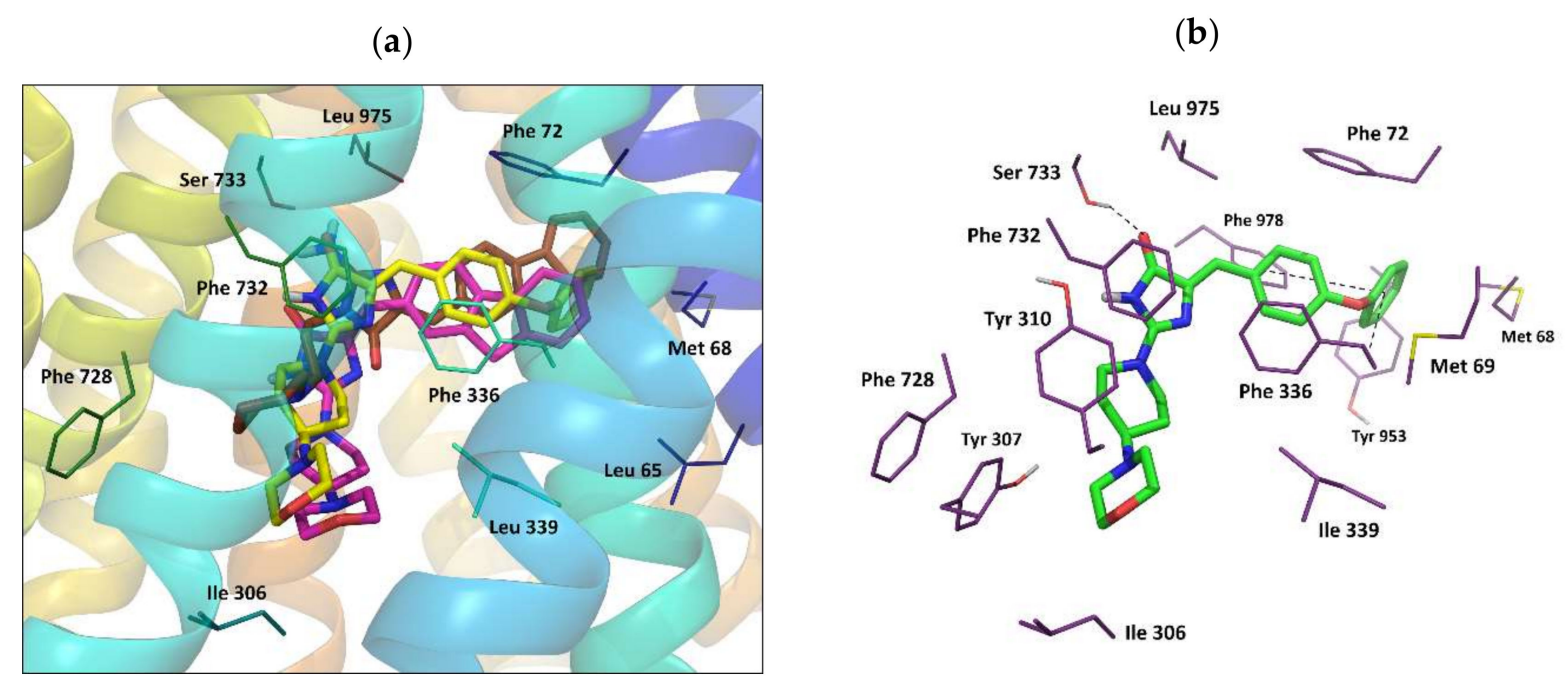
(**a**) Binding mode of compounds **11**, **18**, and **12** adopting docking pose 2; (**b**) molecular interactions for the best docking pose 2 represented by compound **11**.

**Figure 7:**
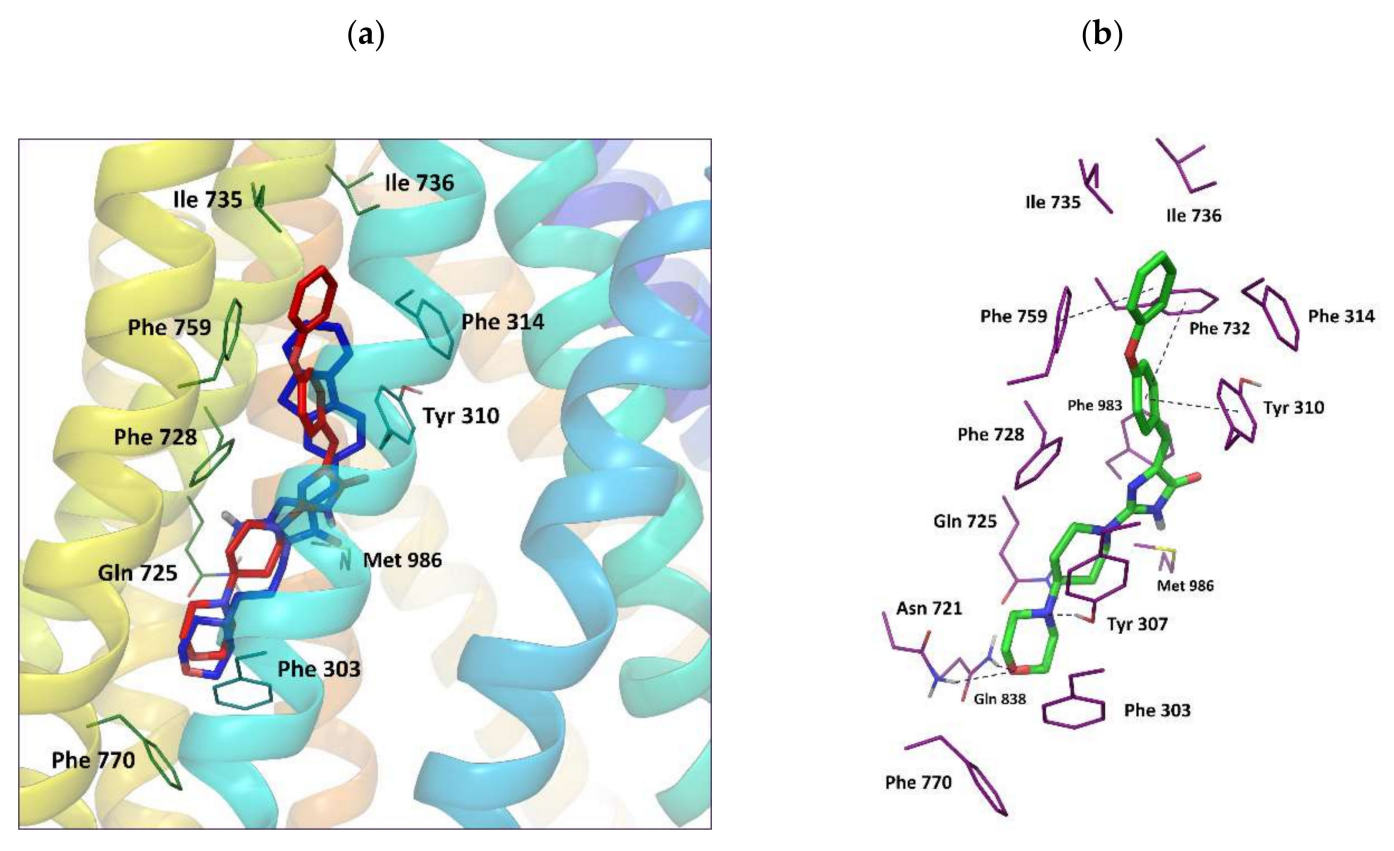
(**a**) Binding mode of compounds **11** and **18** adopting docking pose 3; (**b**) molecular interactions for the best docking pose 3 represented by compound **11**.

Out of docking poses obtained for the neutral forms of compounds, top-ranked poses detected for at least two out of three docked imidazolones were collected in three clusters (Table S2 in the Supplementary). The molecular interactions for each pose are shown for individual compounds with the best docking score (Figure 5, Figure 6 and Figure 7).

The ligand–protein interaction pattern for pose 1 adopted by compound **18** (with the highest docking score and the highest binding energy among all obtained poses, Table S2 in the Supplementary), and by compound **12**, corresponded to the binding mode of verapamil. In this pose **18**, it was found to form H-bonds with Tyr 307 and Tyr 310 in a similar way to verapamil, and additional H-bonds between the amino group of the ligand and Ser 733, Ser 979, and the backbone chain of Ala 729 were observed (Figure 5). The aromatic fluorene moiety of **18** interacted by π–π stacking with Phe 72 and Tyr 953. The whole ligand molecule was involved in numerous hydrophobic contacts with surrounding residues, most of which is known to be a part of the verapamil binding site (Table S2) [31]. Furthermore, according to literature reports, some of the amino acids listed in Table S2 for pose 1 are also a part of the putative binding site for rhodamine dyes - R site (Met 68, Met 69, Phe 72, Phe 336, Phe 732, Tyr 953, Phe 957, Leu 975, Phe 978) [31,33].

Pose 2, found in the case of all three docked imidazolones **11**, **12**, and **18**, was, in fact, very similar to pose 1, with the difference in location of the morpholine tail attached to the imidazolone ring at the 2- or 3-position (Figure 6). This part of the molecule in pose 2 was oriented toward TM5 and TM6 (instead of TM7, like in the case of pose 1) and formed hydrophobic contacts with Ile 306, Tyr 307, Tyr 310, and Leu 339. The aromatic moiety of ligands was involved in π–π stacking interactions with surrounding phenylalanines and tyrosines (Phe 978 and Tyr 953 in the case of **11**). A hydrogen bond with Ser 733 was detected for compounds **11** and **18**, while in the case of **12**, this interaction was replaced by a H-bond with Ser 979.

An interesting pose 3 with high docking scores and high binding energy values (only slightly below those calculated for pose 1 and 2, Table S2, Supplementary) was observed for compounds **11** and **18**. In this case, imidazolone derivatives adopted a more extended conformation, located among TM5, TM7, TM8, and TM12 in almost perfectly parallel orientation to α-helices, with an aromatic “head” directed to the outer leaflet of the lipid bilayer. It was found that compound **11** in this position formed hydrogen bonds with surrounding polar residues: Asn 721, Gln 838, and Tyr 307 (Figure 7), and π–π stacking interactions with Phe 732, Phe 759, and Tyr 310. Additionally, the ligand was involved in hydrophobic contacts with the amino acids that belong mostly to TM5 (Phe 303, Ala 311, Phe 314, Trp 315, Thr 318), TM7 (Leu 724, Gln 725, Phe 728, Ile 735, Ile 736), TM8 (Ser 756, Ser 766, Phe 770), and TM12 (Phe 983, Met 986) (Table S2). The binding mode observed for pose 3 resembled to some extent that proposed by Pajeva for Hoechst 33342, according to which Tyr 307, Asn 721, and Phe 770 were identified as important residues forming a part of the H-site [33].

Summing up, results of the docking studies confirm that imidazolones (**11**, **12**, **18**) are highly probable to interact with the transmembrane interior of Pgp with a mechanism similar to verapamil. Analysis of the docking results showed that the binding pocket for imidazolones most likely involves the residues responsible for binding verapamil and, partially, residues of the putative binding site for rhodamine dyes. These results are in good accordance with those coming from the Pgp ATPase assay. Thus, they support our hypothesis of imidazolone modulator–rhodamine 123 substrate competitive action to the same Pgp-binding site as the most probable mechanism of the dye-efflux inhibition caused by the imidazolones.

### 2.4. Safety In Vitro

**Figure 8:**
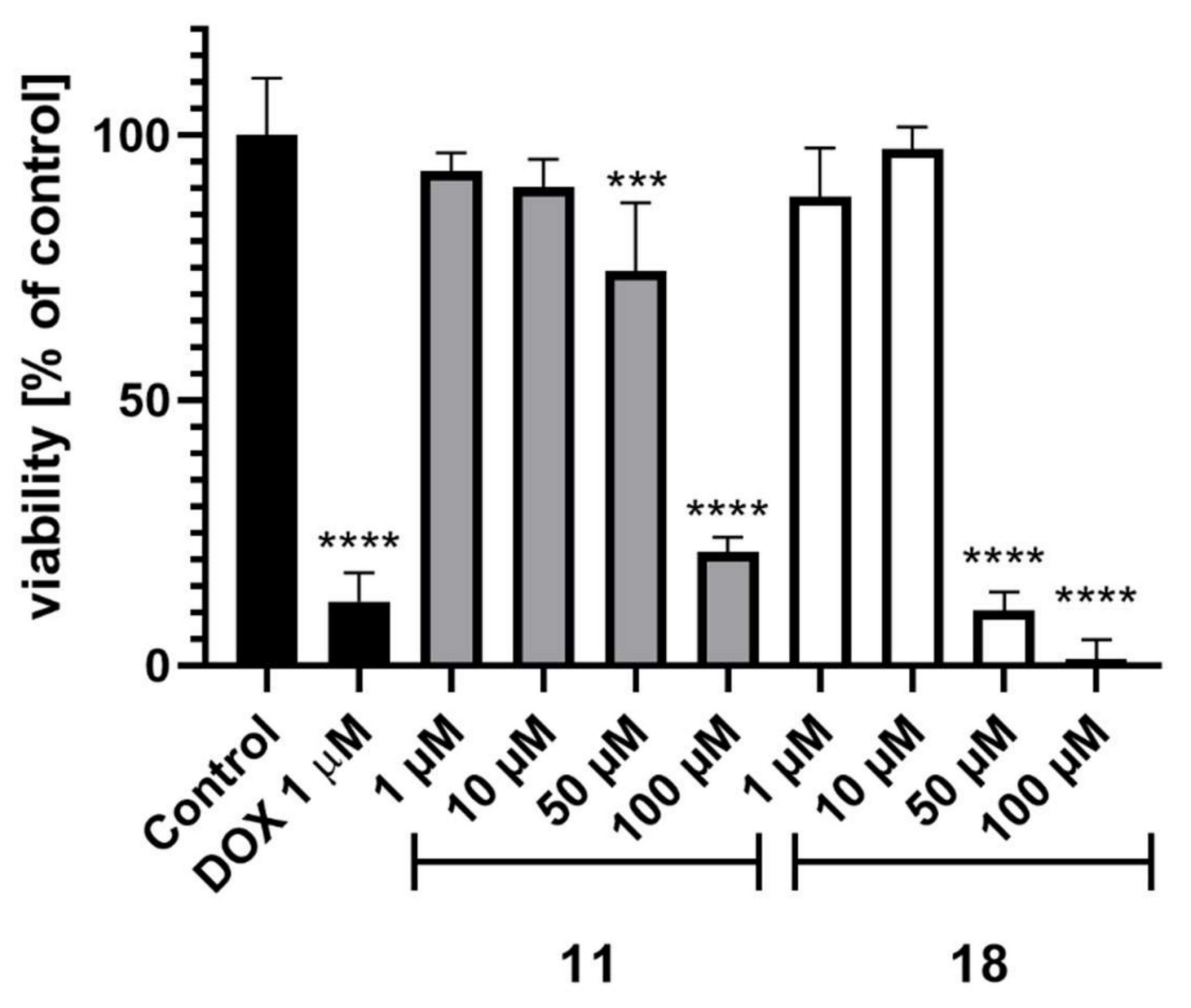
The viability of the HEK-293 cell line after incubation with compound **11** or **18** for 72 h. DOX—doxorubicin. Control—1% DMSO in the cell culture media. The statistical significance was evaluated by a one-way ANOVA, followed by Bonferroni’s comparison test (*** *p* < 0.001, **** *p* < 0.0001 compared to control).

In order to determine the safety of the most active ABCB1 modulators (**11**, **18**), cell-based toxicity assays in vitro were performed using the standard colorimetric MTS procedure in the human embryonic kidney HEK-293 cell line. Results are shown in Figure 8. Both compounds significantly decreased HEK-293 cell viability at the highest used concentrations (50 and 100 µM). Compound **18** was distinctly more toxic than **11** as, at the same concentration (50 µM), **18** decreased the cell viability up to 10.3%, whereas **11** only decreased it to 84.2%. However, the tested imidazolones (**11**, **18**) were found as relatively safe in comparison to the reference doxorubicin (DOX), tested at 1 µM.

## 3. Discussion

This study allowed an original group of modulators to be found for the T-lymphoma ABCB1 efflux pump that may be helpful as potential “adjuvants” in order to improve the efficacy of cancer therapy. The new group of morpholine-containing 5-arylideneimidazolones (**7**–**20**) distinctly differs from typical Pgp-modulator structures that usually contain more than one hydrophobic/aromatic-terminate fragment [43]. In the case of the imidazolones presented, a bulky hydrophobic area is concentrated at only position 5 of the imidazolone, while the opposite ends, either at position 2 (**7**–**13**, group A) or at position 3 (**14**–**20**, group B), demonstrate a significant decrease in hydrophobic properties in favor of alkaline ones, protonable to form HCl salts. Nevertheless, this deviation from the typical Pgp inhibitor structure allowed some compounds to have a high potency to inhibit the dye-substrate efflux, in comparable range with verapamil, or even higher. The mutual structural diversity within the considered series (**7**–**20**), however, seems to be responsible for various kinds of forces of expected biological activity, and, therefore, allows us to postulate some qualitative structure–activity relationship (SAR).

The main structural difference consists of the two ways of substitution topology at positions 2 and 3, i.e., the unsubstituted position 3 (group A) and the presence of a NH_2_ group at position 2 and alkyl-linked morpholine at position 3 (group B). Furthermore, the modifications performed give the possibility to estimate a role for the activity of chemical moieties, as follows: (i) The aromatic area within both groups (A and B), (ii) the morpholine-containing moieties (group A), and finally (iii) the length of alkyl linker at position 3 (group B).

As topology is concerned, the most active dye efflux inhibitors (**11** and **18**) represent both groups (A and B), and there are either inactive or slightly active compounds found in both topological groups (**12**, **13** vs. **15**–**17**, Table 1). Thus, both kinds of topology (A and B) seem to equally contribute in the desirable Pgp modulatory action. This has also been confirmed by our molecular modeling results that indicated energetically favorable and mutually similar binding modes in the verapamil binding site for representative members of both A (**11**, **12**) and B (**18**).

In terms of insight into the role of the three modified moieties, at least three main modes of the biological action should be considered, i.e., (1) the potency to inhibit 123 rhodamine efflux in the accumulation assay, (2) the substrate “force” of compounds, observed as a difference in the cytotoxic action between PAR and MDR T-lymphoma cells, and (3) the substrate properties seen as the ability to increase the intrinsic ATPase activity of Pgp. In this context, the fluorene derivative containing the propyl-linked morpholine moiety (**18**, group B) was found as the best agent, displaying both the dye-inhibition in the accumulation assay and the substrate potency in the Pgp ATPase test, noticeably higher than the reference verapamil. Furthermore, the Pgp-substrate properties of **18** can be confirmed by an almost two-times-higher cytotoxicity toward PAR T-lymphoma cells in comparison to MDR ones.

On the other hand, the *p*-phenoxybenzylidene derivative with the piperidine-morpholine moiety at position 2 (**11**, group A) was even slightly more potent than **18** to inhibit the dye-substrate efflux, while the substrate potency of **11** was significantly weaker in either the Pgp ATPase- or the PAR vs. MDR T-lymphoma cytotoxicity assays. Despite this, the compound was less toxic than **18** for HEK-293 cells and, therefore, could be the most proper among the investigated imidazolones for development in search for new “adjuvants” of anticancer therapy.

Although the two compounds (**11** and **18**) were the most promising therapeutically, this comprehensive study allowed us to also recognize other members that undoubtedly influence the ABCB1-efflux pump and can contribute to SAR analysis. Among them, the anthracene derivative with the propyl-linked morpholine moiety at position 2 (**19**, group B) displayed the strongest substrate properties in the PAR/MDR T-lymphoma cytotoxicity assays (Table 3), while it did not act as an efflux pump inhibitor in the accumulation assay (Table 2). This compound (**19**) differs from **18** only within the aromatic area, i.e., possesses a slightly bigger and “more aromatic”, compared to fluorene, anthracene group. This bulky and stiffened steric hindrance may be responsible for “a defeat” in the competition with rhodamine 123 to be expelled by the ABCB1-pump.

Despite the different effects of imidazolones (**7**–**20**), observed depending on the tests carried out, some structural features, which benefit the dye efflux inhibition, can be captured. In the group with unsubstituted nitrogen at position 3 (**7**–**13**, group A), the piperidine-morpholine substituent at imidazolone position 2 (**8**, **10**–**13**) is distinctly more favorable than either the longer piperazine-ethyl-morpholine (**9**) or the shorter directly substituted morpholine (**7**). The compounds (**7** and **9**) did not show any syndrome of action toward the ABCB1-efflux pump, either as substrates or as inhibitors. The influence of the piperidine-morpholine scaffold is, however, closely associated with the aromatic substituents at the 5-imidazolone. Thus, the flexible *p*-phenoxy-phenyl moiety can be identified as the most profitable (**11**), and compared to the significantly less active *m*-phenoxy-phenyl analog (**10**), it is supposed that not only steric but also electronic properties can be responsible for the considered biological actions, with a preference for the electro-donating substituent at position *para*. In group A, the tricyclic aromatic moieties seem to be profitable, too (**12**, **13**), in particular, the fluorene moiety present in compound **12**, while a slight ABCB1-modulatory effect was also found for the anthracene derivative (**13**). These results are not surprising, as the fluorene moiety is also a beneficial motif of our earlier efflux pump inhibitors, coming from different chemical groups [21,22,25].

The role of the fluorene motif is even more exposed in group B, being a trait of the most active agent (**18**). Apart from the role of aromatic area, the group B with topology resulting from the Dimroth rearrangement allows us to analyze the role of the spacer between the imidazolone and the morpholine fragments. Results of the whole biological screening indicate that the compounds affecting the efflux pump (**15**–**19**) include the three-carbon linker, while the two-carbon analog of **15**, compound **14**, was inactive along the biological studies. Unlike group A, topology B does not seem to discriminate between *para* (**17**) and *meta* (**16**) positions in the case of the phenyloxy-phenyl aromatic area, and even some predominant influence of meta-substitution could be observed in both the accumulation and cytotoxicity assays, respectively (**16** vs. **17**, Table 2 and Table 3).

Summing up, the performed SAR analysis allowed us to recognize chemical moieties that could be beneficial for the action on the cancer ABCB1-efflux pump, i.e., the fluorene, phenoxyphenyl, or anthracene as aromatic moieties of 5-arylideneimidazolone on the one hand, and either the morpholine-piperidine at position 2 or the propyl-linked morpholine at position 3 of the imidazolone core, on the other.

## 4. Materials and Methods

### 4.1. Chemistry

Reagents were purchased from Alfa Aesar (Karlsruhe, Germany) or Sigma Aldrich (Darmstadt, Germany) or Acros Organics (Geel, Belgium). Reaction progress was verified using thin-layer chromatography (TLC), which was carried out on 0.2 mm Merck silica gel 60 F254 plates. Spots were visualized by UV light. Melting points (m.p.) were determined using MEL-TEMP II apparatus (LD Inc., Long Beach, CA, USA) and are uncorrected. ^1^H-NMR and ^13^C-NMR spectra were obtained on a Mercury-VX 300 Mz spectrometer (Varian, Palo Alto, CA, USA) in DMSO-d_6_. Chemical shifts in ^1^H-NMR spectra were reported in parts per million (ppm) on the δ scale using the solvent signal as an internal standard. Data are reported as follows: Chemical shift, multiplicity (s, singlet; br. s, broad singlet; d, doublet; d def.—doublet deformed; t, triplet; t def.—triplet deformed; m, multiplet), coupling constant J in Hertz (Hz), number of protons, proton position (Ar—aromatic moiety at position 5, Pp—piperidine, Pip—piperazine, Mor—morpholine). Mass spectra were recorded on a UPLC-MS/MS system consisting of a Waters ACQUITY^®®^UPLC^®®^ (Waters Corporation, Milford, MA, USA) coupled to a Waters TQD mass spectrometer (electrospray ionization mode ESI-tandem quadrupole). Chromatographic separations were carried out using the Acquity UPLC BEH (bridged ethyl hybrid) C18 column; 2.1 × 100 mm, and 1.7 µm particle size, equipped with an Acquity UPLC BEH C18 VanGuard precolumn (Waters Corporation, Milford, MA, USA); 2.1 × 5 mm, and 1.7 µm particle size. The column was maintained at 40 °C and eluted under gradient conditions from 95% to 0% of eluent A over 10 min, at a flow rate of 0.3 mL·min^−1^. Eluent A: Water/formic acid (0.1%, *v/v*); eluent B: Acetonitrile/formic acid (0.1%, *v*/*v*). Chromatograms were made using a Waters eλ PDA detector. Spectra were analyzed in the 200–700 nm range with a 1.2 nm resolution and sampling rate of 20 points/s. MS detection settings of the Waters TQD mass spectrometer were as follows: Source temperature 150 °C, desolvation temperature 350 °C, desolvation gas flow rate 600 L·h^−1^, cone gas flow 100 L·h^−1^, capillary potential 3.00 kV, cone potential 40 V. Nitrogen was used for both nebulizing and drying gas. The data were obtained in a scan mode ranging from 50 to 1000 m/z in 0.5 s time intervals. The data acquisition software was MassLynx V 4.1 (Waters Corporation, Milford, MA, USA). Retention times (t_R_) are given in minutes. The UPLC/MS purity of all final compounds was determined (%). Synthesis of intermediates **21**–**32** and final products **16**, **19** has been described earlier [21,22,44,45,46].

#### General Procedure for Synthesis of Final Products (**7–15**,**17–18**,**20**)

(Z)-5-arylidene-2-(methylthio)-3H-imidazol-4(5H)-one (2–5 mmol) and primary or secondary amine derivative (2.5–6 mmol) were heated for 15 min without solvent in an oil bath with controlled temperature (120–130 °C). Then, ethanol was added and the mixture was heated for 5–7 h. After that, it was mixed for the next 20 h. Conversion into a hydrochloride form was performed using gaseous hydrochloride acid. When necessary, purification was performed using crystallization from ethanol.

##### (Z)-5-(Biphen-4-ylmethylene)-2-morpholino-3H-imidazol-4(5H)-one hydrochloride (7)

(Z)-5-(Biphen-4-ylmethylene)-2-(methylthio)-3H-imidazol-4(5H)-one (**27**) (4 mmol, 1.18 g) and morpholine (6 mmol, 0.52 g) were used. Yellow solid. Yield 63.24%; mp 248–252 °C. C_20_H_20_ClN_3_O_2_ MW 369.84. LC/MS±: Purity 96.25% t_R_ = 5.98, (ESI) m/z [M+H] 334.15. ^1^H-NMR (DMSO-d_6_, ppm): δ 8.01-7.99 (d def., 2H, Ar-2,6-H), 7.72–7.69 (d def., 5H, Ar-3,5,2`,6`-H, N3-H), 7.49-7.44 (t def., 2H, Ar-3`,5`-H), 7.39–7.34 (m, 1H, Ar-4`-H), 6.60 (s, 1H, C=CH), 3.72–3.70 (m, 8H, Mor). ^13^C NMR (75 MHz, dmso) δ [ppm]: 140.12, 139.15, 131.05, 129.40, 127.92, 126.91, 65.98, 45.33, 40.79, 40.50, 40.22, 39.94, 39.66, 39.38, 39.09.

##### (Z)-5-(Biphen-4-ylmethylene)-2-(4-morpholinopiperidin-1-yl)-imidazol-4(5H)-one hydrochloride (8)

(Z)-5-(Biphen-4-ylmethylene)-2-(methylthio)-3H-imidazol-4(5H)-one (**27**) (4 mmol, 1.18 g) and 4-morpholinopiperidine (6 mmol, 1.02 g) were used. Yellow solid. Yield 59.01%; mp 267–269 °C. C_25_H_29_ClN_4_O_2_ MW 452.98. LC/MS±: Purity 100.00% t_R_ = 4.54, (ESI) m/z [M+H] 417.23. ^1^H-NMR (DMSO-d_6_, ppm): δ 11.71 (s, 1H, NH^+^), 7.98-7.95 (d def., 2H, Ar-2,6-H), 7.75-7.70 (t def., 4H, Ar-3,5,2`,6`-H), 7.49-7.45 (t def., 2H, Ar-3`,5`-H), 7.39-7.35 (m, 1H, Ar-4`-H), 6.69 (s, 1H, CH=C), 4.97–2.74 (m, 13H, Mor, Pp-2,4,6-H), 2.31-2.27 (d def., 2H, Pp-3,5-H_b_), 1.91-1.79 (m, 2H, Pp-3,5-H_a_). ^13^C-NMR (75 MHz, dmso) δ [ppm]: 140.15, 138.90, 130.92, 129.39, 127.88, 126.89, 66.99, 60.96, 49.74, 40.79, 40.51, 40.23, 39.95, 39.67, 39.38, 39.10, 27.85.

##### (Z)-5-(Biphen-4-ylmethylene)-2-(4-(2-morpholinoethyl)piperazin-1-yl)-imidazol-4(5H)-one hydrochloride (9)

(Z)-5-(Biphen-4-ylmethylene)-2-(methylthio)-3H-imidazol-4(5H)-one (**27**) (2 mmol, 0.59 g) and 4-(2-morpholinoethyl)piperazine (2.5 mmol, 0.5 g) were used. Cream solid. Yield 59.81%; mp 305–307 °C. C_26_H_32_ClN_5_O_2_ MW 482.02. LC/MS±: Purity 100.00% t_R_ = 4.60, (ESI) m/z [M+H] 446.27. ^1^H-NMR (DMSO-d_6_, ppm): δ 8.12-8.10 (d def., 2H, Ar-2,6-H), 7.71-7.66 (m, 5H, Ar-3,5,2`,6`-H, N3-H), 7.48-7.43 (t def., 2H, Ar-3`,5`-H), 7.38-7.33 (t def., 1H, Ar-4`-H), 6.46 (s, 1H, C=CH), 3.88 (s, 6H, Mor-2,6-H, N-CH_2_), 3.70-2.60 (m, 1H, Mor-3,5-H, N-CH_2_-CH_2__,_ Pip). ^13^C-NMR (75 MHz, dmso) δ [ppm]: 140.14, 131.03, 129.40, 126.90, 66.65, 56.16, 55.21, 54.10, 52.82, 40.80, 40.51, 40.23, 39.95, 39.67, 39.38, 39.10.

##### (Z)-5-(3-Phenoxybenzylidene)-2-(4-morpholinopiperidin-1-yl)-imidazol-4(5H)-one hydrochloride (10)

(Z)-5-(3-Phenoxybenzylidene)-2-(methylthio)-3H-imidazol-4(5H)-one (**28**) (4 mmol, 1.24 g) and 4-morpholinopiperidine (6 mmol, 1.02 g) were used. Cream solid. Yield 89.26%; mp 289–292 °C. C_25_H_29_ClN_4_O_3_ MW 468.98. LC/MS±: Purity 99.33% t_R_ = 4.60, (ESI) m/z [M+H] 433.18. ^1^H-NMR (DMSO-d_6_, ppm): δ 11.80 (s, 1H, NH^+^), 7.79-7.61 (m, 1H, N3-H), 7.45-7.35 (m, 5H, Ar-2,5-H, Ar-3`,4`,5`-H), 7.23–7.13 (m, 1H, Ar-6-H), 7.12-7.03 (m, 2H, Ar-2`,6`-H), 7.02-6.88 (m, 1H, Ar-4-H), 6.51 (s, 1H, CH=C), 4.21–4.17 (d.def, 2H, Mor-2,6-H_b_), 3.95-3.93 (d.def, 2H, Mor-2,6-H_a_), 3.58-3.22 (m, 4H, Pp-2,6-H), 3.03 (s, 5H, Pp-4-H, Mor-3,5-H), 2.27-2.23 (d def., 2H, Pp-3,5-H_b_), 2.03-1.70 (m, 2H, Pp-3,5-H_a_). ^13^C NMR (75 MHz, dmso) δ [ppm]: 157.98, 156.33, 138.24, 130.43, 130.05, 125.36, 124.28, 120.34, 117.99, 117.30, 110.39, 66.99, 60.90, 49.71, 40.78, 40.50, 40.21, 39.93, 39.65, 39.37, 39.08, 27.78.

##### (Z)-5-(4-Phenoxybenzylidene)-2-(4-morpholinopiperidin-1-yl)-imidazol-4(5H)-one hydrochloride (11)

(Z)-5-(4-Phenoxybenzylidene)-2-(methylthio)-3H-imidazol-4(5H)-one (**29**) (4 mmol, 1.24 g) and 4-morpholinopiperidine (5 mmol, 0.85 g) were used. Yellow solid. Yield 77.80%; mp 249–251 °C. C_25_H_29_ClN_4_O_3_ MW 468.98. LC/MS±: Purity 96.12% t_R_ = 4.45, (ESI) m/z [M+H] 433.25. ^1^H-NMR (DMSO-d_6_, ppm): δ 11.34 (s, 1H, NH^+^), 8.14-7.98 (d def., 2H, Ar-2,6-H), 7.46-7.32 (t def., 2H, Ar-3`,5`-H), 7.19-7.09 (t def., 1H, Ar-4`-H), 7.06-6.91 (m, 4H, Ar-3,5-H, Ar-2`,6`-H), 6.33 (s, 1H, CH=C), 4.31 (s, 2H, Mor-2,6-H_b_), 3.85 (s, 4H, Mor-2,6-H_a_, Pp-2,6-H_b_), 3.55-2.83 (m, 7H, Pp-2,6-H_a_, Pp-4-H, Mor-3,5-H), 2.30–2.03 (m, 2H, Pp-3,5-H_b_), 1.65 (s, 2H, Pp-3,5-H_a_). ^13^C NMR (75 MHz, dmso) δ [ppm]: 156.80, 156.21, 132.13, 131.80, 130.51, 124.06, 119.25, 118.73, 110.88, 66.97, 60.97, 56.46, 49.72, 40.78, 40.49, 40.21, 39.93, 39.65, 39.37, 39.08, 27.86, 19.00.

##### (Z)-5-((9H-Fluoren-2-yl)methylene)-2-(4-morpholinopiperidin-1-yl)-imidazol-4(5H)-one hydrochloride (12)

(Z)-5-((9H-Fluoren-2-yl)methylene)-2-(methylthio)-3H-imidazol-4(5H)-one (**30**) (5 mmol, 1.55 g) and 4-morpholinopiperidine (6 mmol, 1.02 g) were used. Orange solid. Yield 44.39%; mp 274–276 °C. C_26_H_29_ClN_4_O_2_ MW 464.99. LC/MS±: Purity 97.41% t_R_ = 4.65, (ESI) m/z [M+H] 429.19. ^1^H-NMR (DMSO-d_6_, ppm): δ 11.37 (s, 1H, NH^+^), 8.24 (s, 1H, Ar-1-H), 8.12-8.09 (d def., 1H, Ar-4-H), 7.88-7.85 (d def., 3H, Ar-5,8-H, N3-H), 7.57-7.55 (d def., 1H, Ar-3-H), 7.38-7.27 (m, 2H, Ar-6,7-H), 6.42 (s, 1H, CH=C), 4.35 (s, 2H, Ar-CH_2_), 4.07–3.62 (m, 6H, Mor-2,6-H_,_ Pp-2,6-H_b_), 3.62–3.20 (m, 2H, Pp-2,6-H_a_), 3.11–3.03 (m, 5H, Pp-4-H, Mor-3,5-H), 2.22 (s, 2H, Pp-3,5-H_b_), 1.72 (s, 2H, Pp-3,5-H_a_). ^13^C-NMR (75 MHz, dmso) δ [ppm]: 185.01, 143.79, 141.08, 126.93, 125.51, 120.64, 67.15, 60.56, 49.74, 40.79, 40.51, 39.30, 39.09, 38.79, 27.94.

##### (Z)-5-(Anthracen-10-ylmethylene)-2-(4-morpholinopiperidin-1-yl)-imidazol-4(5H)-one hydrochloride (13)

(Z)-5-(Anthracen-10-ylmethylene)-2-(methylthio)-3H-imidazol-4(5H)-one (**31**) (4 mmol, 1.22 g) and 4-morpholinopiperidine (5 mmol, 0.85 g) were used. Yellow solid. Yield 38.48%; mp 303–305 °C. C_27_H_29_ClN_4_O_2_ MW 477.00. LC/MS±: Purity 97.60% t_R_ = 4.22, (ESI) m/z [M+H] 441.22. ^1^H-NMR (DMSO-d_6_, ppm): δ 9.75 (s, 1H, N3-H), 8.63-8.54 (d def., 1H, Ar-9-H), 8.12–8.00 (m, 4H, Ar-1,4,5,8-H), 7.54-7.45 (m, 4H, Ar-2,3,6,7-H), 7.11 (s, 1H, C=CH), 3.93-3.78 (m, 4H, Mor-2,6-H), 3.60-2.70 (m, 9H, Pp-2,4,6-H, Mor-3,5-H), 2.25–2.02 (m, 2H, Pp-3,5-H_b_), 1.58 (s, 2H, Pp-3,5-H_a_).

##### (Z)-5-(Biphen-4-yl)-2-amino-3-(2-morpholinoethyl)-imidazol-4(5H)-one hydrochloride (14)

(Z)-5-(Biphen-4-ylmethylene)-2-(methylthio)-3H-imidazol-4(5H)-one (**27**) (4 mmol, 1.18 g) and 2-morpholinoethan-1-amine (5 mmol, 0.65 g) were used. Yellow solid. Yield 33.10%; mp 266–270 °C. C_22_H_25_ClN_4_O_2_ MW 412.91. LC/MS±: Purity 98.26% t_R_ = 4.60, (ESI) m/z [M+H] 377.22. ^1^H-NMR (DMSO-d_6_, ppm): δ 7.97 (s, 2H, NH_2_-hydantoin), 7.80–7.66 (m, 5H, Ar-2,3,5,6-H, Ar-2`-H), 7.54-7.44 (t def., 3H, Ar-3`,5`,6`-H), 7.41-7.32 (m, 1H, Ar-4`-H), 6.60 (s, 1H, CH=C), 4.26-2.97 (m, 12H, Mor-2,3,5,6-H, N3-CH_2_-CH_2_). ^13^CNMR (75 MHz, dmso) δ [ppm]: 140.12, 130.99, 130.89, 129.39, 127.88, 126.92, 66.64, 53.66, 40.79, 40.51, 40.22, 39.94, 39.66, 39.38, 39.09.

##### (Z)-5-(Biphen-4-ylmethylene)-2-amino-3-(3-morpholinopropyl)-imidazol-4(5H)-one hydrochloride (15)

(Z)-5-(Biphen-4-ylmethylene)-2-(methylthio)-3H-imidazol-4(5H)-one (**27**) (4 mmol, 1.18 g) and 3-morpholinopropan-1-amine (6 mmol, 0.87 g) were used. Yellow solid. Yield 56.93%; mp 248–252 °C. C_23_H_27_ClN_4_O_2_ MW 426.94. LC/MS±: Purity 97.42% t_R_ = 4.39, (ESI) m/z [M+H] 391.17. ^1^H-NMR (DMSO-d_6_, ppm): δ 7.86 (s, 2H, NH_2_-hydantoin) 7.89-7.65 (t def., 5H, Ar-2,3,5,6-H, Ar-2`-H), 7.52-7.45 (t def., 3H, Ar-3`,5`,6`-H), 7.40-7.33 (t def., 1H, Ar-4`-H), 6.77 (br. s, 1H, CH=C), 3.98-2.80 (m, 12H, Mor, N3-CH_2_-CH_2_-CH_2_), 2.13-2.00 (m, 2H, N3-CH_2_-CH_2_).

##### (Z)-5-(4-Phenoxybenzylidene)-2-amino-3-(3-morpholinopropyl)-imidazol-4(5H)-one hydrochloride (17)

(Z)-5-(4-Phenoxybenzylidene)-2-(methylthio)-3H-imidazol-4(5H)-one (**29**) (4 mmol, 1.24 g) and 3-morpholinopropan-1-amine (6 mmol, 0.87 g) were used. Cream solid. Yield 70.62%; mp 210–212 °C. C_23_H_27_ClN_4_O_3_ MW 442.94. LC/MS±: Purity 100.00% t_R_ = 4.32, (ESI) m/z [M+H] 407.26. ^1^H-NMR (DMSO-d_6_, ppm): δ 11.36 (s, 1H, NH^+^), 9.37 (s, 1H, NH), 7.79 (br. s, 2H, NH_2_-hydantoin), 7.47-7.37 (t def., 3H, Ar-2,6-H, Ar-3`-H), 7.25-7.14 (t def., 1H, Ar-5`-H), 7.11-6.97 (m, 5H, Ar-3,5-H, Ar-2`,4`,6`-H), 6.76 (br. s, 1H, CH=C), 3.88 (s, 4H, Mor-2,6-H), 3.67-2.87 (m, 8H, Mor-3,5-H, N3-CH_2_-CH_2_-CH_2_), 2.05 (s, 2H, N3-CH_2_-CH_2_).^13^C NMR (75 MHz, dmso) δ [ppm]: 156.84, 156.10, 132.09, 130.50, 124.03, 119.17, 118.74, 66.57, 53.71, 40.78, 40.49, 40.21, 39.93, 39.65, 39.36, 39.08.

##### (Z)-5-((9H-Fluoren-2-yl)methylene)-2-amino-3-(3-morpholinopropyl)-imidazol-4(5H)-one hydrochloride (18)

(Z)-5-((9H-Fluoren-2-yl)methylene)-2-(methylthio)-3H-imidazol-4(5H)-one (**30**) (5 mmol, 1.55 g) and 3-morpholinopropan-1-amine (6 mmol, 0.87 g) were used. Orange solid. Yield 63.90%; mp 261–263 °C. C_24_H_27_ClN_4_O_2_ MW 438.95. LC/MS±: Purity 100.00% t_R_ = 4.46, (ESI) m/z [M+H] 403.20. ^1^H-NMR (DMSO-d_6_, ppm): δ 11.30 (br. s, 1H, NH^+^), 9.41 (s, 1H, NH), 8.18–7.91 (m, 5H, NH_2_-hydantoin, Ar-1,4,5-H), 7.90–7.70 (m, 1H, Ar-8-H), 7.69-7.56 (m, 1H, Ar-3-H), 7.53–7.29 (m, 2H, Ar-6,7-H), 6.85 (s, 1H, CH=C), 4.06–2.53 (m, 14H, Ar-9-H, Mor, N3-CH_2_-CH_2_-CH_2_), 2.07 (s, 2H, N3-CH_2_-CH_2_). ^13^C-NMR (75 MHz, dmso) δ [ppm]: 143.85, 143.49, 141.36, 127.25, 125.58, 120.50, 66.59, 53.74, 40.78, 40.50, 40.22, 39.94, 39.66, 39.37, 39.09, 36.76.

##### (Z)-5-(Phenanthren-9-ylmethylene)-2-amino-3-(3-morpholinopropyl)-imidazol-4(5H)-one hydrochloride (20)

(Z)-5-(Phenanthren-9-ylmethylene)-2-(methylthio)-3H-imidazol-4(5H)-one (**32**) (4 mmol, 1.22 g) and 3-morpholinopropan-1-amine (5 mmol, 0.72 g) were used. Yellow solid. Yield 25.62%; mp 238–240 °C. C_25_H_27_ClN_4_O_2_ MW 450.96. LC/MS±: Purity 95.34% t_R_ = 4.40, (ESI) m/z [M+H] 415.23. ^1^H-NMR (DMSO-d_6_, ppm): δ 9.52 (s, 1H, NH_2_-hydantoin), 8.96-8.79 (m, 2H, Ar-4,5-H), 8.39–8.00 (m, 3H, Ar-1,8,10-H), 7.84-7.62 (m, 4H, Ar-2,3,6,7-H), 7.42 (s, 1H, CH=C), 3.89 (s, 4H, Mor-2,6-H), 3.67–2.89 (m, 8H, Mor-3,5-H, N3-CH_2_-CH_2_-CH_2_), 2.19–1.96 (m, 2H, N3-CH_2_-CH_2_). ^13^C NMR (75 MHz, dmso) δ [ppm]: 131.69, 130.38, 130.33, 129.68, 129.16, 127.61, 127.53, 127.40, 123.94, 123.25, 66.58, 53.75, 40.78, 40.49, 40.21, 39.93, 39.65, 39.37, 39.08.

### 4.2. Biological Assays in Mouse T-Lymphoma Cells

#### 4.2.1. Cell Lines

L5178 mouse T-cell lymphoma cells (PAR) (ECACC Cat. No. 87111908, obtained from FDA, Silver Spring, MD, USA) were transfected with pHa MDR1/A retrovirus, as previously described by Cornwell et al. [47]. The ABCB1-expressing cell line L5178Y (MDR) was selected by culturing the infected cells with colchicine. The parental L5178 mouse T-cell lymphoma cells and the L5178Y human *ABCB1*-transfected subline were cultured in McCoy’s 5A medium (Sigma-Aldrich, St Louis, MO, USA) supplemented with 10% heat-inactivated horse serum (Sigma-Aldrich, St Louis, MO, USA), 200 mM L-glutamine (Sigma-Aldrich, St Louis, MO, USA), and a penicillin-streptomycin (Sigma-Aldrich, St Louis, MO, USA) mixture in concentrations of 100 U/L and 10 mg/L, respectively. The cell lines were incubated at 37 °C, in a 5% CO_2_, 95% air atmosphere.

#### 4.2.2. Evaluation of Rhodamine 123 Retention by Flow Cytometry

The potency of the tested compounds as inhibitors of the ABCB1 efflux pump was determined using a fluorescence-based detection system, described previously in the literature [23,26]. Verapamil was applied as a reference inhibitor of the ABCB1 transporter. The parental (PAR) and multidrug-resistant (MDR) mouse T-lymphoma cells were adjusted to a density of 2 × 10^6^/mL, re-suspended in serum-free McCoy’s 5A medium, and distributed in 0.5 mL aliquots into Eppendorf centrifuge tubes. The tested compounds (1 and 10 μL, from a stock solution of 1 mM) were added at different concentrations (2 and 20 μM final concentrations, respectively), and the samples were incubated for 10 min at room temperature. Subsequently, 10 μL (with a final concentration of 5.2 μM) of rhodamine 123 was added to the samples and the cells were incubated for 20 min at 37 °C, washed twice, and re-suspended in 0.5 mL phosphate-buffered saline (PBS) for analysis. The fluorescence intensity of the cell population was measured with a Partec CyFlow flow cytometer (Partec, Munster, Germany). Verapamil was used as a positive control at 20 μM final concentration in the rhodamine 123 exclusion experiments. The mean fluorescence intensity (%) was calculated for the treated MDR and PAR mouse T-lymphoma cells as compared to the untreated cells. The fluorescence activity ratio (FAR) was calculated based on the following equation, which relates the measured fluorescence values:

$$FAR=\frac{MDR_{treated}/MDR_{control}}{parental_{treated}/parental_{control}}\;;Quotient=100\times (FAR_{compound}/FAR_{verapamil})$$

#### 4.2.3. Cytotoxicity and Antiproliferative Effect

Cytotoxicity and antiproliferative assays were performed following the procedure described in the literature [23,48,49]. Parental and multidrug-resistant mouse T-lymphoma cells were used to determine the effect of the arylideneimidazolones on the growth of cells. The effects of increasing concentrations of arylideneimidazolones on cell growth were tested in 96-well flat-bottomed microtiter plates. The compounds were diluted in a volume of 100 μL of medium.

In the case of the mouse T-lymphoma cells, the two-fold serial dilutions were prepared in 100 μL of McCoy’s 5A, horizontally. The parental (PAR) and multidrug-resistant (MDR) mouse T-lymphoma cells were adjusted to a density of 1 × 10^4^ cells (for cytotoxic assay) and 6 × 10^3^ (for antiproliferative assay) in 100 μL of McCoy’s 5A medium and were added to each well, with the exception of the medium control wells.

The culture plates were incubated at 37 °C for 24 h (for cytotoxicity assay) and 72 h (for antiproliferative assay); at the end of the incubation period, 20 μL of MTT (thiazolyl blue tetrazolium bromide, Sigma) solution (from a stock solution of 5 mg/mL) were added to each well. After incubation at 37 °C for 4 h, 100 μL of sodium dodecyl sulfate (SDS) (Sigma) solution (10% in 0.01 M HCI) were added to each well and the plates were further incubated at 37 °C overnight. Cell growth was determined by measuring the optical density (OD) at 540/630 nm with a Multiscan EX ELISA reader (Thermo Labsystems, Cheshire, WA, USA). Inhibition of the cell growth was determined according to the formula below:

$$IC_{50}=100-\left[\frac{OD_{sample}-OD_{medium\;control}}{OD_{cell\;control}-OD_{medium\;control}}\right]\times 100$$

Results have been expressed in terms of IC_50_, defined as the inhibitory dose that reduces the growth of the cells exposed to the tested compounds by 50%, each representing the mean of a minimum of three independent experiments.

### 4.3. Intrinsic Activity Toward Pgp In Vitro

The luminescent Pgp-Glo™ Assay System used for determination of the arylideneimidazolone influence on P-glycoprotein activity was purchased from Promega (Madison, WI, USA). The assay was performed in triplicate as described previously [27,28,29]. Compounds **11**, **12**, and **18** (100 μM) were incubated with Pgp membranes for 40 min at 37 °C. The references, Pgp-stimulator verapamil (VER) and Pgp-negative compound caffeine (CFN), were incubated at 200 and 100 µM, respectively. For basal P-gp activity calculation, the membranes were incubated with 100 µM of Na_3_VO_4_. The luminescence signal was measured by a microplate reader EnSpire PerkinElmer (Waltham, MA, USA). The statistical significances were calculated using GraphPad Prism 8.0.1 software.

### 4.4. Molecular Modeling

#### 4.4.1. Homology Modeling

The homology model of human P-glycoprotein was constructed by multiple comparative modeling methods. The X-ray structures of murine Pgp: 4Q9H, 4XWK, and 4M1M, due to 87% sequence identity and high diffraction resolution (3.4, 3.5, and 3.8 Å, respectively) were chosen as templates. The sequence of human Pgp multidrug transporter (accession no. P08183) was retrieved from the UniProtKB/TrEMBL database [42]. The multiple sequence alignment was performed by a PROMALS server [50] and the result in the suitable format was used to build the homology model using MODELLER 9.21 software [51]. Out of 1000 models generated, three models with the lowest DOPE (Discrete Optimized Protein Energy) scores were selected for further investigations and refined by minimization of energy in the Schrodinger Suite software [52]. Analysis of the Ramachandran plots for all three models allowed us to choose the most beneficial homology model showing the highest number of residues in the most favored regions (1077; 93.7%) and the lowest number of residues in disallowed regions (4; 0.3%), with a DOPE score −153,884.80 (Figure S1a, Supplementary). This model was subjected to further structure validation using PROCHECK [53], ProSA [54], and QMEAN [55,56] software (Figures S2 and S3, Supplementary).

The topology and the secondary structure of the linker region (amino acids 630–698) were separately predicted using the HMMTOP transmembrane topology prediction server [57]. The additional helix was detected for residues Asp679–Asp689 of the linker. The modelled chain was incorporated into the selected and validated homology model derived by MOLDELLER. The Ramachandran plot computed by the PROCHECK program for the modified structure showed: 94.2% (1083) of residues in the most favored regions, 4.8% (55) of residues in the additional allowed regions, 0.8% (9) of residues in the generously allowed regions, and 0.3% (3) residues in the disallowed regions. Based on these results, the model was considered to meet the requirements of quality and reliability and selected for molecular docking studies.

#### 4.4.2. Induced Fit Docking of Verapamil and Synthesized Compounds

Verapamil was used as a reference compound for analysis of interactions between the investigated compounds and the human Pgp. The possible protonation and tautomeric states, as well as all stereoisomers for the verapamil structure, were generated with the LigPrep module and optimized by conformational search using the options of the MacroModel module implemented in the Schrodinger Suite (Monte Carlo multiple minimization method, OPLS2005 force field, minimization with Truncated Newton Conjugate Gradient option until the root-mean-square of conjugate-gradient was below 0.05 kJ⋅mol^−1^⋅Å^−1^) [58]. Compounds **11**, **12**, and **18** were subjected to the same procedure of ligands preparation as verapamil. Both positively charged and neutral forms of the ligands were used in further calculations.

The protein was prepared for docking in Protein Preparation Wizard (addition of hydrogen atoms, assignment of protonation states of residues, and optimization of hydrogen bond network, minimization of the protein energy using OPLS2005 force field) [52]. Flexible receptor docking was performed using the induced fit protocol as implemented in the Schrodinger Suite [59].

For verapamil, the receptor grid was assigned as a box with the size of 25 Å, built around a centroid of residues of Pgp known from mutagenesis experiments to interact with verapamil, described by Loo at al. [36,37,38]. For the investigated arylideneimidazolone derivatives **11**, **12**, and **18**, the receptor grid box was set to include the whole binding cavity formed by the transmembrane region of the human Pgp, as defined for the P08183 sequence in the UniProt database [42] (residues of TM1: 45–71, TM2: 113–132, TM3: 183–207, TM4: 209–228, TM5: 288–310, TM6: 327–348, TM7: 708–729, TM8: 751–775, TM9: 825–847, TM10: 852–874, TM11: 931–953, TM12: 970–991).

In the first step, Glide docking of ligands was conducted with van der Waals radii scaling by a factor of 0.5. The side chains of residues within 6 Å of ligand poses were predicted and optimized by Prime. Initially docked ligands were re-docked into the resulting ligand–protein complexes with Glide XP precision. The final ligand–protein complexes were ranked and analyzed according to the obtained docking score, XP Glide score (XP GScore), and induced fit docking score (IFD Score) values (Tables S1 and S2, Supplementary).

For the graphic presentation of the selected structures with the highest docking scores, representing individual clusters of poses, PyMOL software [60] was used.

### 4.5. Safety In Vitro

The human embryonic kidney HEK-293 (ATCC CRL-1573) cells were cultured in Dulbecco’s modified Eagle’s medium (DMEM) with 10% fetal bovine serum (FBS) (Gibco, Carlsbad, CA, USA) according to previously described protocols [27,28,29]. For safety assay, cells were seeded in 96-well plates at a concentration of 1 × 10^4^ cells/well and incubated for 24 h at 37 °C in 5% CO_2_ atmosphere to reach 50% of confluence. Compounds **11**, **12**, and **18** were diluted into fresh growth medium and added to the cells at the final concentrations of 0.1–100 μM. The positive control DOX (1 μM) was also added and the cells were incubated for 72 h. The MTS (3-(4,5-dimethylthiazol-2-yl)-5-(3-carboxymethoxyphenyl)-2-(4-sulfophenyl)-2H-tetrazolium) reagent (CellTiter 96^®^ AQueous One Solution Cell Proliferation Assay, Promega, Madison, WI, USA) was added next to each well and incubated for 4 h. The absorbance was measured using a microplate reader (EnSpire, PerkinElmer, Waltham, MA USA) at 490 nm to determine the cells’ viability. The statistical significances were calculated using GraphPad Prism 8.0.1 software.

## 5. Conclusions

In this study, a new series of fourteen 2-amine-5-arylideneimidazolones (**7**–**20**), representing two different topologies (A and B), as potential inhibitors of the main cancer MDR protein, i.e., ABCB1 efflux pump, was investigated. The compounds were designed and synthesized within a 3–4 step synthesis, involving Knoevenagel condensation, S-methylation, and reaction with amines proceeding via Dimroth rearrangement in the case of primary amines. The comprehensive biological screening in vitro, including: the rhodamine 123 accumulation-, cytotoxicity-, and proliferation assays in T-lymphoma cells with either a parental or multidrug resistance level of ABCB1, has indicated that most of the series affected the efflux pump, either as efflux inhibitors and/or as substrates. The structure–activity relationship analysis has shown the fluorene-, phenoxy-phenyl-, or anthracenyl aromatic moieties, as well as the morpholine-piperidine or the propyl-linked morpholine, with respect to the topology, as the benefit to affect this MDR efflux pump. The studies on potential mechanisms of action for the most active modulators (**11**, **12**, and **18**), using the luminescence Pgp-Glo™ Assay in vitro and computer-aided docking to human Pgp protein, have confirmed the Pgp substrate properties, and the competitive action with the rhodamine 123 to the binding site for verapamil within the efflux pump, as the most probable mechanism of the dye efflux inhibition.

This study allowed us to find two of the most active modulators, i.e., (Z)-5-(4-phenoxybenzylidene)-2-(4-morpholinopiperidin-1-yl)-imidazol-4(5H)-one (**11**) and (Z)-5-((9H-fluoren-2-yl)methylene)-2-amino-3-(3-morpholinopropyl)-imidazol-4(5H)-one (**18**), that have shown a significantly stronger efflux inhibitory action than verapamil. Both compounds were less toxic than doxorubicin toward human cells, but **11** was the safer one. Thus, phenoxybenzylidene derivative **11** seems to be the best candidate for extended studies in search for “adjuvants” able to improve the efficacy of cancer therapy, while further chemical modifications in order to improve the safety of **18** are required.

## Figures and Tables

**Table 1:** Structures of tested 5-arylideneimidazolones (**7**–**20**).

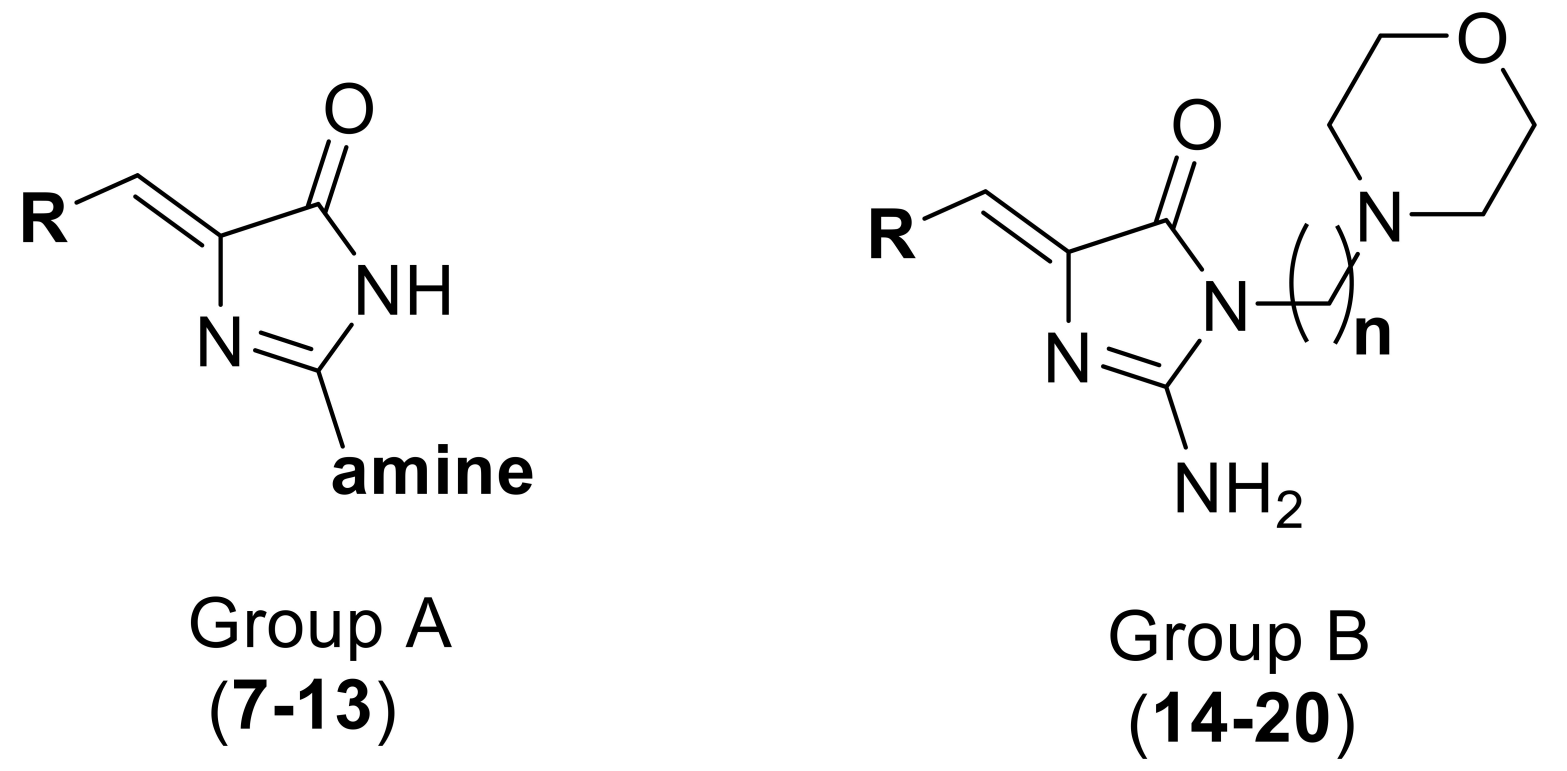				
Cpd	Group	R	n	Amine
**7**	A	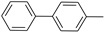	---	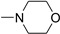
**8**	A	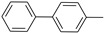	---	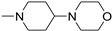
**9**	A	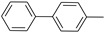	---	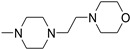
**10**	A	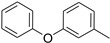	---	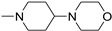
**11**	A	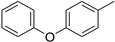	---	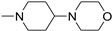
**12**	A	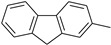	---	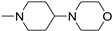
**13**	A	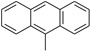	---	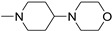
**14**	B	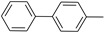	2	---
**15**	B	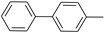	3	---
**16**	B	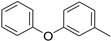	3	---
**17**	B	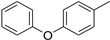	3	---
**18**	B	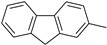	3	---
**19**	B	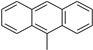	3	---
**20**	B	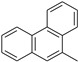	3	---

**Table 2:** Effects of 5-arylideneimidazolones (**7**–**20**) on rhodamine 123 accumulation by multidrug-resistant (MDR) mouse T-lymphoma cells.

Effects on Rhodamine 123 Accumulation				
	Concentration 2 µM		Concentration 20 µM	
Cpd	FAR	FAR Quotient (%)	FAR	FAR Quotient (%)
**7 ^1^**	0.955	7.31	0.927	7.09
**8 ^2^**	1.359	8.49	2.409	15.04
**9 ^1^**	0.766	5.86	1.645	12.58
**10 ^2^**	1.108	6.92	1.992	12.44
**11 ^2^**	1.203	7.51	23.419	146.22
**12 ^1^**	1.161	8.88	6.045	46.23
**13 ^2^**	0.968	6.04	2.073	12.94
**14 ^1^**	0.814	6.22	0.847	6.48
**15 ^1^**	0.887	6.78	2.221	16.98
**16 ^1^**	0.927	7.09	2.426	18.55
**17 ^2^**	0.929	5.80	2.301	14.37
**18 ^1^**	1.038	7.94	18.095	138.37
**19 ^2^**	1.272	7.94	1.615	10.08
**20 ^2^**	0.896	5.59	1.237	7.72
**VER**	-	-	13.077 ^1^/16.016 ^2^	100.00
**DMSO**	0.672 ^1^/0.664 ^2^ (V/V%)	5.14^1^/4.15 ^2^	-	-

VER, verapamil; FAR, fluorescence activity ratio; FAR quotient: FAR of a compound related to FAR of VER tested at 20 µM; ^1,2^ Compounds tested in two groups with respect to different FAR values for VER and DMSO. The highest quotients underlined.

**Table 3:** Cytotoxic effects of 2-amine-5-arylideneimidazolones (**7**–**20**) on cancer cells.

Cpd	Mouse T-Lymphoma Cells				
	PAR		MDR		
	IC_50_ (µM)	SD ±	IC_50_ (µM)	SD ±	SI
**7**	53.52	2.37	54.17	1.42	0.99
**8**	23.97	2.09	46.42	3.37	0.52
**9**	12.82	1.35	18.11	2.53	0.71
**10**	53.96	3.80	63.76	0.30	0.85
**11**	39.06	2.36	45.50	0.60	0.86
**12**	15.82	1.15	30.80	1.56	0.51
**13**	82.22	3.08	>100	-	≤0.82
**14**	36.85	1.12	57.37	5.74	0.64
**15**	17.48	0.53	20.76	0.91	0.84
**16**	16.00	0.98	31.29	2.11	0.51
**17**	21.49	0.45	23.41	1.21	0.92
**18**	15.96	0.68	27.75	1.29	0.58
**19**	28.59	0.60	>100	-	≤0.29
**20**	19.60	2.35	24.88	0.10	0.79
**DOX**	0.7	0.56	2.14	0.76	0.33
**DMSO**	>1%	-	>1%	-	-

PAR, parental T-lymphoma cells; MDR, multidrug-resistant T-lymphoma cells overproducing efflux pump Pgp; DOX, doxorubicin; DMSO, dimethyl sulfoxide; SD, standard deviation; SI, selectivity index; starting concentration of tested compounds: 100 µM.

**Table 4:** Antiproliferative effects of 2-amine-5-arylideneimidazolones (**7**–**20**) on cancer cells.

Cpd	Mouse T-Lymphoma Cells				
	PAR		MDR		
	IC_50_ (µM)	SD ±	IC_50_ (µM)	SD ±	SI
**7**	17.60	0.87	26.33	0.32	0.67
**8**	14.77	1.41	25.98	1.17	0.57
**9**	7.78	0.31	16.53	1.10	0.47
**10**	26.42	1.43	22.45	0.94	1.18
**11**	24.77	0.89	25.60	0.26	0.97
**12**	12.70	0.08	16.20	0.30	0.78
**13**	17.37	0.17	48.58	0.21	0.36
**14**	24.93	0.77	27.86	0.95	0.89
**15**	10.26	0.32	13.64	0.22	0.75
**16**	11.39	0.38	12.16	0.04	0.94
**17**	12.17	1.07	14.25	0.58	0.85
**18**	13.38	0.20	10.82	0.61	1.24
**19**	14.39	0.90	43.82	4.29	0.33
**20**	14.78	0.41	17.31	0.70	0.85
**DOX**	0.28	0.06	1.75	0.38	0.16
**DMSO**	>1%	-	>1%	-	-

PAR, parental T-lymphoma cells; MDR, multidrug-resistant T-lymphoma cells overproducing efflux pump Pgp; SD, standard deviation; SI, selectivity index; starting concentration of tested compounds: 100 µM.

## Supplementary Materials

The following are available online at https://www.mdpi.com/1420-3049/25/9/2258/s1. Spectral data for compounds (^1^HNMRs and ^13^CNMRs), Figure S1: (a) Ramachandran plot for the most beneficial B008 homology model (DOPE –153,884.80); (b) Ramachandran plot for model B008 with a linker incorporated, Figure S2: Evaluation of the top-ranked *h*Pgp homology model B008 by ProSA server, Figure S3: Evaluation of the top-ranked *h*Pgp homology model B008 by QMEAN Swiss Model server, Figure S4: Comparison of the induced fit docking results for the reference pose of verapamil in the neutral form and in the protonated form (top view). The neutral form of verapamil and surrounding residues (side chains only) is marked in pink; the protonated form of the drug and surrounding residues is marked in green. H-bonds between the protein and the ligands are marked by the dashed line, Table S1: Comparison of docking results for top-ranked poses of the neutral and protonated form of verapamil, Table S2: Comparison of the induced fit docking results for top-ranked poses of the imidazolone derivatives **11**, **12**, and **18** (in the neutral form).
