# Supplementary material for: Search for ABCB1 Modulators Among 2-Amine-5-Arylideneimidazolones as a New Perspective to Overcome Cancer Multidrug Resistance
Source: Molecules. 2020 May 11;25(9):2258. doi: 10.3390/molecules25092258 (PMC7249047; doi:10.3390/molecules25092258)
Supplement: Supplementary file 1 [file molecules-25-02258-s001.pdf]

# Computer-aided search for ABCB1 modulators among 2-amine-5-arylideneimidazolones as a new perspective to overcome cancer multidrug resistance

Aneta Kaczor[1], Márta Nové, Annamária Kincses, Gabriella Spengler[2],

Ewa Szymańska[1], Gniewomir Latacz[1], Jadwiga Handzlik[1*]

## Supplementary

**Spectral data for compounds - $^{1}H$ NMR** ............................................. 1

**Spectral data for compounds - $^{13}C$ NMR** ............................................. 13

**Homology modeling** ........................................................................ 23

**Docking results** ............................................................................ 27

**References** ................................................................................. 29

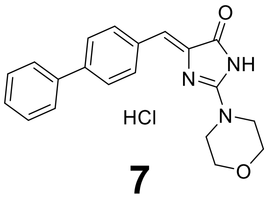

O
NH
N
N
O
HCl


**7**

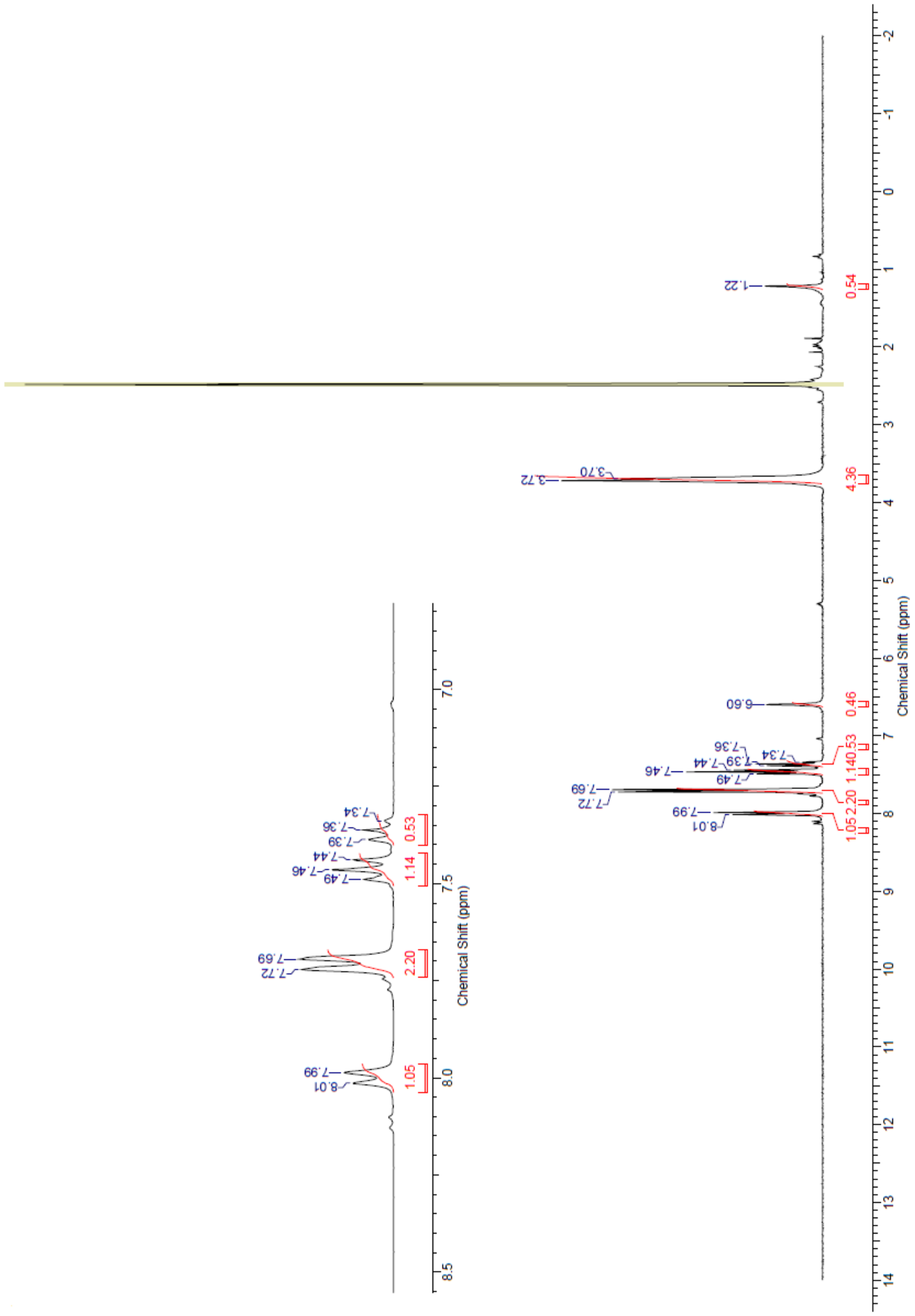

Chemical Shift (ppm)
1.22
3.70
3.72
6.60
7.34
7.36
7.39
7.44
7.46
7.49
7.69
7.72
7.99
8.01
0.54
4.36
0.46
0.53
1.14
2.20
1.05

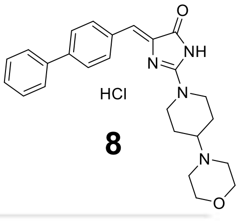


**8**

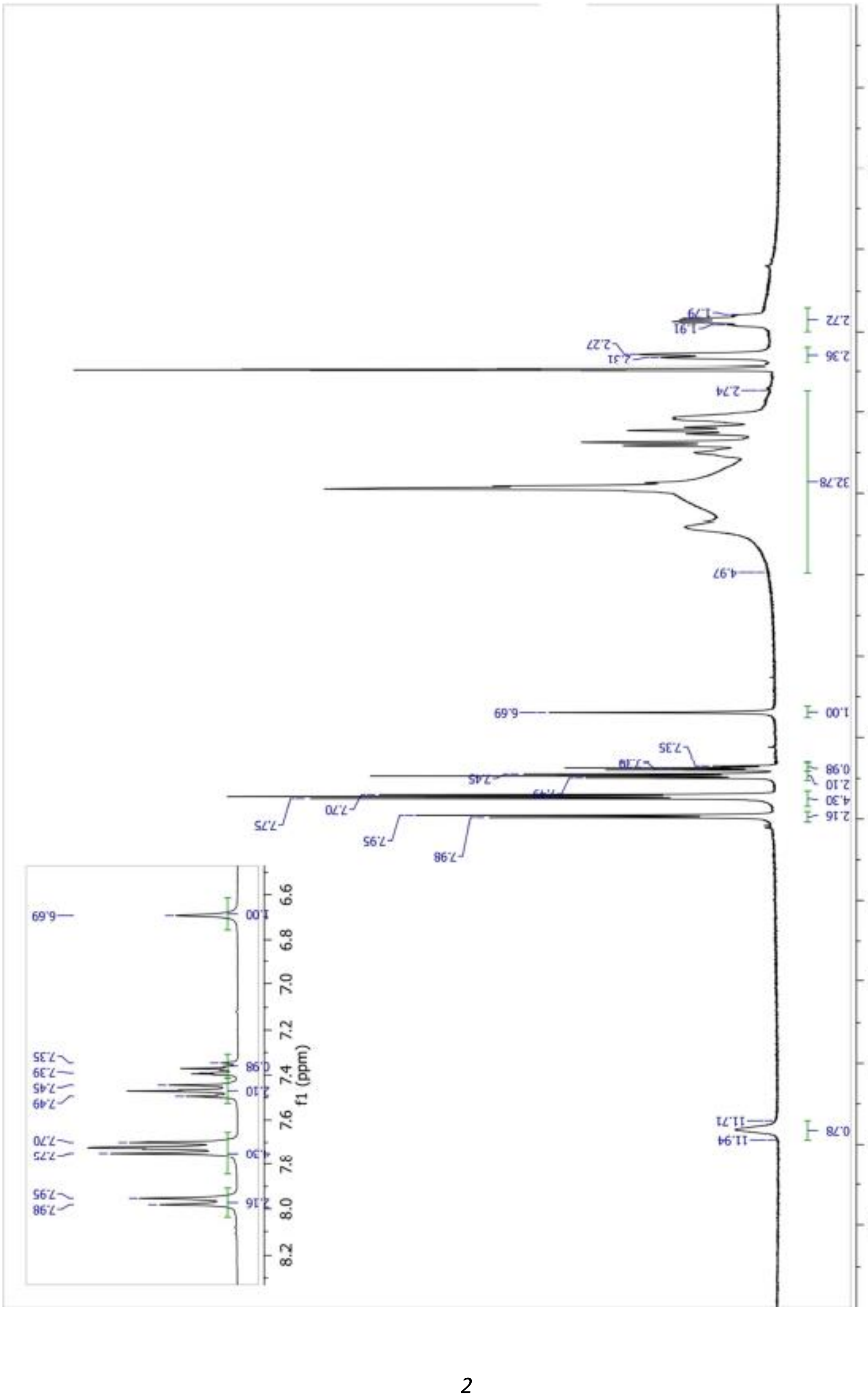

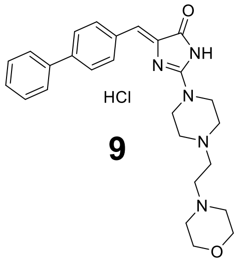


**9**

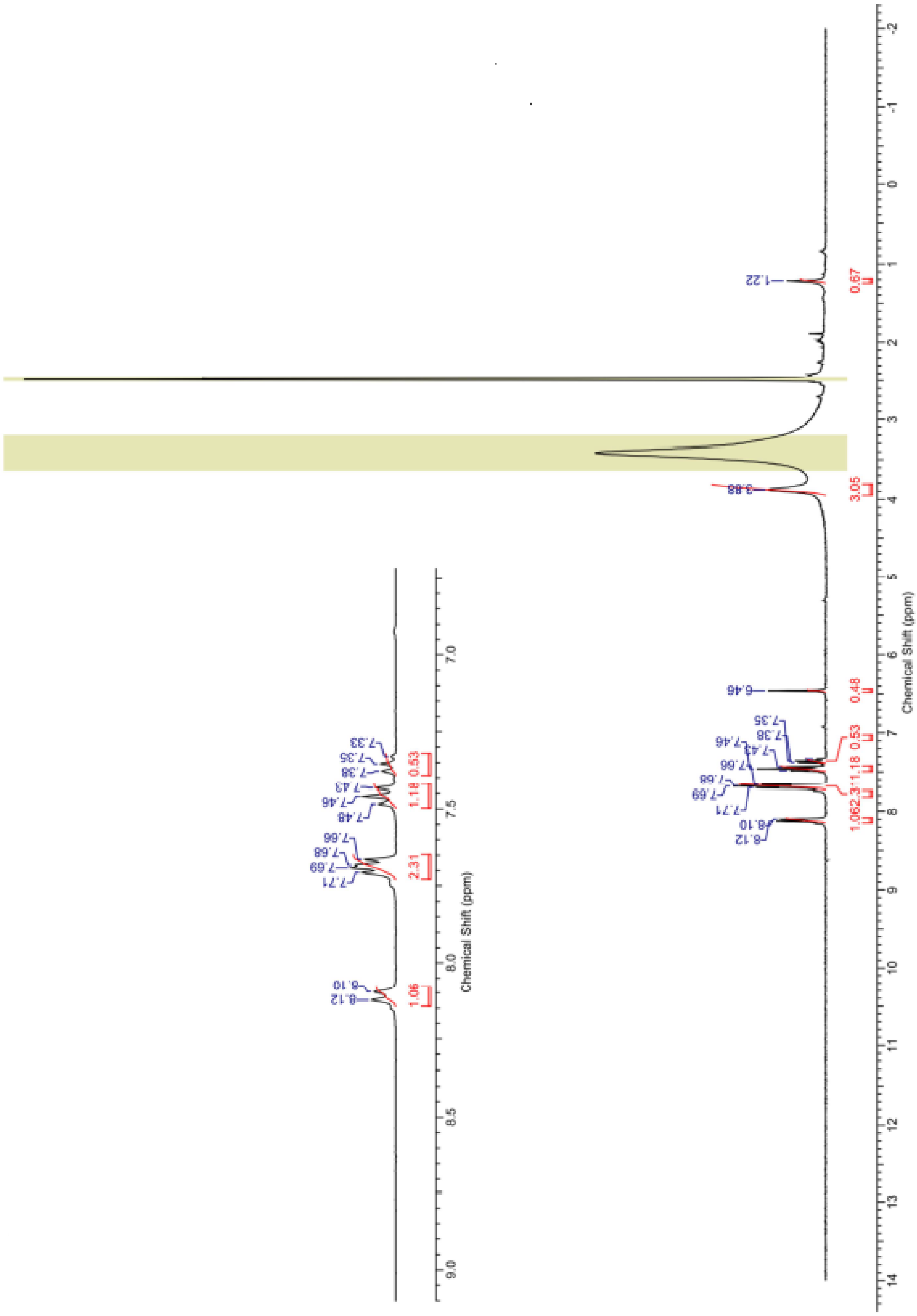

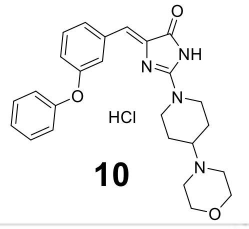

**10**

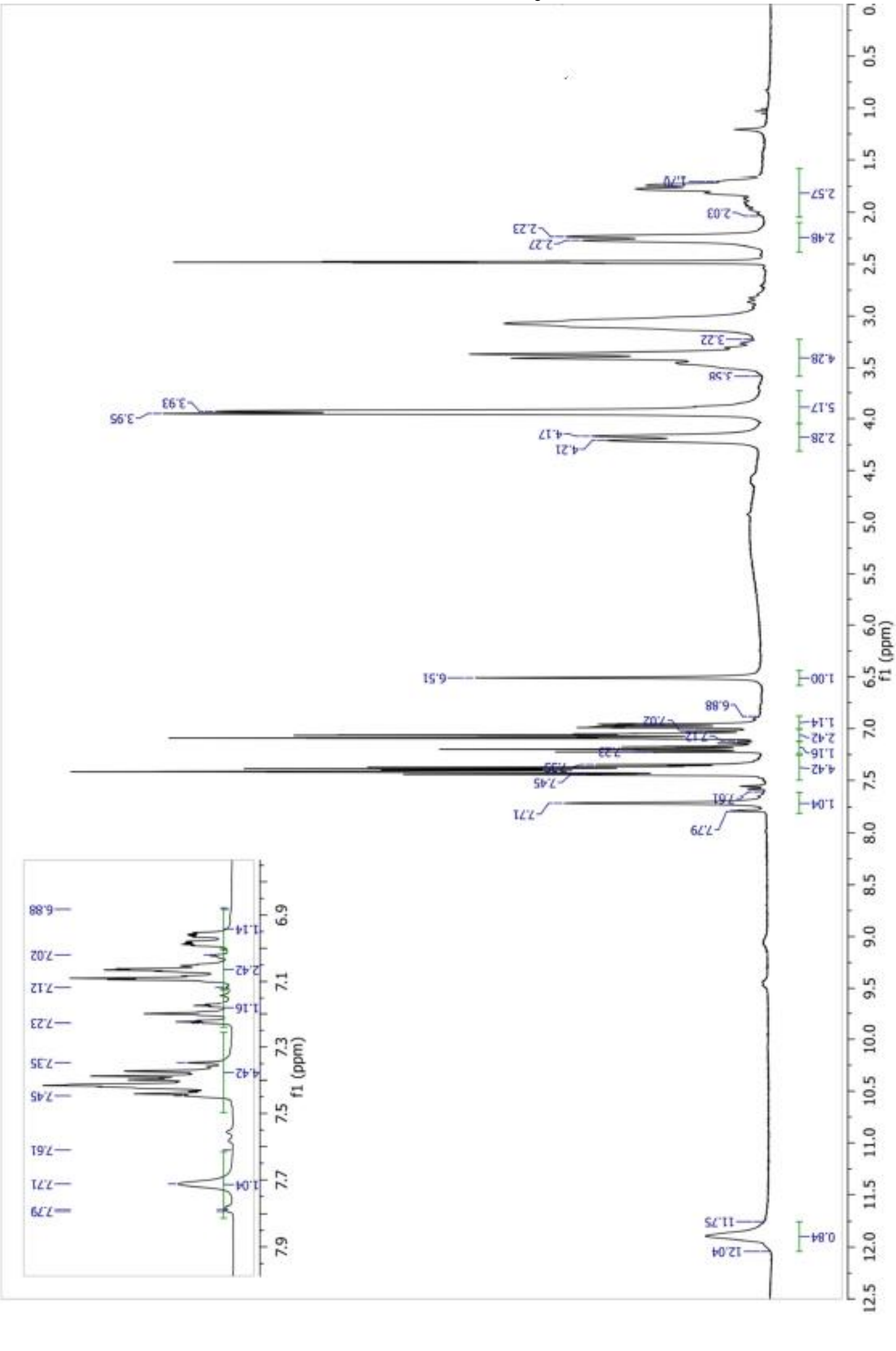

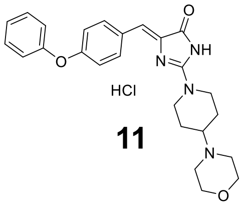


**11**

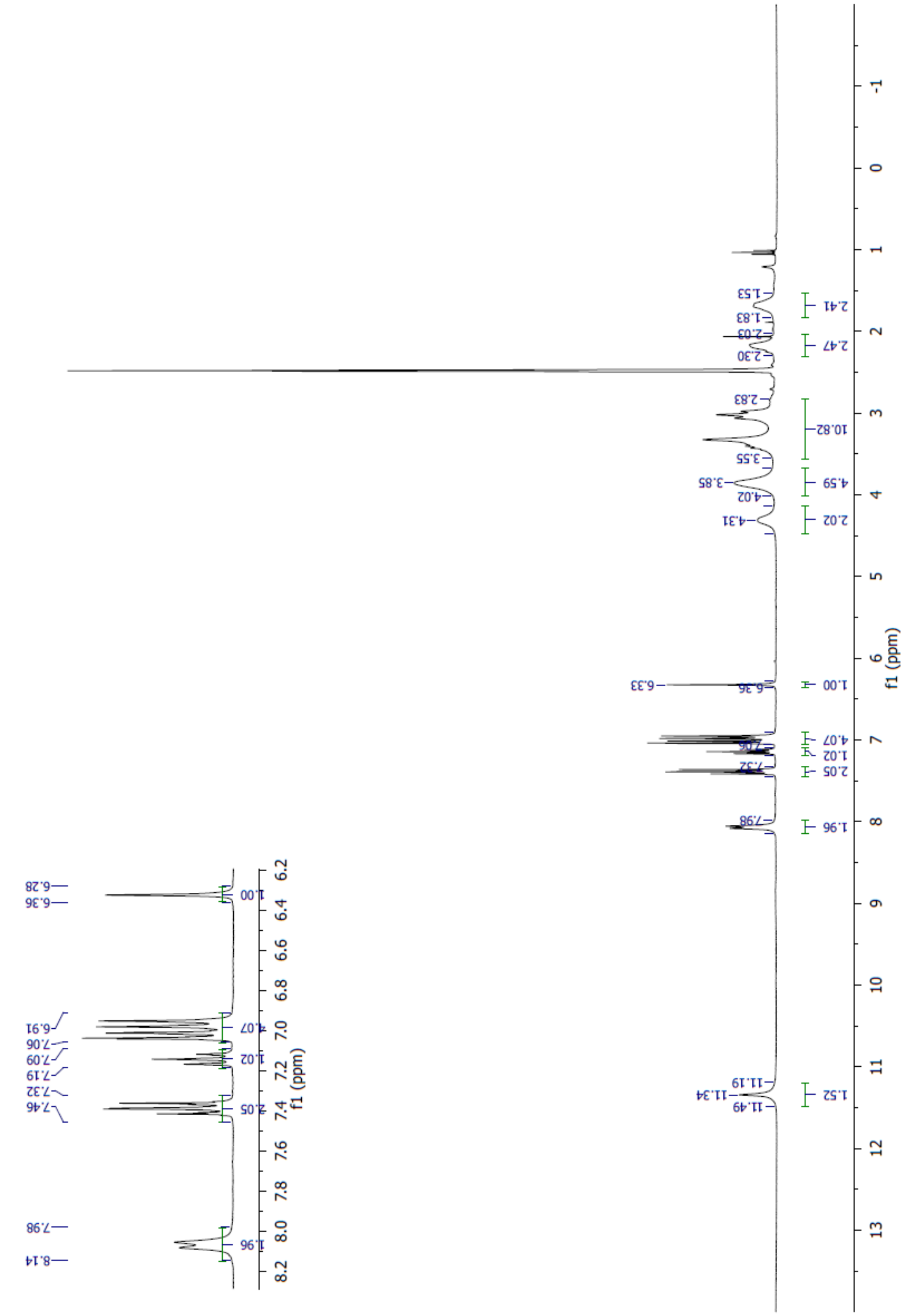

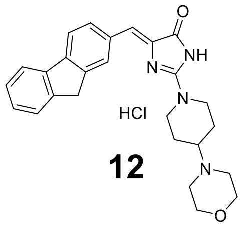


**12**

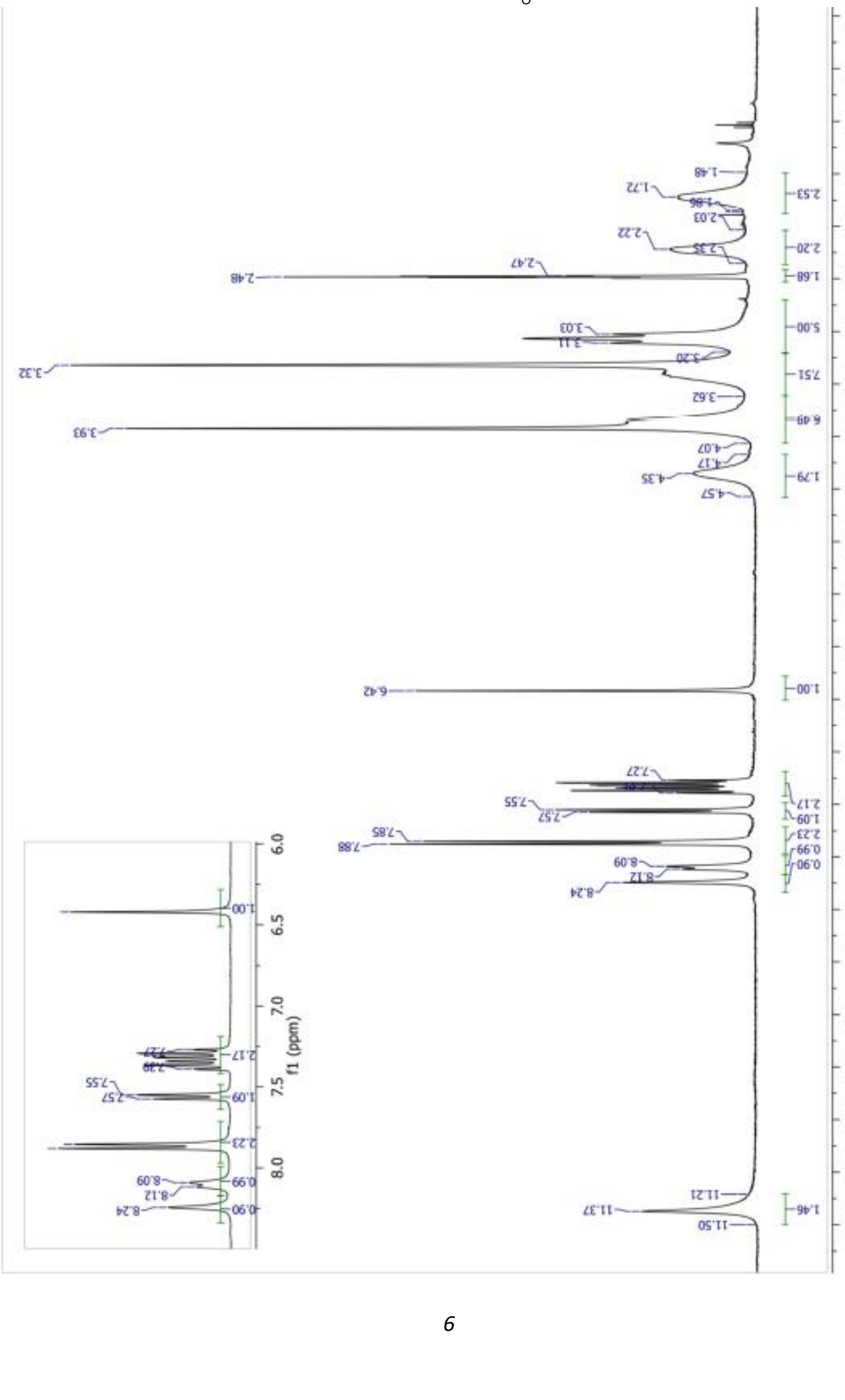

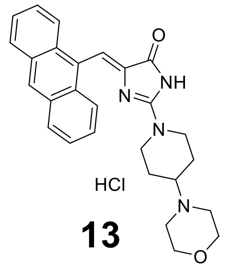


**13**

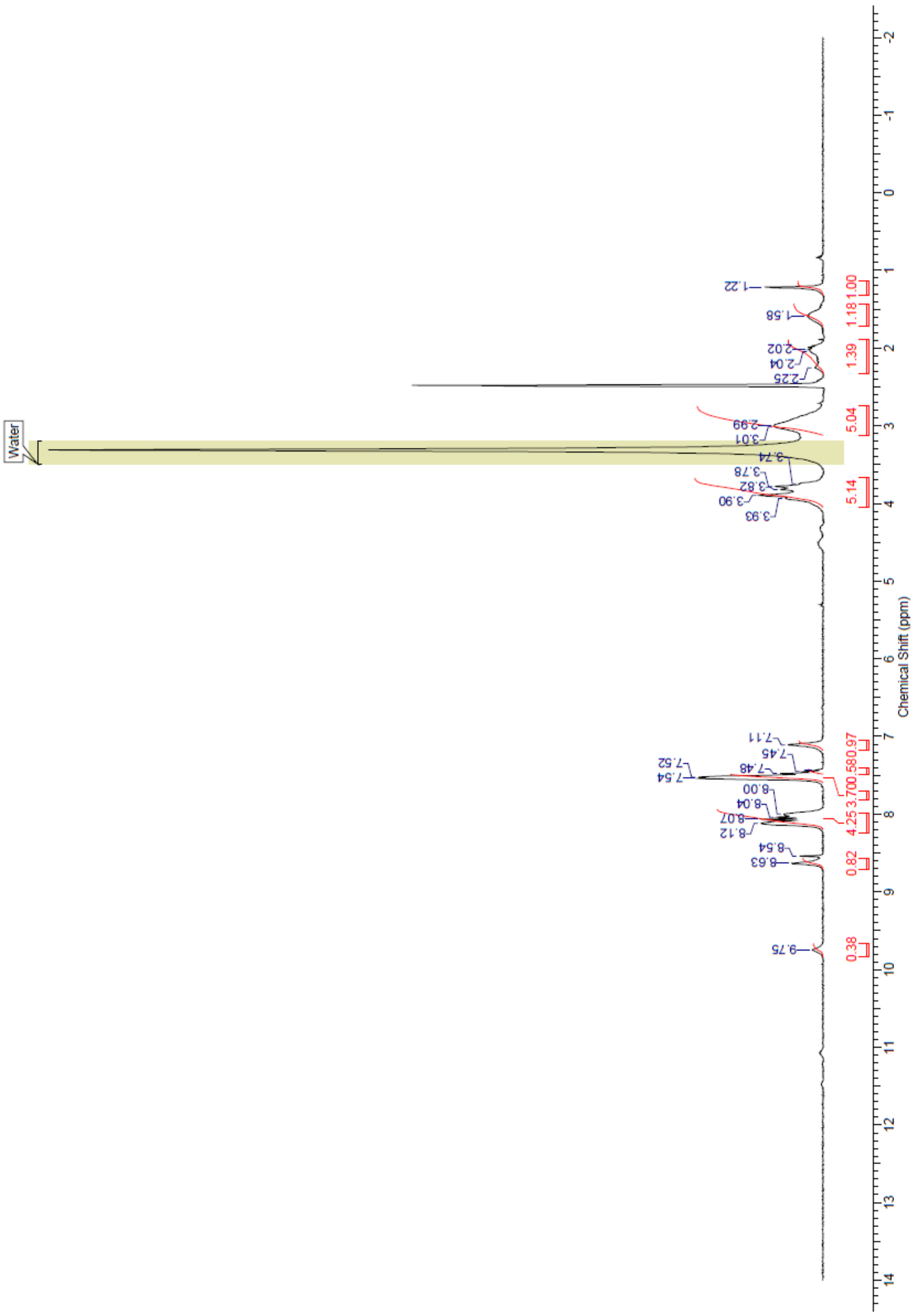

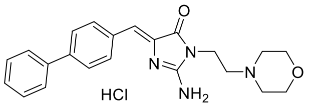

**14**

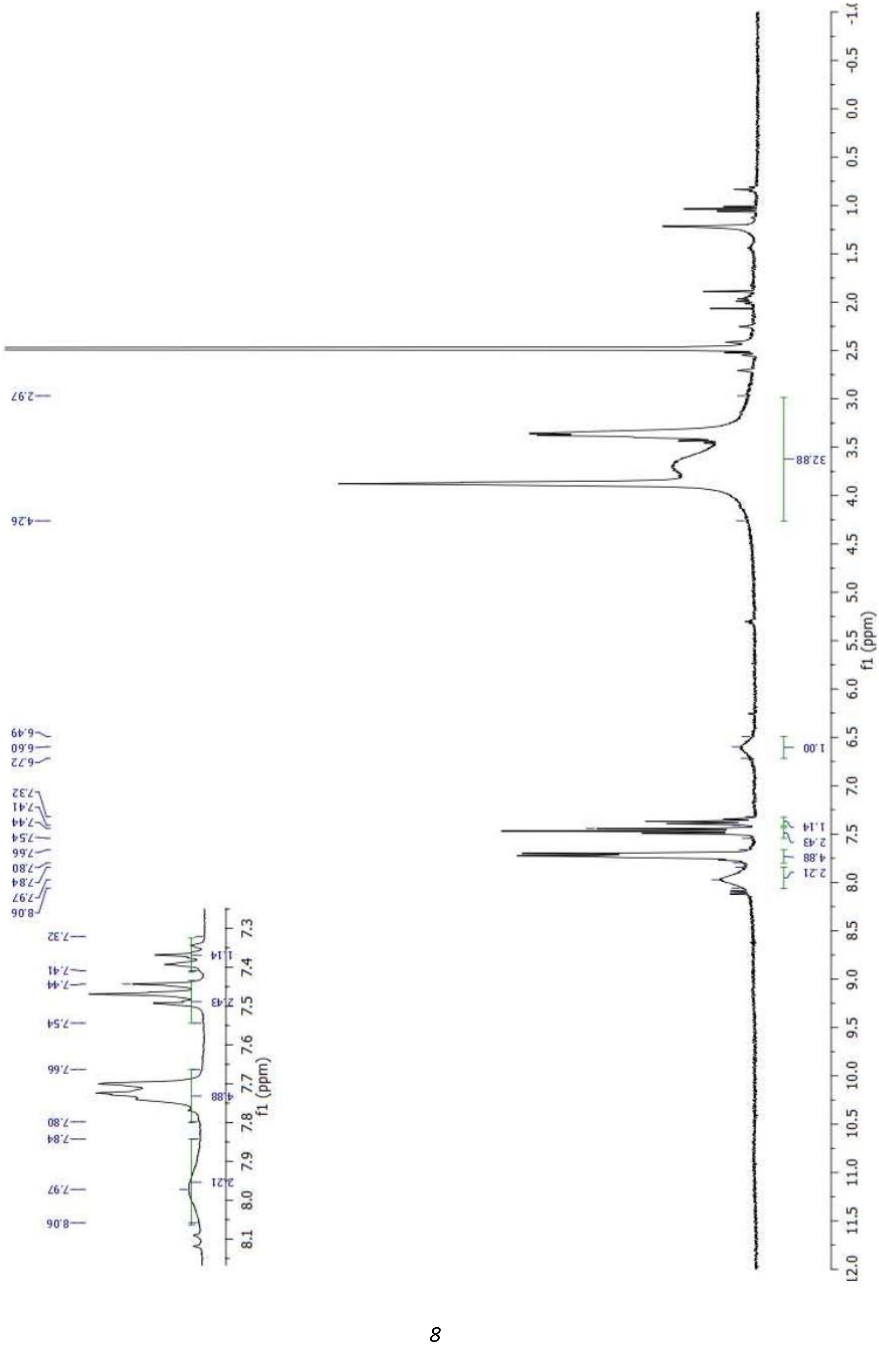

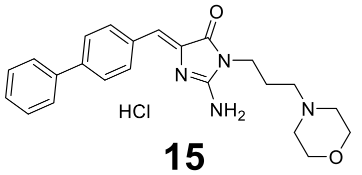

**15**

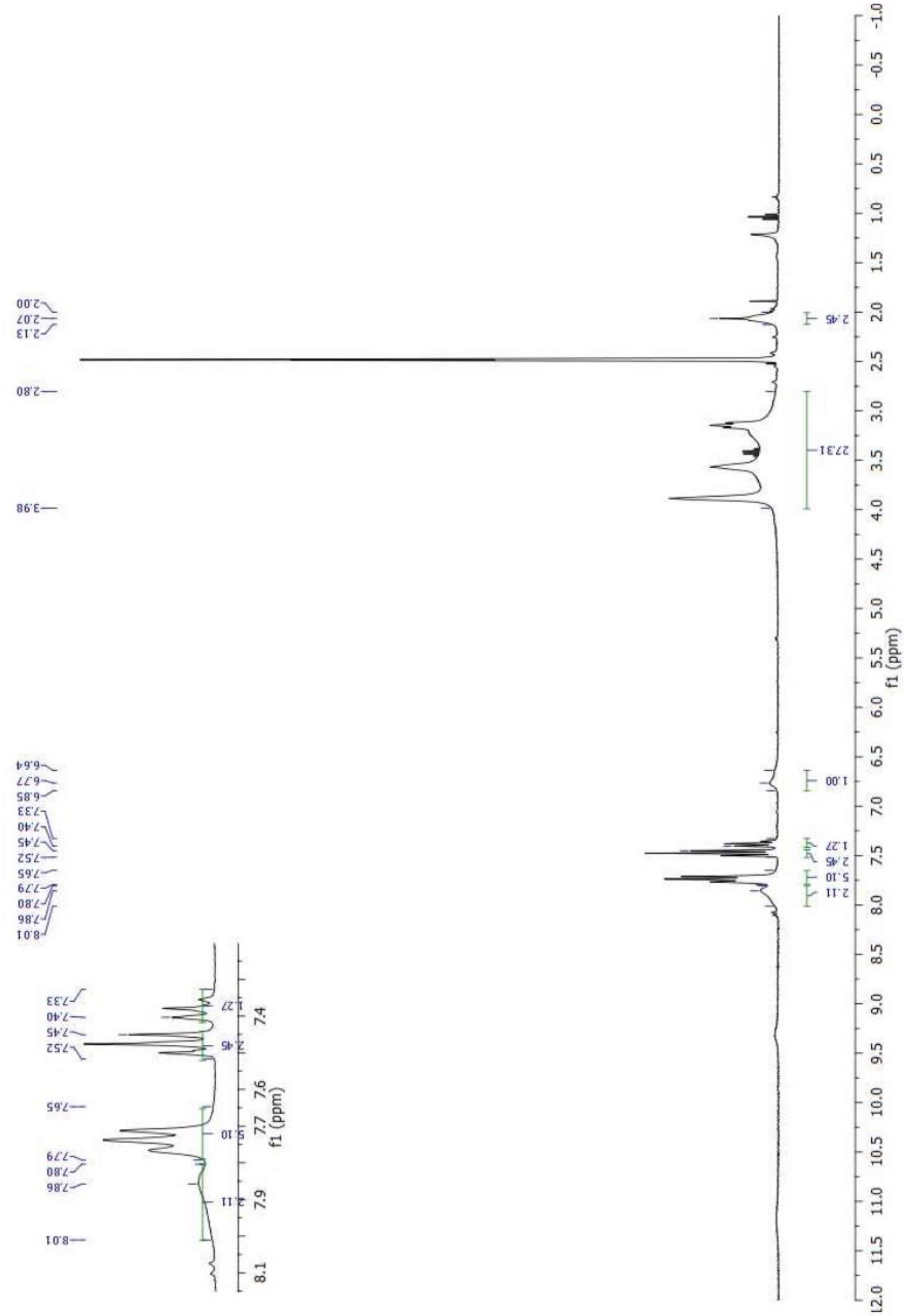

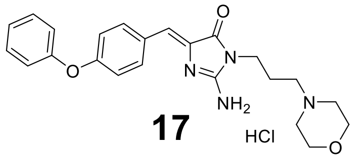

O
N
N
NH2
HCl
N
O
O


**17**

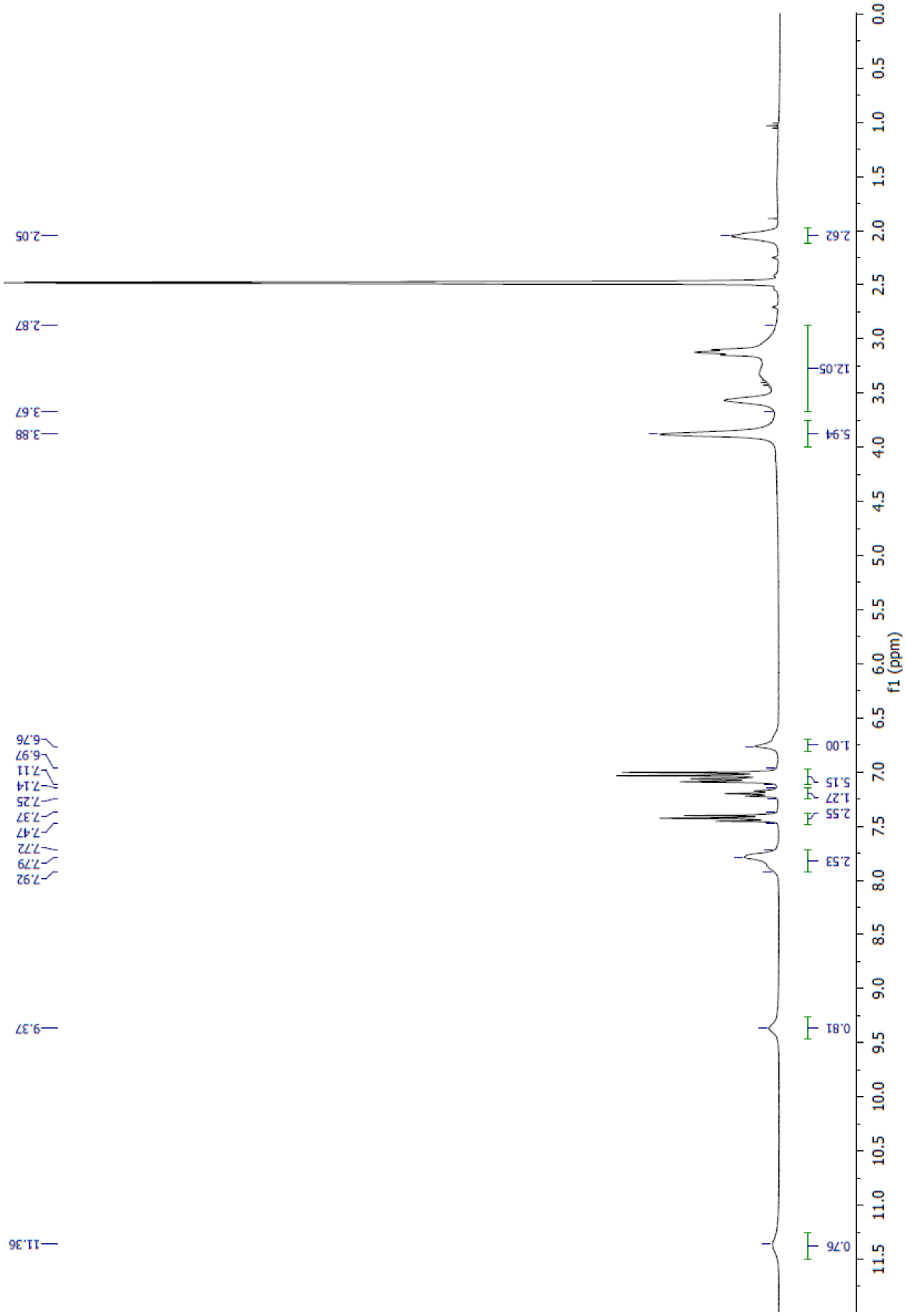

f1 (ppm)
2.05
2.87
3.67
3.88
6.76
6.97
7.11
7.14
7.25
7.37
7.47
7.72
7.79
7.92
9.37
11.36
2.62
12.05
5.94
1.00
5.15
1.27
2.55
2.53
0.81
0.76

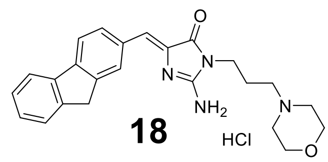


**18**

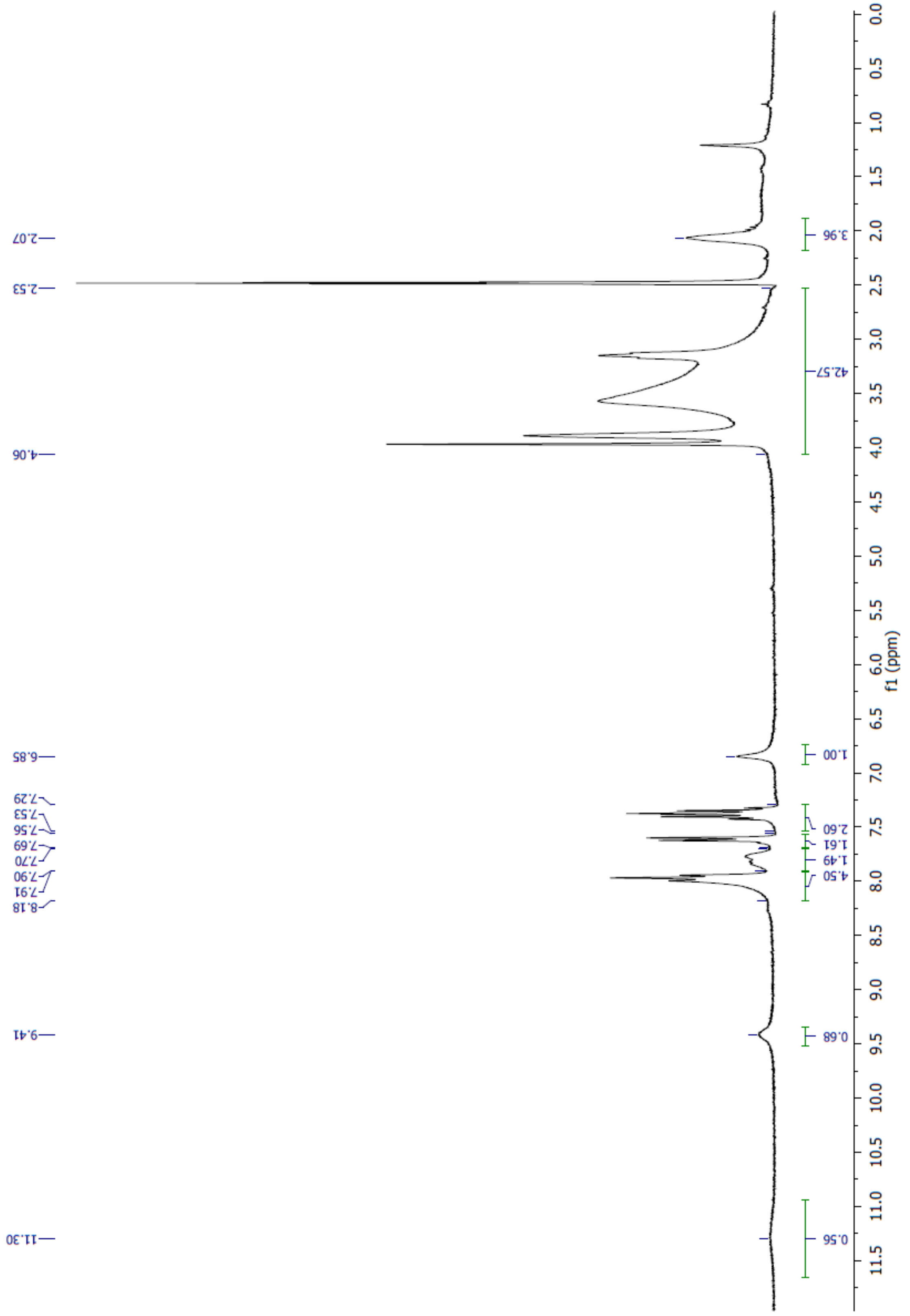

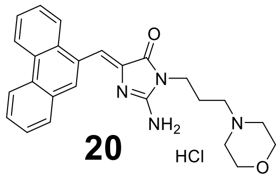

O
N
N
NH2
N
O
HCl


**20**

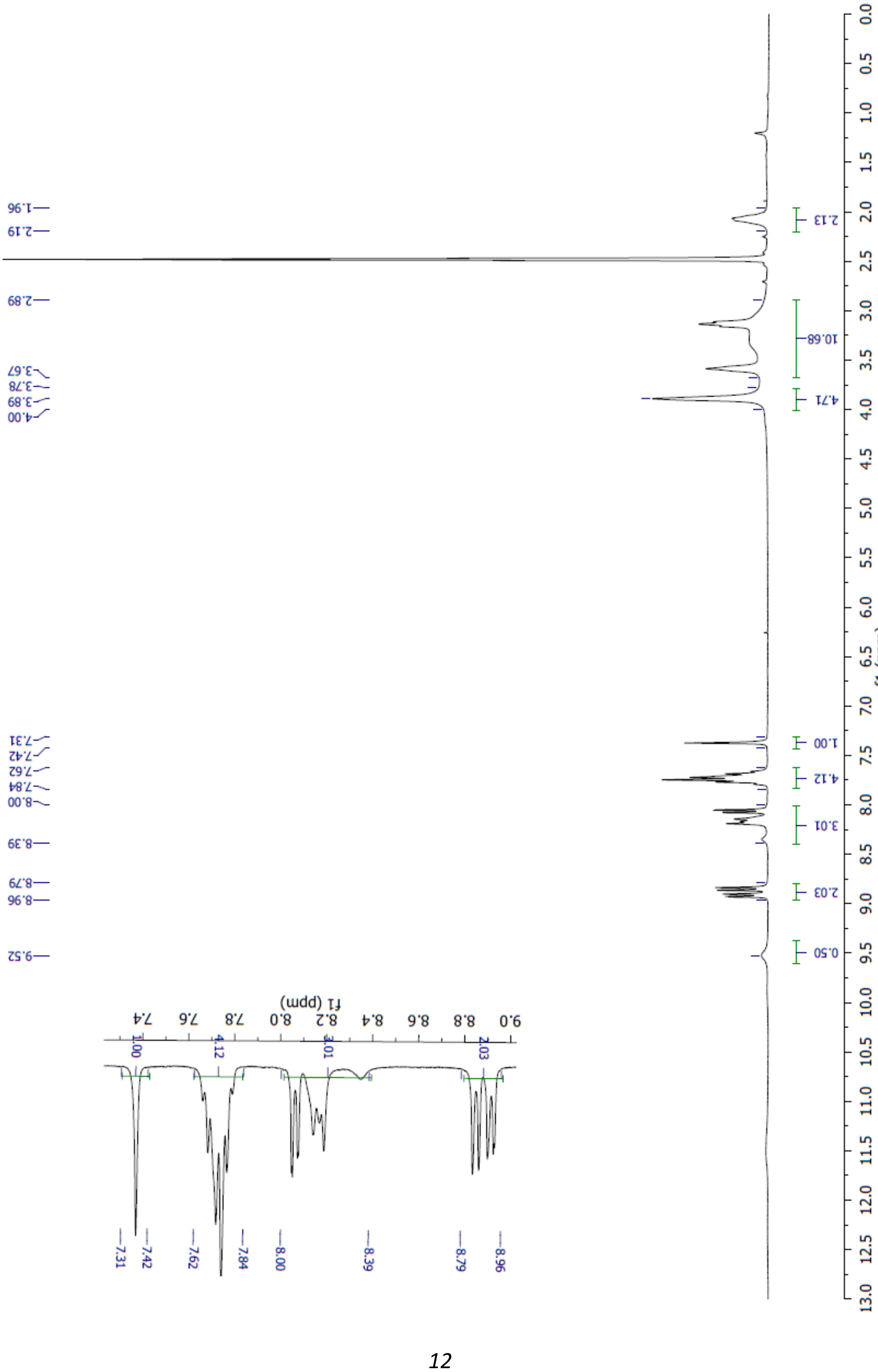

f1 (ppm)
1.96
2.19
2.89
3.67
3.78
3.89
4.00
7.31
7.42
7.62
7.84
8.00
8.39
8.79
8.96
9.52
2.13
10.68
4.71
1.00
4.12
3.01
2.03
0.50

**$^{13}$C NMRs of selected compounds**

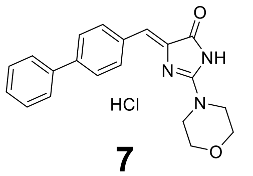


**7**

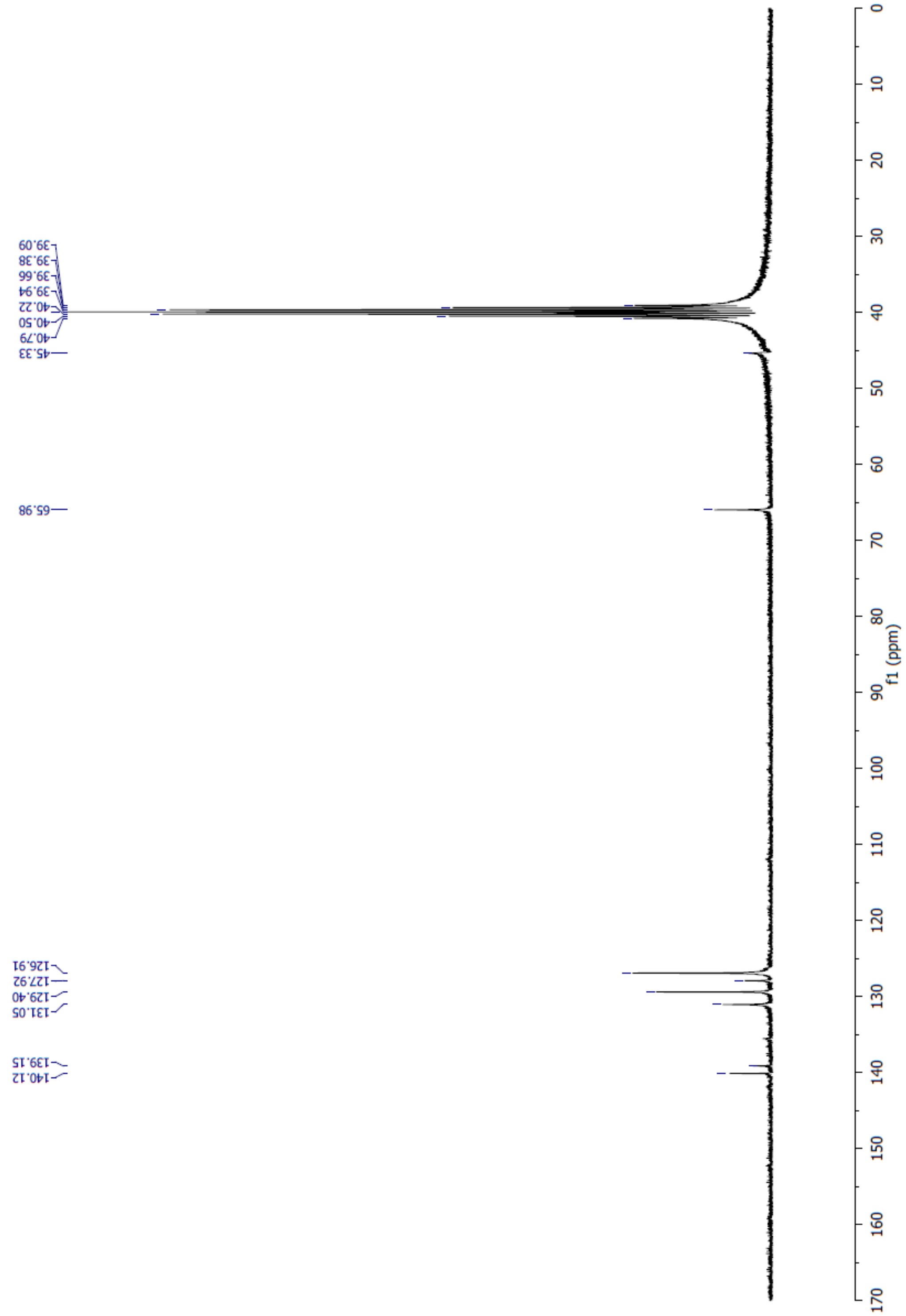

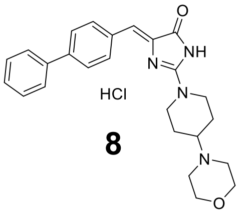

HCl


**8**

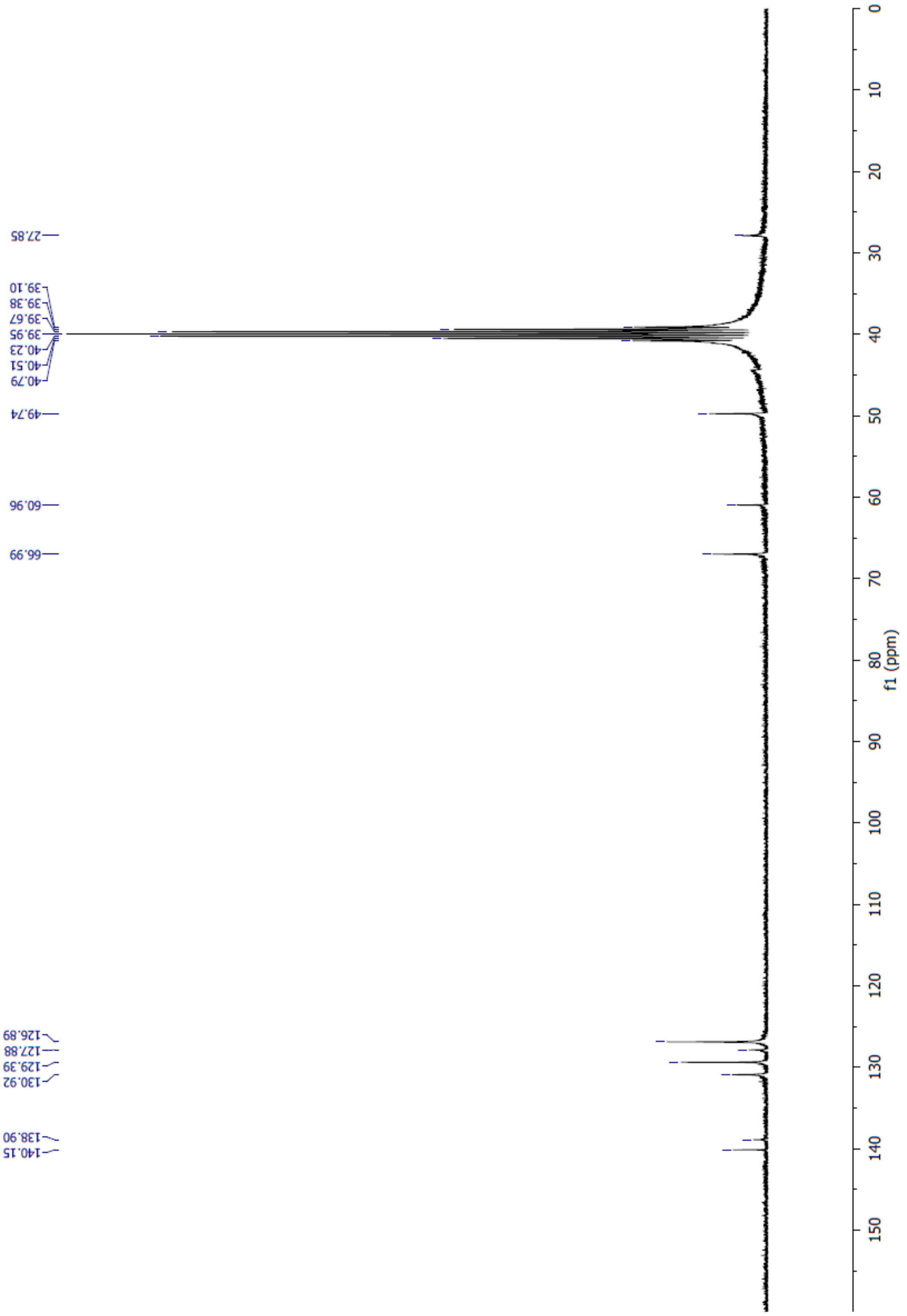

27.85
39.10
39.38
39.67
39.95
40.23
40.51
40.79
49.74
60.96
66.99
126.89
127.88
129.39
130.92
138.90
140.15
f1 (ppm)

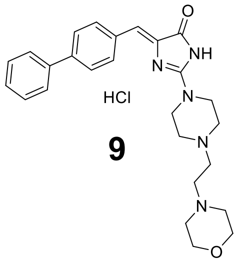
O
NH
N
N
N
HCl
N
O
9

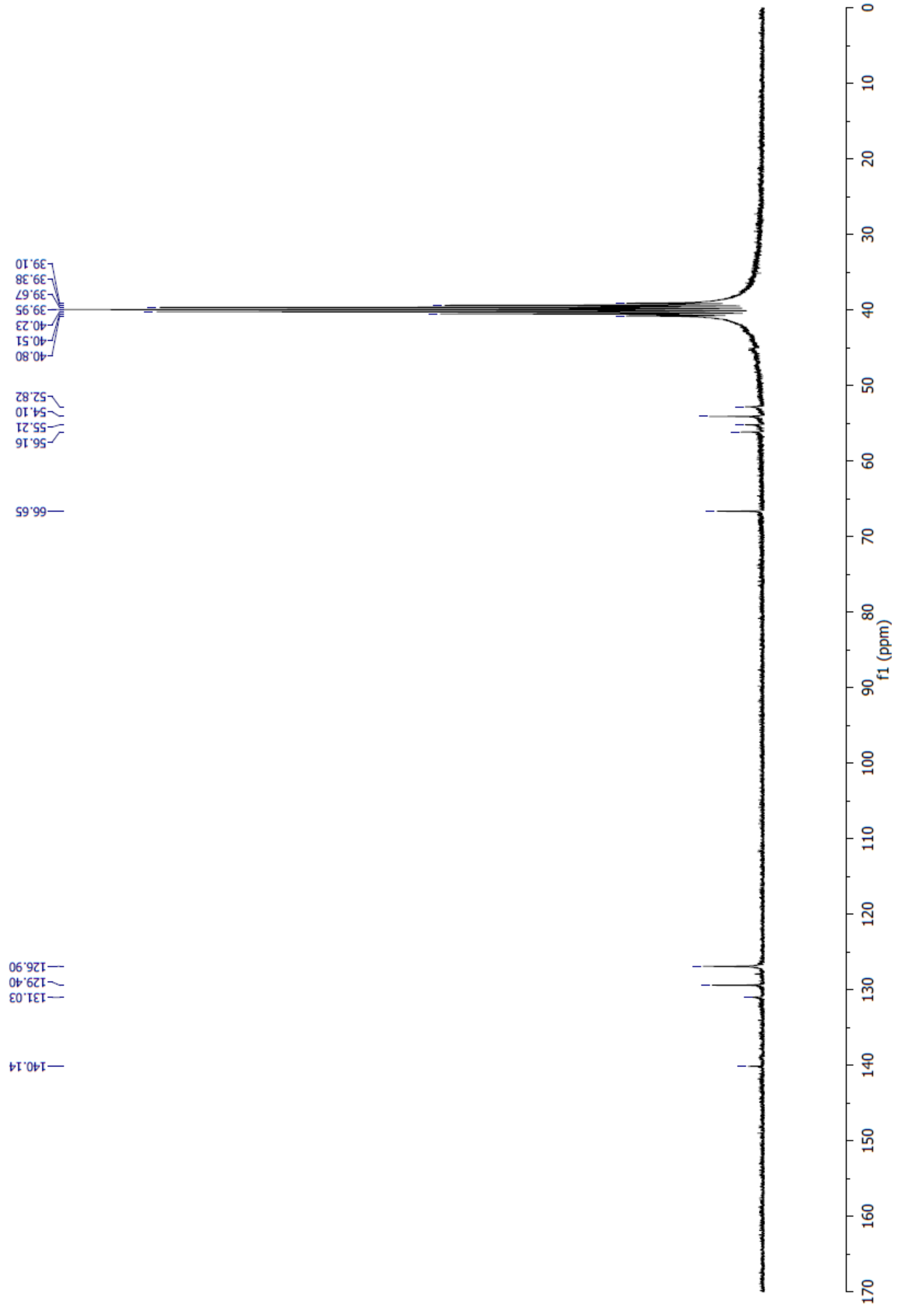
39.10
39.38
39.67
39.95
40.23
40.51
40.80
52.82
54.10
55.21
56.16
66.65
126.90
129.40
131.03
140.14
f1 (ppm)

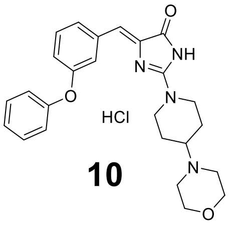
O
NH
N
N
O
HCl
N
O

**10**

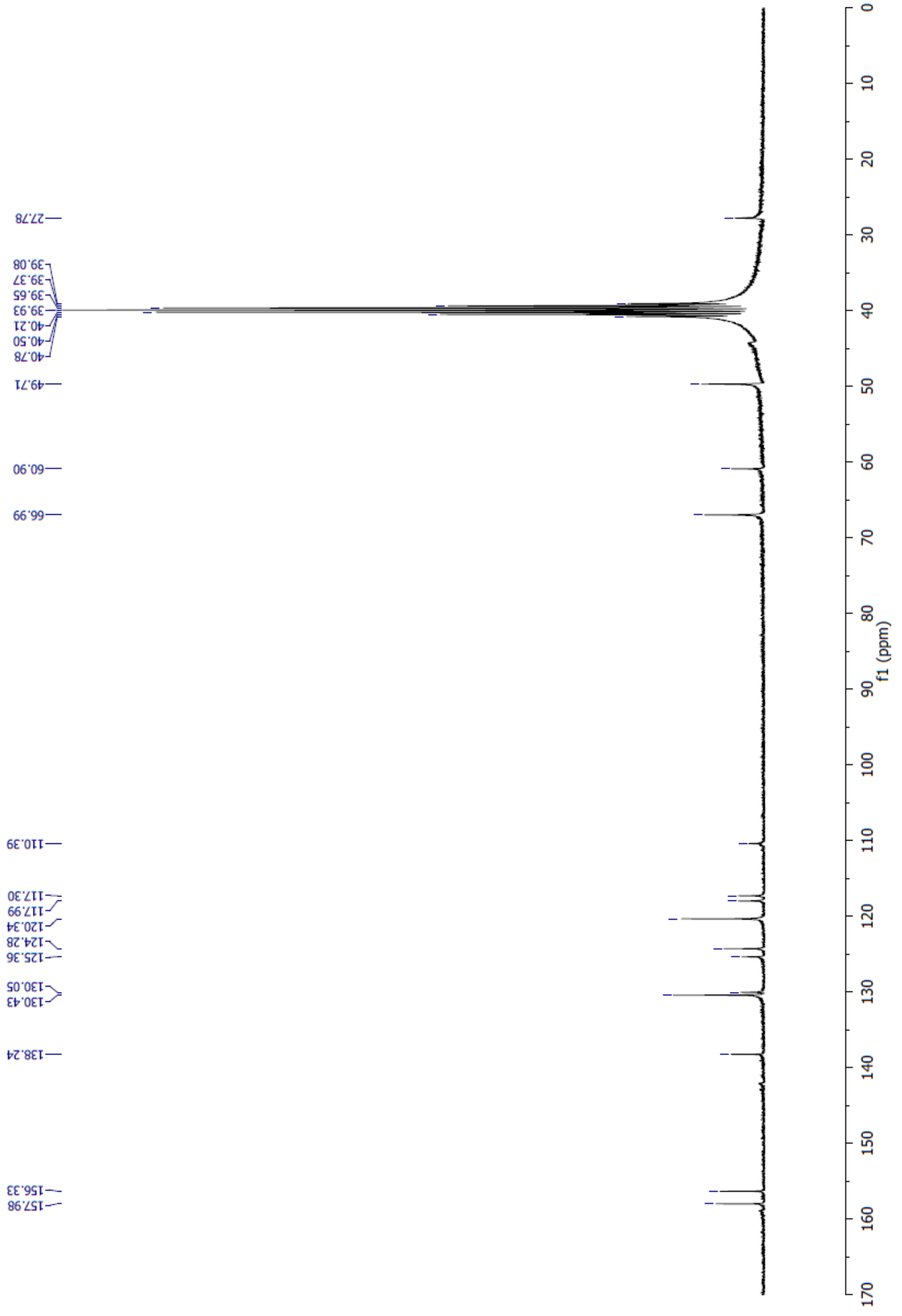
27.78
39.08
39.37
39.65
39.93
40.21
40.50
40.78
49.71
60.90
66.99
110.39
117.30
117.99
120.34
124.28
125.36
130.05
130.43
138.24
156.33
157.98
f1 (ppm)
0
10
20
30
40
50
60
70
80
90
100
110
120
130
140
150
160
170

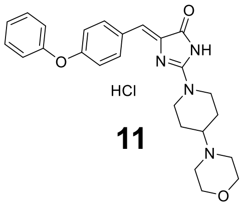
O
NH
N
O
HCl
N
N
O
11

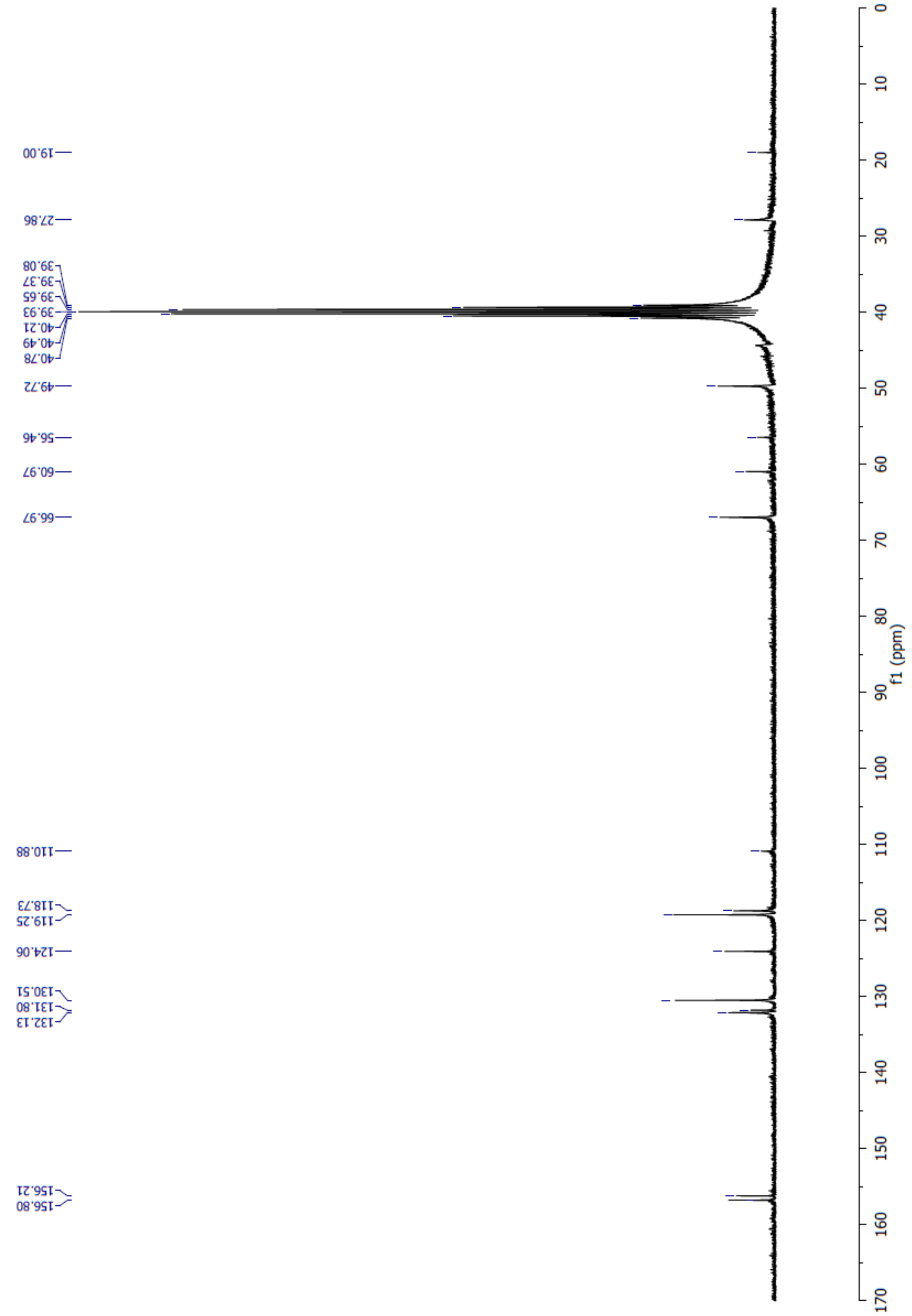
19.00
27.86
39.08
39.37
39.65
39.93
40.21
40.49
40.78
49.72
56.46
60.97
66.97
110.88
118.73
119.25
124.06
130.51
131.80
132.13
156.21
156.80
f1 (ppm)
0
10
20
30
40
50
60
70
80
90
100
110
120
130
140
150
160
170

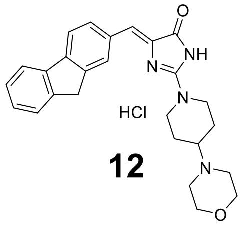
O
NH
N
N
N
O
HCl
12

**12**

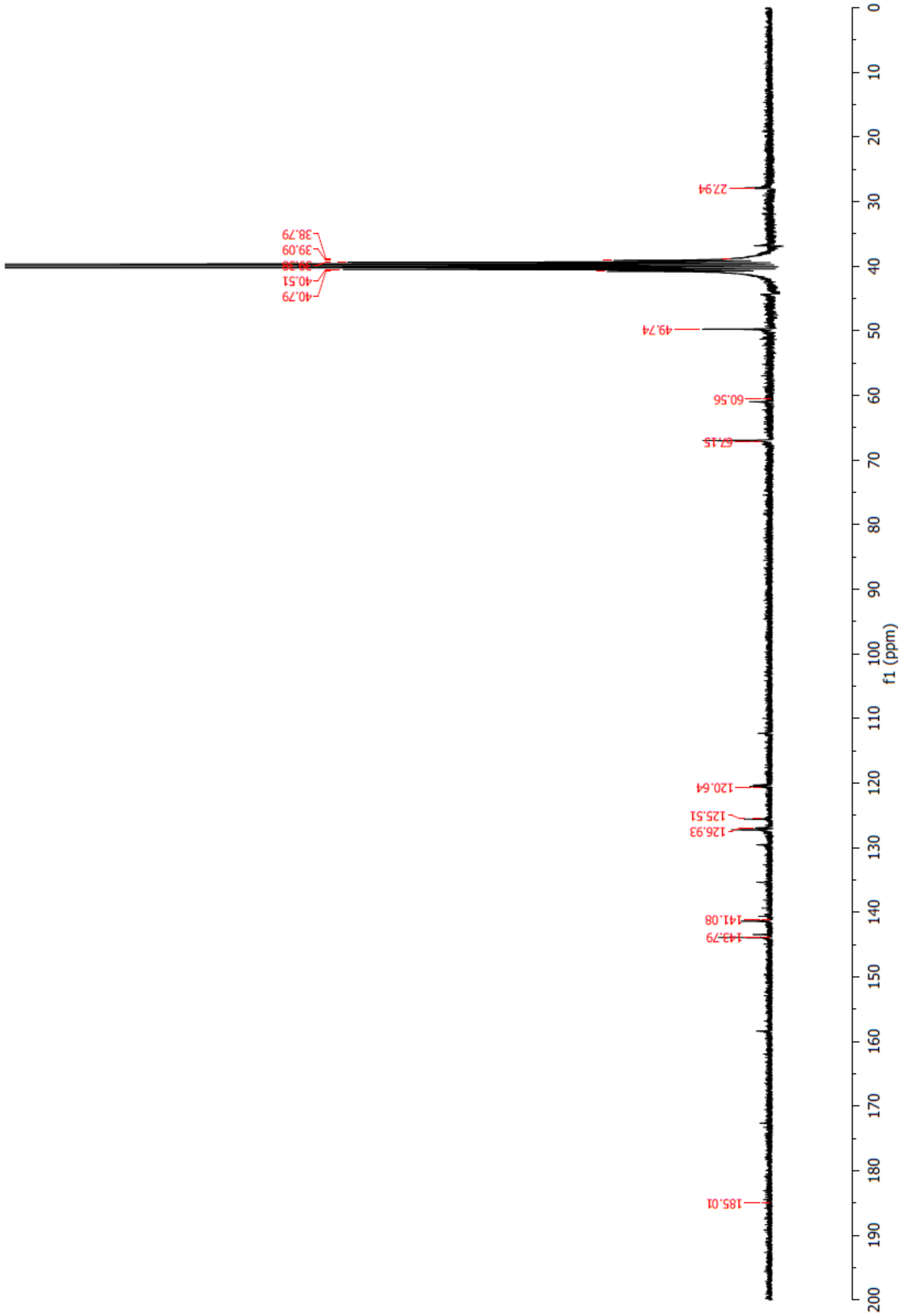
27.94
38.79
39.09
39.38
40.51
40.79
49.74
60.56
67.15
120.64
125.51
126.93
141.08
143.79
185.01
f1 (ppm)
0
10
20
30
40
50
60
70
80
90
100
110
120
130
140
150
160
170
180
190
200

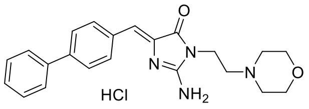
HCl
NH2

**14**

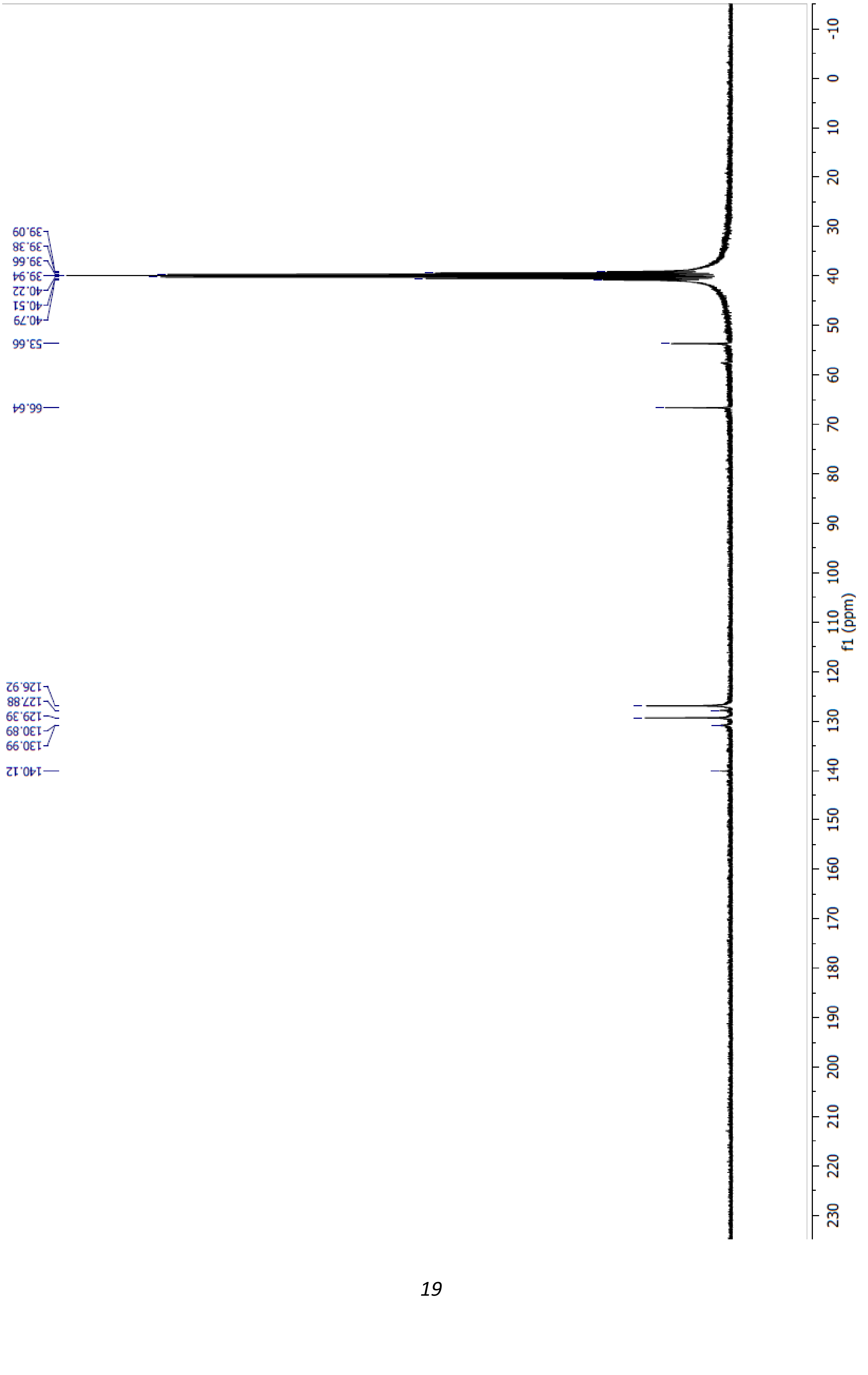
39.09
39.38
39.66
39.94
40.22
40.51
40.79
53.66
66.64
126.92
127.88
129.39
130.89
130.99
140.12
f1 (ppm)

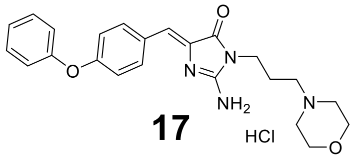

O
N
N
NH2
HCl
N
O


**17**

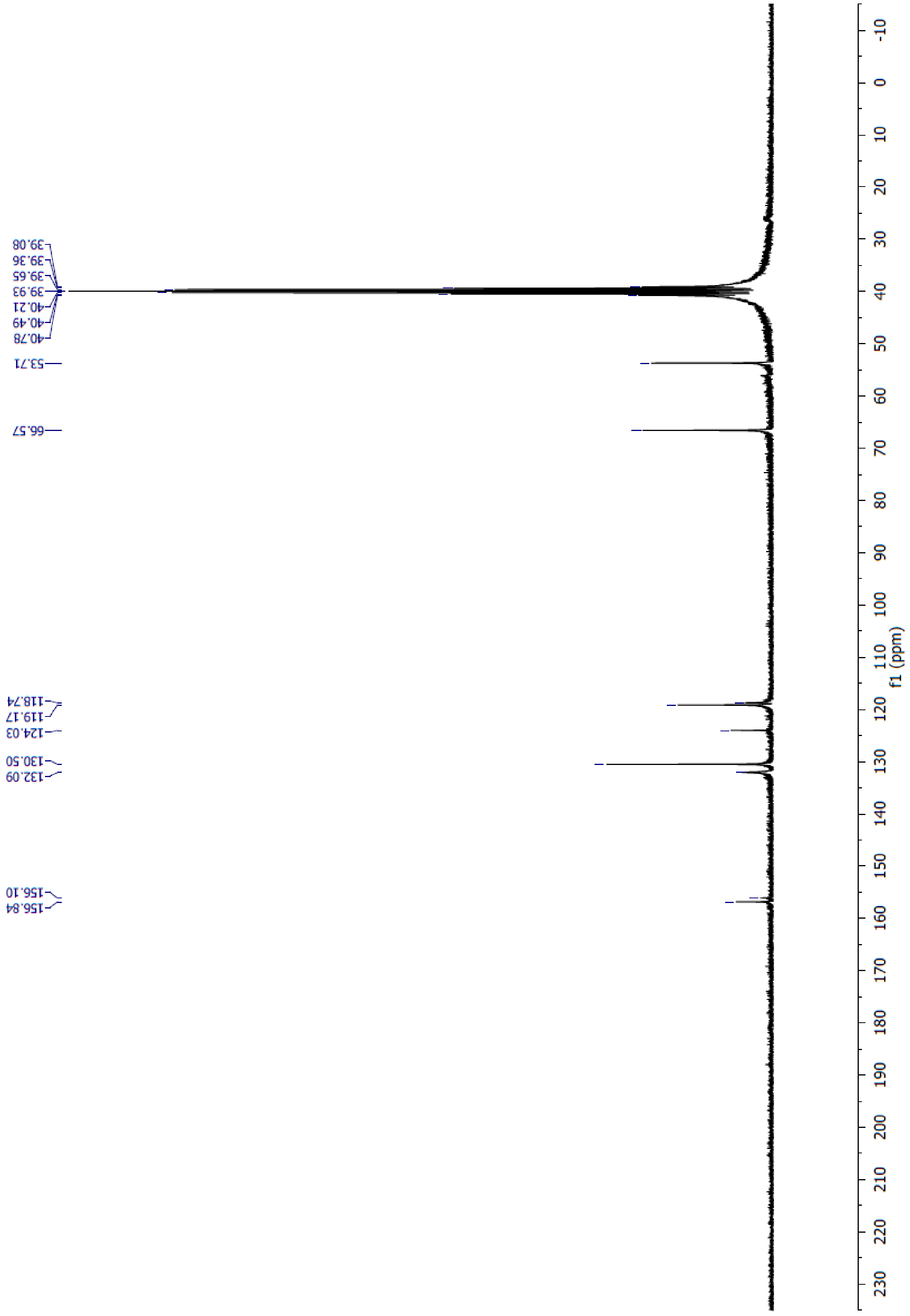

39.08
39.36
39.65
39.93
40.21
40.49
40.78
53.71
66.57
118.74
119.17
124.03
130.50
132.09
156.10
156.84
f1 (ppm)

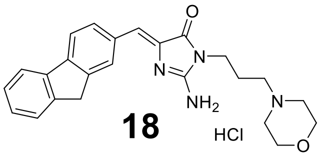
O
N
N
NH2
HCl
N
O

**18**

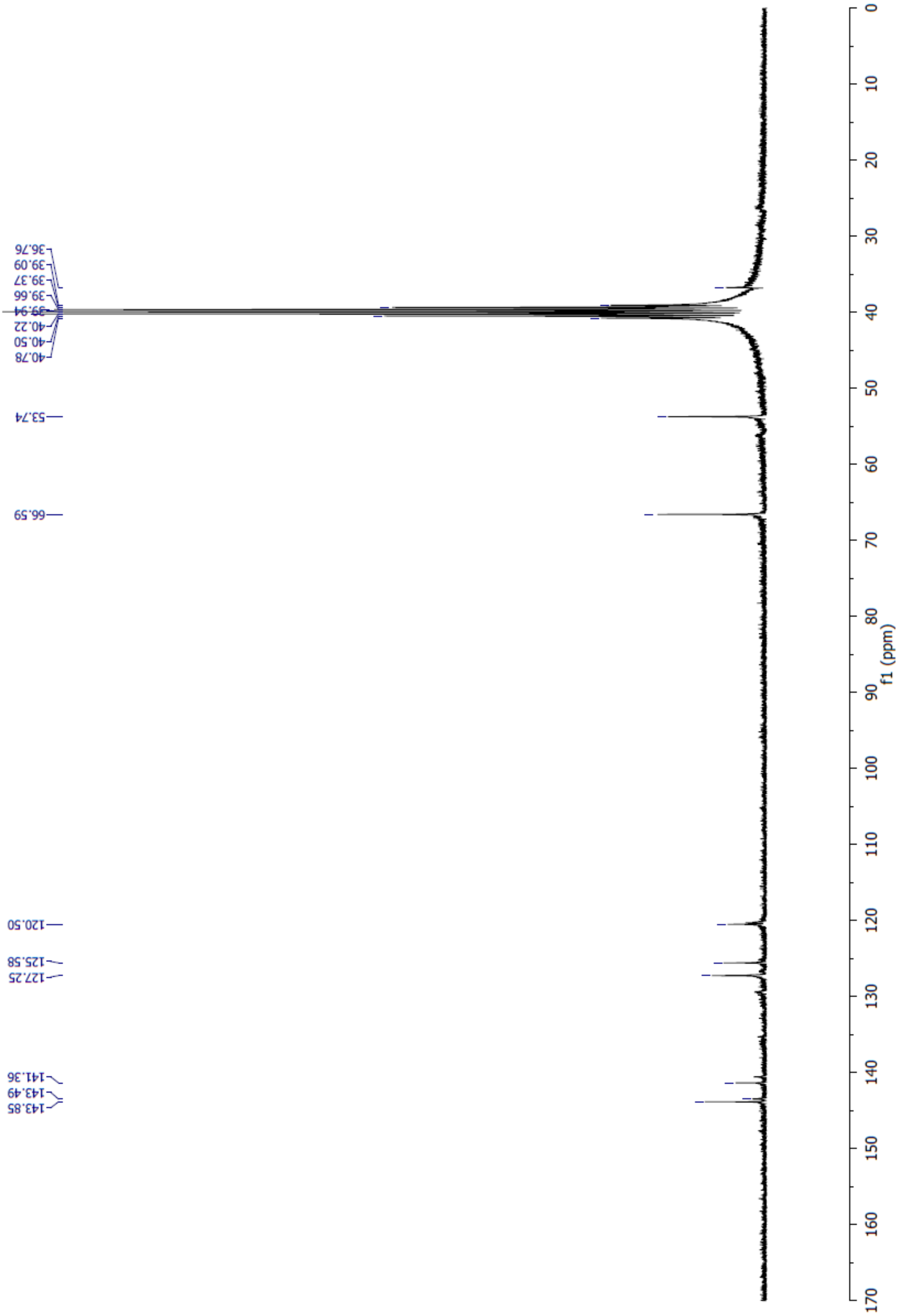
36.76
39.09
39.37
39.66
39.94
40.22
40.50
40.78
53.74
66.59
120.50
125.58
127.25
141.36
143.49
143.85
f1 (ppm)

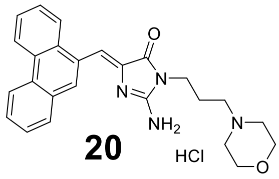
O
N
N
NH2
HCl
N
O

**20**

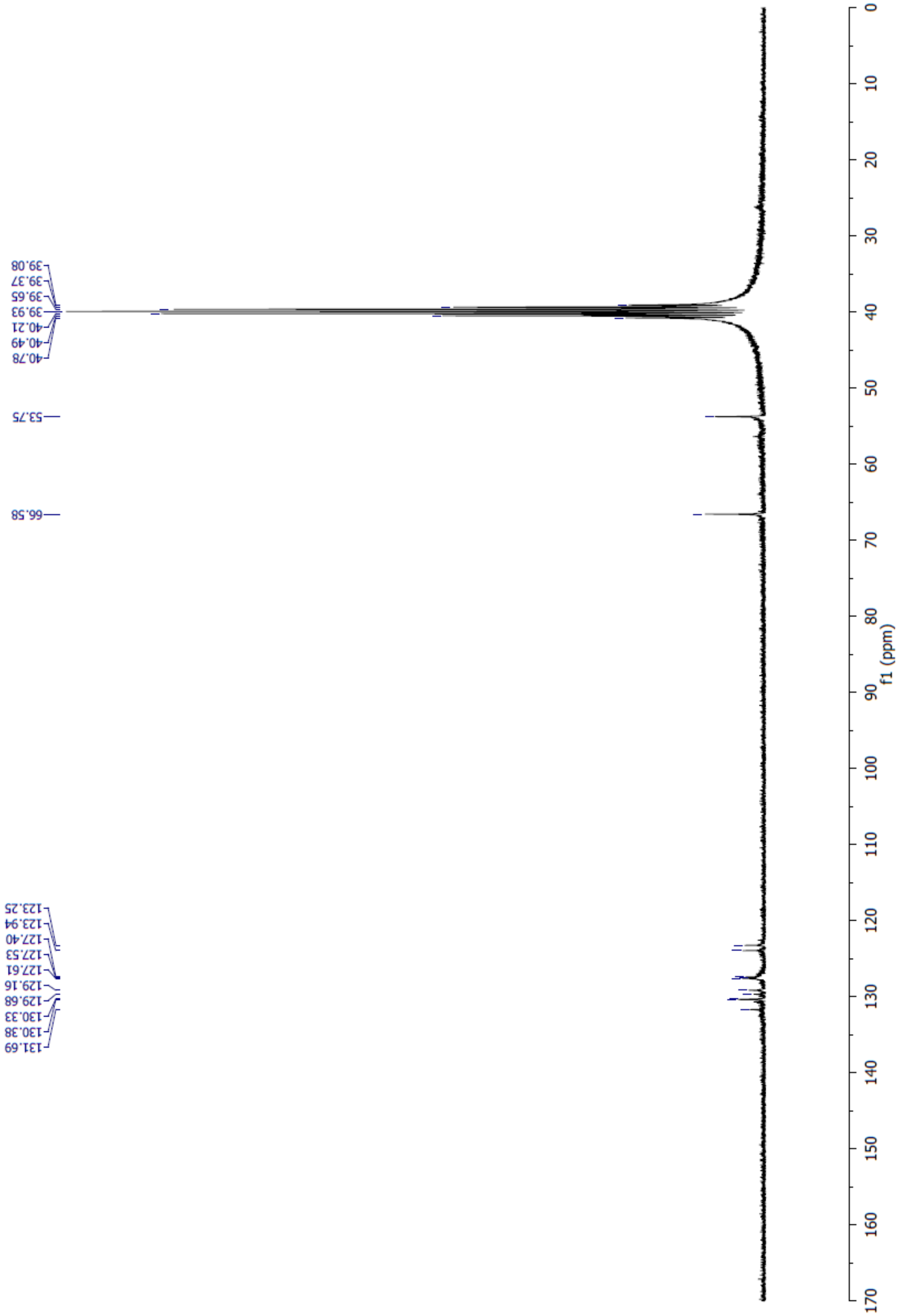
39.08
39.37
39.65
39.93
40.21
40.49
40.78
53.75
66.58
123.25
123.94
127.40
127.53
127.61
129.16
129.68
130.33
130.38
131.69
f1 (ppm)

## Homology modeling

The homology model of human P-glycoprotein was constructed by multiple comparative modeling methods using the X-ray structures of murine Pgp: 4Q9H, 4XWK and 4M1M as templates (resolution 3.4 Å, 3.5 Å and 3.8 Å, respectively). Out of 1000 models generated by MODELLER software,[1] three models with the lowest DOPE scores (-154 032.45, -153 981.16, -153 884.80) were refined by minimization of energy in Schrodinger Suite software.[2] Analysis of the Ramachandran plots for all three models allowed to choose the most beneficial homology model showing the highest number of residues in most favored regions (1077; 93.7%) and the lowest number of residues in disallowed regions (4; 0.3%), with the DOPE score -153 884.80 (**Fig. S1a**). Alignment of this model to individual templates performed in the Maestro module[3] gave the following results:

4Q9H Alignment Score: 0.031, RMSD: 0.868 Å

4XWK Alignment Score: 0.043, RMSD: 1.020 Å

4M1M Alignment Score: 0.444, RMSD: 2.946 Å

The model was subjected to further structure validation using PROCHECK,[4] ProSA[5] and QMEAN[6, 7] software (**Fig. S2** and **Fig. S3**). Incorporation of the linker region with a predicted secondary structure (amino acids 630-698) into the selected top ranked homology model allowed to improve its quality and decrease the number of residues found in disallowed regions (**Fig. S1b**).

a) B008 homology model, DOPE -153 884.80

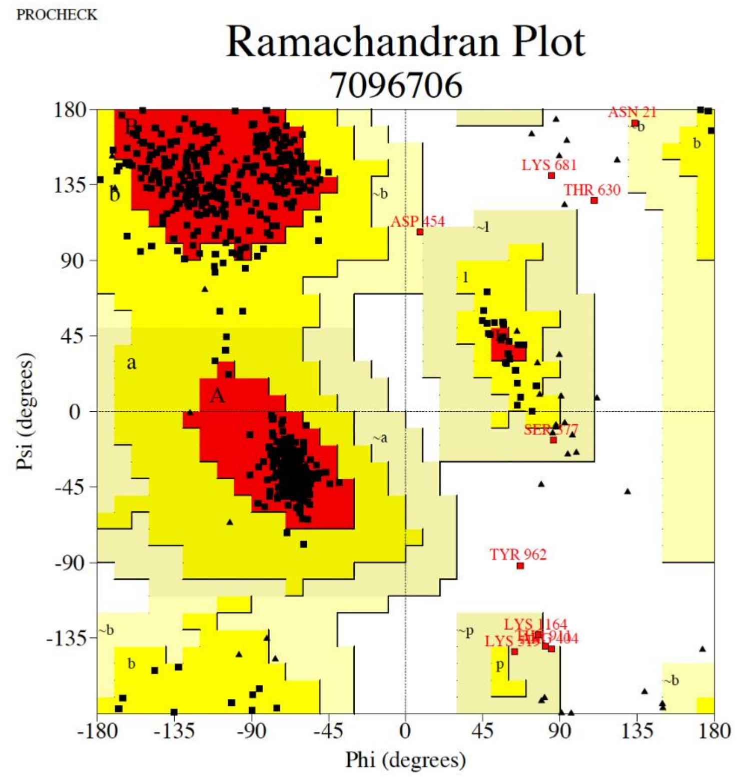


Plot statistics

| | | |
|---|---|---|
| Residues in most favoured regions [A,B,L] | 1077 | 93.7% |
| Residues in additional allowed regions [a,b,l,p] | 63 | 5.5% |
| Residues in generously allowed regions [~a,~b,~l,~p] | 6 | 0.5% |
| Residues in disallowed regions | 4 | 0.3% |
| | ---- | ------ |
| Number of non-glycine and non-proline residues | 1150 | 100.0% |
| Number of end-residues (excl. Gly and Pro) | 2 | |
| Number of glycine residues (shown as triangles) | 99 | |
| Number of proline residues | 29 | |
| | ---- | |
| Total number of residues | 1280 | |

b) Optimized model B008 with a linker incorporated

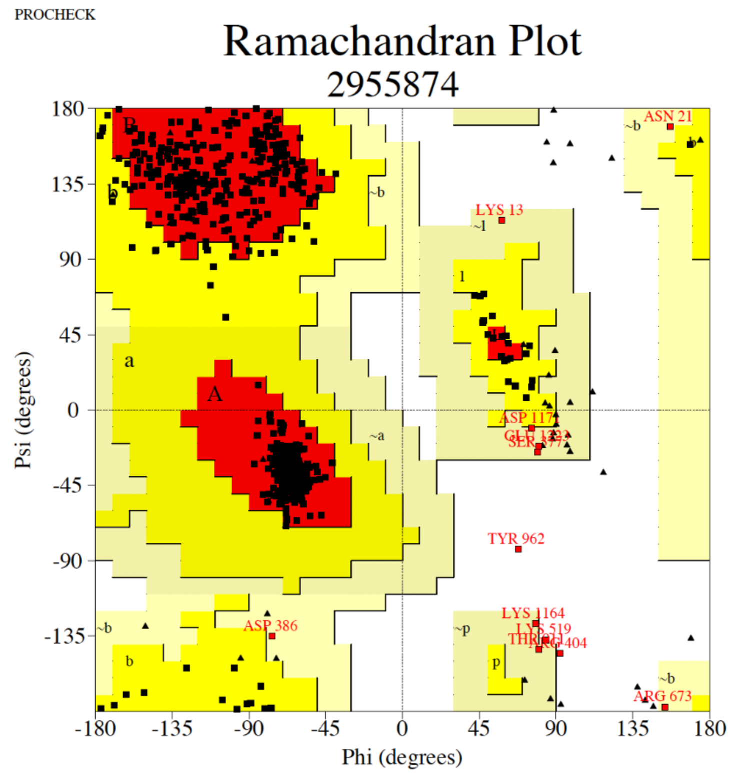


Plot statistics

| | | |
|---|---|---|
| Residues in most favoured regions [A,B,L] | 1083 | 94.2% |
| Residues in additional allowed regions [a,b,l,p] | 55 | 4.8% |
| Residues in generously allowed regions [~a,~b,~l,~p] | 9 | 0.8% |
| Residues in disallowed regions | 3 | 0.3% |
| | ---- | ------ |
| Number of non-glycine and non-proline residues | 1150 | 100.0% |
| Number of end-residues (excl. Gly and Pro) | 2 | |
| Number of glycine residues (shown as triangles) | 99 | |
| Number of proline residues | 29 | |
| | ---- | |
| Total number of residues | 1280 | |

**Fig. S1**. a) Ramachandran plot for the most beneficial B008 homology model (DOPE -153 884.80); b) Ramachandran plot for the model B008 with a linker incorporated[4]

a) b)

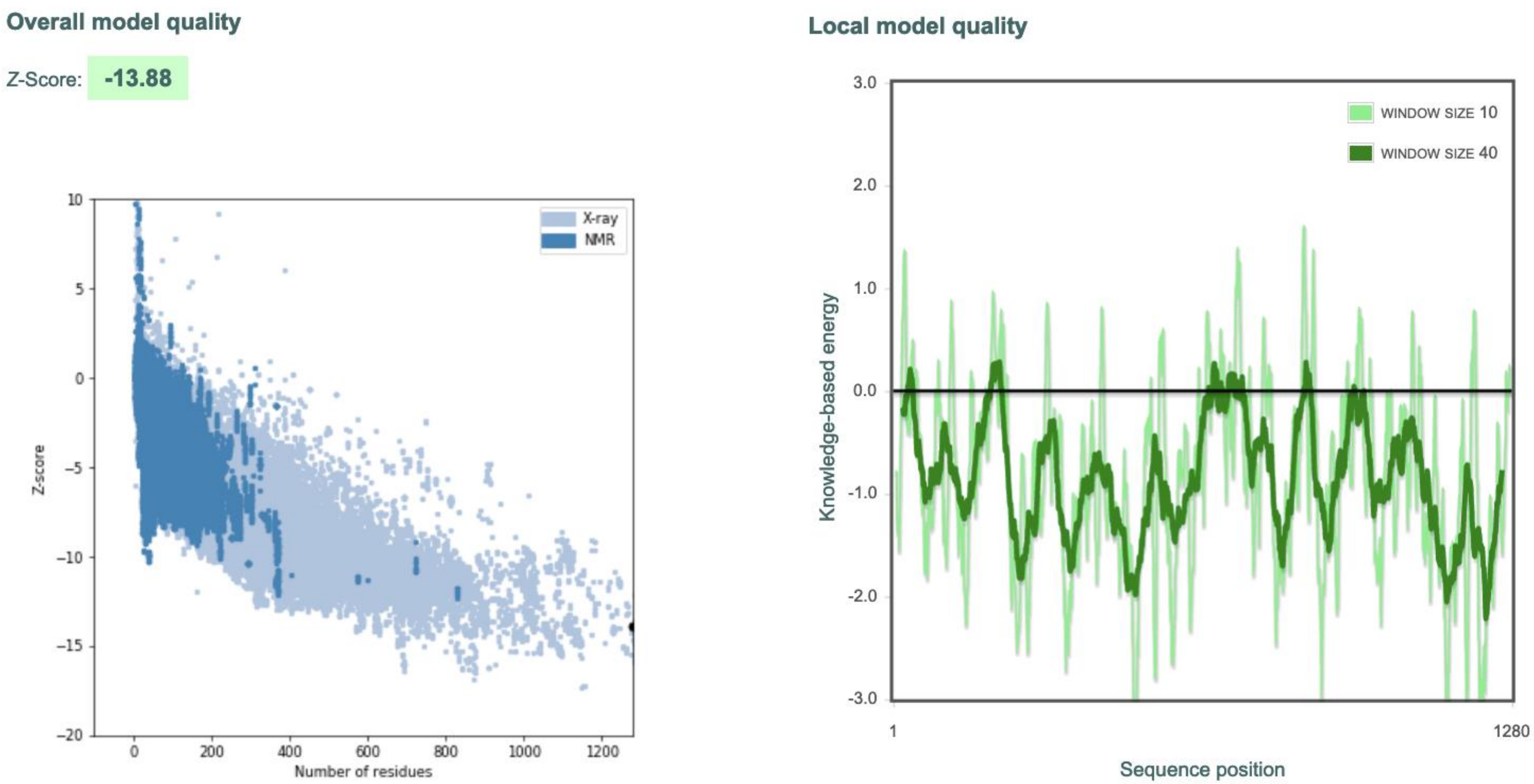


**Fig. S2**. Evaluation of the top-ranked *h*Pgp homology model B008 by ProSA server[5]

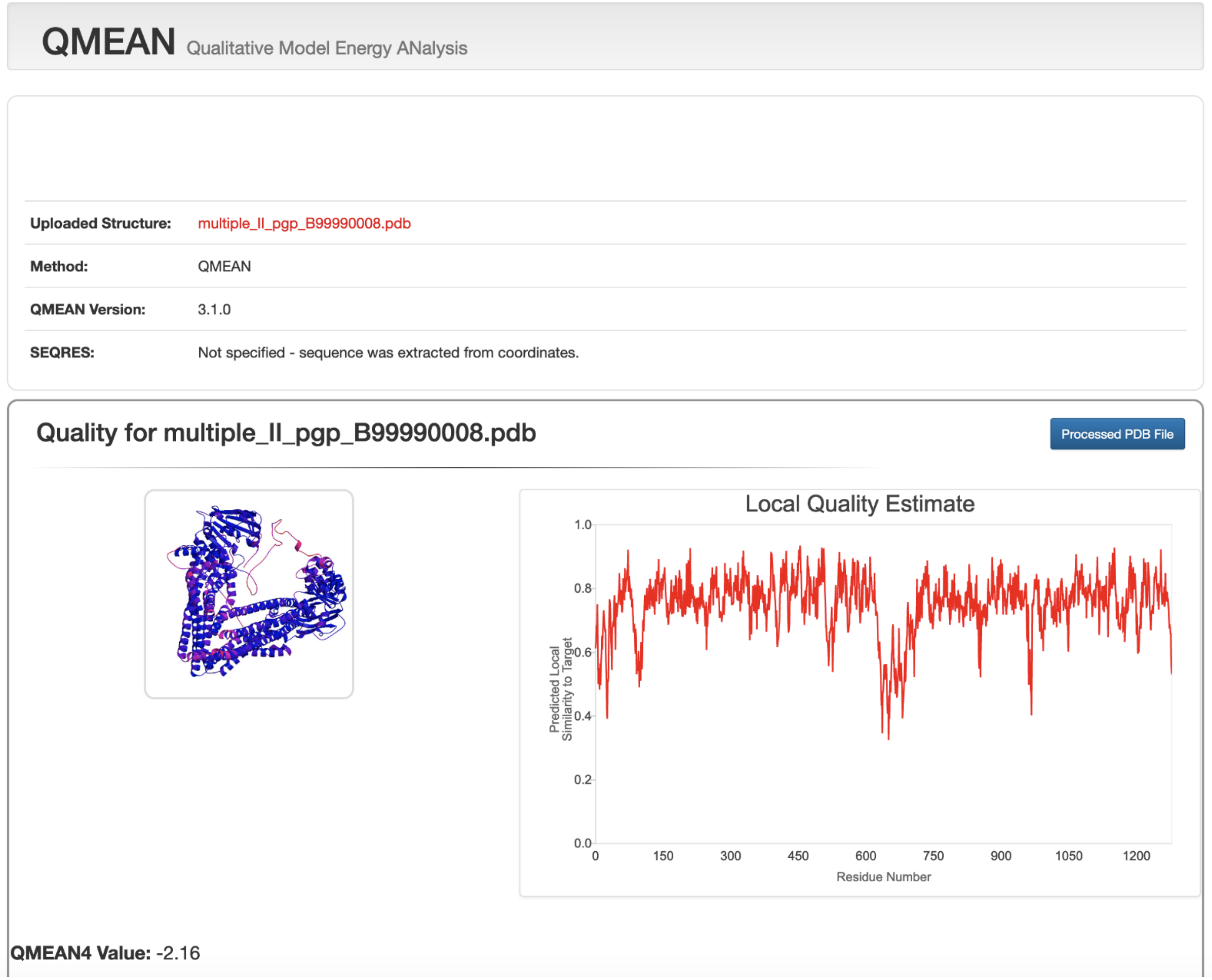


**Fig. S3**. Evaluation of the top-ranked *h*Pgp homology model B008 by QMEAN Swiss Model server[6, 7]

## Docking results

a) Verapamil. Induced fit docking results[2] to the selected homology model of the human Pgp showed similar poses for the neutral and charged forms of verapamil, although docking scores were generally lower for neutral forms of the drug. One of the top-ranked poses (with a docking score -7.471 for the neutral form of verapamil), was chosen as a reference for further investigations for its very good agreement with experimental data (**Fig. S4**, **Table S1**). Three hydrogen bonds were observed in this binding mode: two H-bonds formed by methoxy groups interacting with Tyr 307 and Tyr 953, and a H bond between Tyr 310 and the amine group of verapamil. A very similar binding mode was detected for the pose that showed the highest docking score among the docked protonated forms of verapamil (docking score -10.521, **Table S1**), with the difference that the hydrogen bond is formed between Tyr 310 and the nitrogen atom of the nitrile moiety (**Fig. S4**). Both described poses revealed comparable values of the binding energy expressed by the IFD score (**Table S1**).

**Table S1.** Comparison of docking results for top-ranked poses of the neutral and protonated form of verapamil[2]

| Ligand | Charge | Docking score | XP GScore | IFD Score* | Residues interacting with the ligand within 4 Å |
|---|---|---|---|---|---|
| **Verapamil** | 0 | -7.471 | -10.292 | -2514.52 | Met 69, Phe 72, Ile 306, Tyr 307, Tyr 310, Phe 335, Phe 336, Leu 339, Leu 724, Gln 725, Phe 728, Phe 732, Leu 762, Asn 842, Tyr 953, Phe 957, Leu 975, Phe 978, Ser 979, Phe 983, Met 986 |
| **Verapamil** | +1 | -10.521 | -10.526 | -2512.16 | Met 68, Met 69, Phe 72, Ile 306, Tyr 307, Tyr 310, Phe 336, Leu 339, Phe 343, Gln 725, Phe 728, Phe 732, Phe 759, Met 949, Tyr 953, Phe 978, Val 982, Phe 983, Met 986 |

* IFD score - a scoring function used for estimation of the binding energy for the output pose[2]

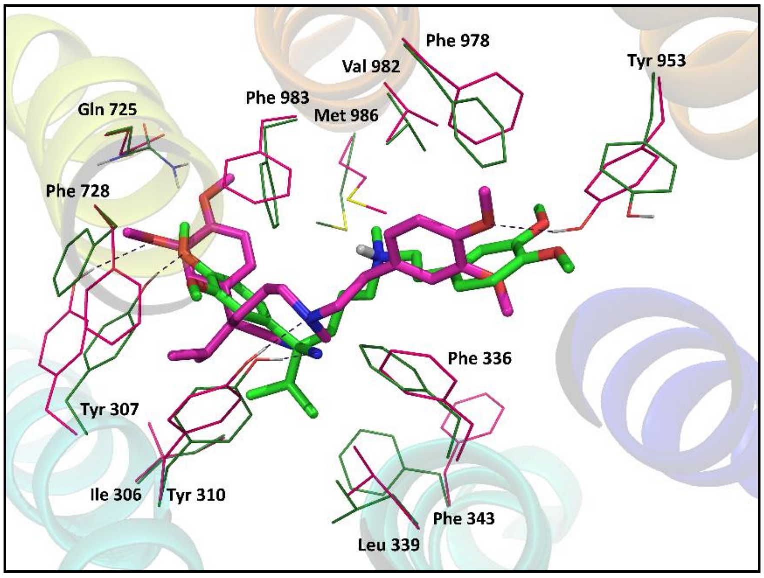


**Fig. S4**. Comparison of the induced fit docking results for the reference pose of verapamil in the neutral form and in the protonated form (top view). The neutral form of verapamil and surrounding residues (side chains only) are marked in pink, the protonated form of the drug and surrounding residues are marked in green. H-bonds between the protein and the ligands are marked by the dashed line.

b) Imidazolone derivatives. Out of docking poses obtained for the neutral forms of compounds, top-ranked poses detected for at least two out of three docked imidazolones were collected in three clusters. The docking scores for individual poses obtained for the compounds **11**, **12** and **18** are presented in **Table S2**:

**Table S2.** Comparison of the induced fit docking results for top-ranked poses of the imidazolone derivatives **11**, **12** and **18** (in the neutral form)[2]

| Ligand | Docking score | XP GScore | IFD Score | Residues interacting with the ligand within 4 Å |
|---|---|---|---|---|
| **pose 1**: cmpd **18** | -12.179 | -13.394 | -2518.72 | Met 68, Met 69, Phe 72, Met 75, Tyr 118, Tyr 307, Tyr 310, Phe 336, Gln 725, Pro 726, Phe 728, Ala 729, Phe 732, Ser 733, Phe 759, Tyr 953, Phe 957, Leu 975, Phe 978, Ser 979, Phe 983, Met 986 |
| **pose 1**: cmpd **12** | -12.170 | -12.581 | -2501.41 | Leu 65, Met 68, Met 69, Phe 72, Tyr 307, Tyr 310, Phe 336, Gln725, Phe 728, Ala 729, Phe 732, Ser 733, Phe 759, Asn 842, Tyr 953, Phe 957, Leu 975, Phe 978, Ser 979, Phe 983 |
| **pose 2**: cmpd **11** | -11.201 | -11.612 | -2520.30 | Met 68, Met 69, Phe 72, Tyr 118, Ile306, Tyr 307, Tyr 310, Leu 332, Phe 335, Phe 336, Leu 339, Phe 728, Ala 729, Phe 732, Ser 733, Ile736, Phe 759, Tyr 953, Phe 957, Leu 975, Phe 978, Ser 979, Phe 983, Met 986 |
| **pose 2**: cmpd **18** | -10.478 | -11,694 | -2518.44 | Met 68, Met 69, Phe 72, Met 75, Tyr 118, Tyr 307, Tyr 310, Phe 335, Phe 336, Phe 728, Ala 729, Phe 732, Ser 733, Ile736, Phe 759, Tyr 953, Phe 957, Leu 975, Phe 978, Ser 979, Phe 983 |
| **pose 2**: cmpd **12** | -10.357 | -10.768 | -2519.14 | Met 68, Met 69, Phe 72, Ile 306, Tyr 307, Tyr 310, Phe 336, Leu 339, Ala 729, Phe 732, Tyr 953, Leu 975, Phe 978, Ser 979, Phe 983, Met 986 |
| **pose 3**: cmpd **11** | -11.869 | -12.280 | -2498.99 | Phe 303, Tyr 307, Tyr 310, Ala 311, Phe 314, Trp 315, Thr 318, Asn 721, Leu 724, Gln 725, Phe 728, Phe 732, Ile 735, Ile 736, Ser 756, Phe 759, Ser 766, Phe 770, Gln 838, Phe 983, Met 986 |
| **pose 3**: cmpd **18** | -10.222 | -11.437 | -2499.17 | Phe 303, Tyr 307, Tyr 310, Phe 314, Phe 336, Asn 721, Gln 725, Phe 728, Ile 731, Phe 732, Ile 735, Phe 759, Ser 766, Phe 770, Gln 838, Phe 983, |

## References:

1. Webb, B.; Sali, A. Protein Structure Modeling with MODELLER. *Methods Mol Biol* **2017,** *1654*, 39-54.
2. Schrödinger Release 2018-1: Protein Preparation Wizard; Induced Fit Docking protocol; Glide; Prime; Schrödinger, LLC, New York, NY, **2018**.
3. Schrödinger Release 2018-1: Maestro; LigPrep; Macromodel; Schrödinger, LLC, New York, NY, **2018**.
4. Laskowski, R. A.; MacArthur, M. W.; Moss, D. S.; Thornton, J. M. PROCHECK: a program to check the stereochemical quality of protein structures. *Journal of Applied Crystallography* **1993,** *26* (2), 283-291.
5. Wiederstein, M.; Sippl, M. J. ProSA-web: interactive web service for the recognition of errors in three-dimensional structures of proteins. *Nucleic acids research* **2007,** *35* (Web Server issue), W407-W410.
6. Benkert, P.; Biasini, M.; Schwede, T., Toward the estimation of the absolute quality of individual protein structure models. *Bioinformatics* **2010,** *27* (3), 343-350.
7. Waterhouse, A.; Bertoni, M.; Bienert, S.; Studer, G.; Tauriello, G.; Gumienny, R.; Heer, F. T.; de Beer, T. A P.; Rempfer, C.; Bordoli, L.; Lepore, R.; Schwede, T. SWISS-MODEL: homology modelling of protein structures and complexes. *Nucleic Acids Research* **2018,** *46* (W1), W296-W303.
